# Utilizing a digital cohort to understand the health burden and lifestyle characteristics across the life course in individuals with polycystic ovary syndrome and possible PCOS

**DOI:** 10.3389/fendo.2025.1585628

**Published:** 2025-09-19

**Authors:** Elizabeth Peebles, Zifan Wang, Erin Dracup, Carrie Sarcione, Christine L. Curry, Kayley Abrams, Jukka-Pekka Onnela, Michelle A. Williams, Russ Hauser, Brent Coull, Shruthi Mahalingaiah

**Affiliations:** ^1^ Department of Environmental Health, Harvard T.H. Chan School of Public Health, Boston, MA, United States; ^2^ Health, Apple Inc., Cupertino, CA, United States; ^3^ Department of Biostatistics, Harvard T.H. Chan School of Public Health, Boston, MA, United States; ^4^ Department of Epidemiology and Population Health, Stanford University, Stanford, CA, United States; ^5^ Department of Epidemiology, Harvard T.H. Chan School of Public Health, Boston, MA, United States

**Keywords:** polycystic ovary syndrome (PCOS), possible PCOS, life course health, infertility, body mass index, lifestyle medicine, age at diagnosis, health burden

## Abstract

**Introduction:**

Polycystic ovary syndrome (PCOS) is an ovulation disorder associated with multiple health conditions. This study analyzed health and lifestyle characteristics of those with diagnosed and possible PCOS in a large, digital cohort.

**Methods:**

We analyzed data from female participants who enrolled in the Apple Women’s Health Study-a mobile-application-based cohort in the United States and provided informed consent from 11/14/2019–12/14/2024. Specific analyses were further restricted to those who responded to relevant survey questions. Self-reported sociodemographic, health (conditions and age at diagnosis), and lifestyle characteristics were evaluated, stratified by PCOS status: PCOS (self-reported physician diagnosed PCOS), possible PCOS (self-reported irregular menses and androgen excess), and no PCOS. Among those with PCOS/possible PCOS, we further evaluated potential predictors of not reporting a PCOS diagnosis using multivariable logistic regression.

**Results:**

Of participants providing medical history at enrollment, 12.6% (n=11,022) reported PCOS, and among the subset without a PCOS diagnosis and with relevant survey data, 17.4% (n=7,152) were assigned possible PCOS. The median baseline age was 35 years. Most participants self-identified as non-Hispanic White (74.2%). The possible PCOS group was slightly less educated (≤high school: possible PCOS 14.5%, PCOS 17.3%, no PCOS 14.0%). The PCOS/possible PCOS groups reported lower socioeconomic status (SES) than the no PCOS group (low SES: PCOS 32.7%, possible PCOS 31.6%, no PCOS 23.5%). The PCOS and possible PCOS groups displayed a high burden of disease (cardiometabolic, endometrial hyperplasia/cancer, pregnancy complications, mental health conditions). Compared to those without PCOS, those with PCOS reported less healthy lifestyle behaviors relevant to physical activity/sleep/stress/smoking and more healthy lifestyle behaviors relevant to alcohol intake/diet. The age at diagnosis for multiple health conditions was earlier for participants with PCOS compared to those without PCOS. Young/old age (18 - 29/40–50 years), lower educational attainment, lower SES, and lower BMI were positive predictors of not reporting a PCOS diagnosis.

**Conclusions:**

This study demonstrated significant differences in health and lifestyle characteristics across PCOS status (PCOS, possible PCOS, no PCOS), identifying populations that could benefit from early risk reduction counseling. Our results may inform discussions around clinical care models through improving awareness of health predictors and lifestyle interventions.

## Introduction

Polycystic ovary syndrome (PCOS) is an ovulation disorder that affects 8 - 13% of the population with some studies estimating over 15% depending on the studied population and criteria (1, 2). PCOS falls into the normo-gonadotropic, normo-estrogenic anovulation ovulation category, defined by the World Health Organization, which captures 85% of ovulation disorders (3). When defined by the 2003 Rotterdam criteria, a PCOS diagnosis requires two out of the three relevant criteria—oligo- or anovulation, clinical and/or biochemical signs of hyperandrogenism, and polycystic ovaries—and excludes other etiologies (congenital adrenal hyperplasia, androgen-secreting tumors, and Cushing’s syndrome) (4).

PCOS is associated with multiple physical and mental health conditions, including metabolic syndrome, infertility, hypertension, obesity, dyslipidemia, diabetes, insulin resistance, non-alcoholic fatty liver, sleep apnea, risk of endometrial hyperplasia or cancer, anxiety, depression, and eating disorders (5, 6). These health conditions can impact an individual with PCOS throughout the life course, starting as early as puberty and into postmenopausal life stages (7). Additionally, there is evidence that some of these comorbidities begin earlier in life for individuals with PCOS compared to those without PCOS (8–11).

In 2020, the economic burden of PCOS in the United States was estimated at approximately $3.7 billion (including initial diagnosis and reproductive endocrine morbidities). When accounting for the cost of pregnancy-related and long-term morbidities, the economic burden rose to $4.3 billion annually (12). Delays in diagnoses can contribute to this cost. In a global-reaching survey disseminated to individuals with PCOS via online support groups, over one third of respondents (33.6%) reported taking over two years to receive a diagnosis and almost half of respondents (47.1%) saw three or more health care professionals before receiving a diagnosis (13).

There is also a high level of patient dissatisfaction with receiving a PCOS diagnosis and healthcare experiences (13–17). Particularly, patients are not satisfied with provided information on medical therapy and lifestyle intervention after receiving a diagnosis (13), which aligns with trends of self-education often through the internet (14, 18). In another study of over 750 individuals with PCOS, more than half of the respondents (57.3%) were dissatisfied with the overall medical care they received for their PCOS diagnosis (14).

Delays in diagnoses and patient dissatisfaction pertain to individuals who received a PCOS diagnosis; however, up to 70% of affected individuals remain undiagnosed (1). Few studies exist, to our knowledge, that include individuals who meet diagnostic criteria of PCOS but have not received a formal diagnosis. In line with existing literature (9, 19–22), we refer to this group as “possible PCOS”, but previous studies use other labels such as “probable PCOS”, “algorithm PCOS”, and “study diagnosis” (9, 19–24).

Lifestyle medicine is a field of healthcare that uses interventions to better overall health. The field is designed around using education and behavioral changes as treatment options to prevent, manage, and reverse several chronic diseases including PCOS (25). The six pillars of lifestyle medicine are nutrition, physical activity, stress management, sleep, social connections, and avoiding risky substances (26). These lifestyle behaviors are important to assess in individuals with PCOS and possible PCOS to help clinicians better understand the full health profile of their patients and provide appropriate screening, counseling, and care.

**Figure 1:**
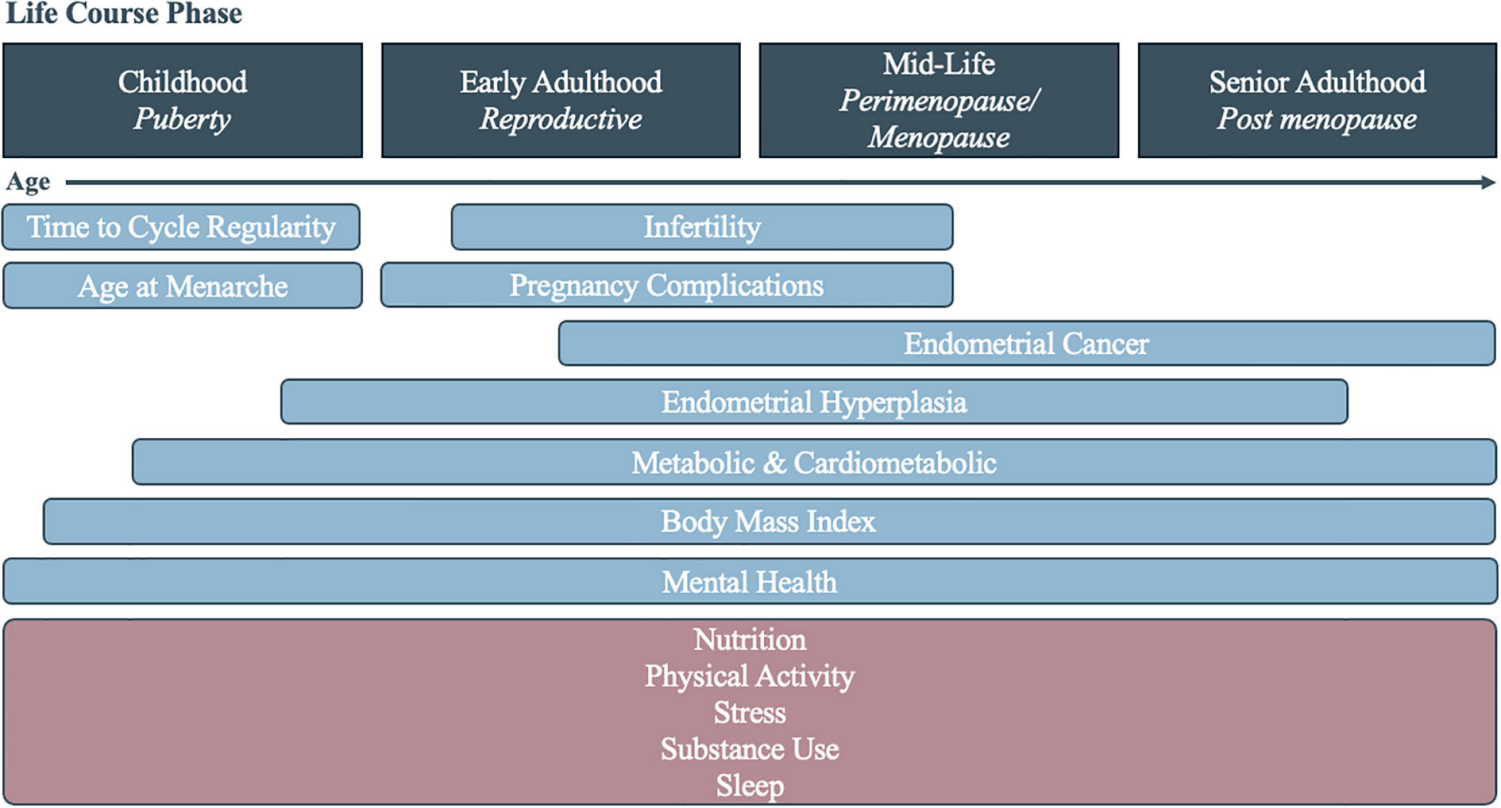
Diagram of the assessed health (blue) and lifestyle (pink) characteristics associated with polycystic ovary syndrome across the life course.

There is a clear need to better understand the lifecourse implications of PCOS in all facets of life. This includes understanding sociodemographic and lifestyle characteristic differences for those with a diagnosis and for those with possible PCOS, who present the clinical symptoms but have not been diagnosed. By doing so, systems of care can better address the delays and missing diagnoses that PCOS patients experience. In this study, we examined three distinct dimensions—sociodemographic, health, and lifestyle characteristics—across the life course of individuals with PCOS, possible PCOS, and without PCOS in a large, demographically diverse United States cohort (**Figure 1**). By analyzing the timing of disease diagnoses and identifying key factors associated with reporting a PCOS diagnosis, we aim to reveal patterns that may inform clinical practices to improve both early detection and long-term patient outcomes.

## Materials and method

### Study design and population

The Apple Women’s Health Study (AWHS) is a prospective digital application-based cohort study in the United States. Recruitment began 11/14/2019 and is ongoing. Participants are eligible to enroll through the Apple Research app on an iPhone if they live in the United States, are age 18 or older (19 in Alabama and Nebraska, 21 in Puerto Rico), have menstruated at least once, are able to communicate in English, are the sole user of their iCloud account, and provide written informed consent at enrollment. More details on the AWHS can be found in previous publications (27). This study was approved by the Institutional Review Board at Advarra (CIRB #PRO00037562).

For this cross-sectional analysis, we included data from 50,357 female participants who enrolled in the AWHS from 11/14/2019 to 12/14/2024 and responded to relevant survey questions on self-reported sociodemographic, health, and lifestyle characteristics. Surveys were distributed at enrollment and updated annually (except for the reproductive history survey, which was distributed once at enrollment). For each survey, we restricted to participants’ single response at enrollment or their first response during the study, whichever was earliest (“baseline”). Those who indicated “I don’t know” (<1% of participants) or “I prefer not to answer” as survey responses or did not respond were excluded from the relevant analyses. A flowchart depicting inclusion/exclusion can be found in **Supplementary Figure S1**. Survey question details can be found in **Supplementary Table S1**.

### PCOS and possible PCOS

We defined PCOS as selecting “polycystic ovary syndrome (PCOS)” to the question, “Have you ever been diagnosed with any of the following by a doctor or other care provider? (select all that apply).”

We defined possible PCOS among participants who did not report a PCOS diagnosis and responded to survey questions relevant to further identifying possible PCOS using the following criteria: those who did not self-report a PCOS diagnosis but had both androgen excess at enrollment and a history of irregular cycles from self-reported characteristics (i.e., meeting two of the three Rotterdam criteria) (4). Based on available data in this cohort, androgen excess was defined as having hirsutism or moderate/severe acne. Hirsutism was defined as those who reported thick, coarse, and dark hair growth on the body, reported “several”/“a lot” of hair growth on the upper lip, or reported “several”/“a lot” of hair growth on the chin. Moderate/severe acne was defined as those who reported having “red, irritated pimples” or “pimples with pus” (28). Based on available data, irregular cycles assignment was based on either reporting time from menarche to cycle regularity as 5+ years, not yet regular, or regular after hormones (11) or reporting less than 8 cycles in a typical year (29) for those who were not currently using hormones at baseline. Possible PCOS represents individuals with a possible missed diagnosis.

Among the remaining participants, those who did not self-report a PCOS diagnosis and did not meet the definition of possible PCOS in this cohort were classified as no PCOS.

A reported family history of PCOS was recorded by the participant answering, “Have your biological mother, sister, or daughter ever been diagnosed with any of the following?” with “polycystic ovarian syndrome (PCOS)” as an option.

### Sociodemographic characteristics

Participant sociodemographic characteristics were self-reported via survey questions. Age was calculated from provided date of birth. Participant race and ethnicity was self-reported and categorized as Asian, Hispanic, non-Hispanic Black, non-Hispanic White, and multiple races (self-identified >1 option)/other races, where “other races” represented several infrequently chosen groups (American Indian or Alaska Native/Middle Eastern or North African/Native Hawaiian or Pacific Islander/“None of these fully describe me”). Educational status was reported as high school or below, some college or technical school, college degree, or graduate degree. Socioeconomic status (SES) was assessed using the MacArthur Scale of Subjective Social Status where participants reported their self-rated rank on a social ladder relative to others (0 – 3 [low], 4 – 5 [medium], 6 – 9 [high]) (30).

### Health characteristics

We defined age at menarche using the participant’s answer to, “At what age did you have your first menstrual period?” with the answer options of “7 years old or younger”, “8 years old”, “9 years old”, “10 years old”, “11 years old”, “12 years old”, “13 years old”, “14 years old”, “15 years old”, “16 years old or older”, “I don’t know”, and “I prefer not to answer”. Responses of “I don’t know” or “I prefer not to answer” were considered as missing values. Participants were grouped into very early menarche (<9 years old), early menarche (<11 years old), and late menarche (≥16 years old).

Time to cycle regularity was evaluated by the participant’s answer to “After your first menstrual cycle, how long did it take for your cycle to become regular?” with the answer options of “less than 1 year”, “1–2 years”, “3–4 years”, “more than 5 years”, “after using hormone (i.e., birth control pills)”, “they’re not yet regular”, “I don’t know”, and “I prefer not to answer”. Responses of “I don’t know” or “I prefer not to answer” were considered as missing values.

Infertility was assigned to participants that selected “infertility” to the question “Have you ever been diagnosed with any of the following by a doctor or other care provider? Select all that apply.” Participant gravidity was reported as the answer to the question, “How many times have you been pregnant?”. For those that replied ≥1 and agreed to share pregnancy history information, data from their first pregnancy were used to evaluate the use of assisted reproductive technology (ART) and pregnancy complications. To assess ART used, participants were asked, “Did you conceive with help of any of these methods? Select all that apply” for each reported pregnancy with options of artificial insemination, medication, and *in vitro* fertilization (IVF). Selecting “No” assigned the participant to the “conceived naturally” category. Pregnancy complications were evaluated by the question, “Did you have any complications related to this pregnancy? Select all that apply” with answer options of “Gestational diabetes (diabetes only during pregnancy)”, “Gestational hypertension (high blood pressure only during pregnancy)”, “Preeclampsia or eclampsia (high blood pressure leading to other complications)”, “Heart problems (heart failure or heart attack)”, “Postpartum hemorrhage (very heavy bleeding during delivery)”, “Anemia (low blood count in pregnancy)”, “Placental abruption (separation of the placenta from uterus)”, “Placenta previa (placenta covering the cervix)”, “Intrauterine growth restriction (fetus was too small for weeks of pregnancy)”, “Perinatal depression (depression during or after pregnancy)”, “Hysterectomy (surgery to remove your uterus)”, “Severe infection or sepsis”, “Seizure disorder”, “None of the above”, and “I prefer not to answer”. Responses of “I prefer not to answer” were considered as missing values.

Metabolic and cardiometabolic conditions were collected by participants answering, “Have you ever been diagnosed with any of the following by a doctor or other care provider? Select all that apply” with the options of “prediabetes”, “type 2 diabetes”, “arrhythmia, such as atrial fibrillation (Afib) or atrial flutter”, “congestive heart failure”, “coronary artery disease (CAD)”, “heart attack”, “high cholesterol”, “hypertension (high blood pressure)”, “stroke”, “transient ischemic attack (mini-stroke)”, “none of the above”, and “I prefer not to answer.” Responses of “I don’t know” or “I prefer not to answer” were considered as missing values.

Similarly, endometrial cancer and endometrial hyperplasia were assessed by participants answering, “Have you ever been diagnosed with any of the following by a doctor or other care provider? Select all that apply” with “endometrial cancer (cancer of uterus)” and “endometrial hyperplasia (pre-cancer of uterus)” as options. Responses of “I prefer not to answer” were considered as missing values.

Body mass index (BMI) at enrollment was calculated from self-reported weight and height at baseline and further categorized as <18.5 (underweight), 18.5 - 24.9 (healthy weight), 25 - 29.9 (overweight), 30.0-34.9 (obesity class 1), 35.0 - 39.9 (obesity class 2), and ≥40 kg/m^2^ (obesity class 3) following Center for Disease Control and Prevention’s (CDC) cutoff values (31).

In August 2023, a set of survey questions were added to the baseline reproductive history survey, asking participants to recall their weight at different ages (18, 25, 35, 45, and/or 55 years old, depending on their age at enrollment) and their height at age 18. Among all 50,357 participants in this analysis (**Table 1**), 5,031 participants provided weight and height information at age 18 (numbers for other timepoints shown in **Table 1**). All data were transformed to weight kilogram (kg) and height in kilogram (kg). We calculated BMI at each timepoint (weight at each timepoint in kg/[height at age 18 in meters]^2^) among the individuals who provided this information while acknowledging the limitation of the high missingness rate for BMI history data.

**Table 1:** Baseline sociodemographic and health characteristics of 50,357 Apple Women’s Health Study participants.

Characteristics self-reported at baseline^a^	Overall	By PCOS status		
		PCOS	Possible PCOS	No PCOS
N	50,357	11,022	7,152	32,183
Age, years, median (IQR)	35 (27 – 43)	33 (27 – 40)	33 (25 – 43)	36 (28 – 44)
Age category, n (%)				
18-19	1837 (3.6)	240 (2.2)	341 (4.8)	1256 (3.9)
20-29	14420 (28.6)	3317 (30.1)	2501 (35.0)	8602 (26.7)
30-39	16244 (32.3)	4207 (38.2)	1977 (27.6)	10060 (31.3)
40-49	11237 (22.3)	2199 (20.0)	1215 (17.0)	7823 (24.3)
≥50	6209 (12.3)	649 (5.9)	1118 (15.6)	4442 (13.8)
Race and ethnicity,^b^ n (%)				
Non-Hispanic White	37369 (74.2)	7901 (71.7)	5420 (75.8)	24048 (74.7)
Non-Hispanic Black	2320 (4.6)	435 (3.9)	266 (3.7)	1619 (5.0)
Asian	1447 (2.9)	345 (3.1)	128 (1.8)	974 (3.0)
Hispanic	3197 (6.3)	807 (7.3)	427 (6.0)	1963 (6.1)
Multiple/other races	5887 (11.7)	1500 (13.6)	893 (12.5)	3494 (10.9)
Education level, n (%)				
High school or below	7348 (14.6)	1603 (14.5)	1239 (17.3)	4506 (14.0)
Some college or tech school	15929 (31.6)	4065 (36.9)	2354 (32.9)	9510 (29.5)
College degree	14899 (29.6)	3069 (27.8)	2043 (28.6)	9787 (30.4)
Graduate degree	12017 (23.9)	2256 (20.5)	1486 (20.8)	8275 (25.7)
Subjective socioeconomic status (SES), n (%)				
Low (0-3)	13428 (26.7)	3605 (32.7)	2262 (31.6)	7561 (23.5)
Medium (4-5)	21027 (41.8)	4718 (42.8)	2904 (40.6)	13405 (41.7)
High (6-9)	15806 (31.4)	2675 (24.3)	1974 (27.6)	11157 (34.7)
Reported family history of PCOS, n (%)	4744 (9.4)	2654 (24.1)	481 (6.7)	1609 (5.0)
Body mass index, kg/m^2^, median (IQR)	27.9 (23.4 – 34.1)	32.4 (26.3 – 39.0)	27.5 (23.0 – 33.6)	26.6 (22.9 – 32.3)
Body mass index, kg/m^2^, n (%)				
Underweight (<18.5)	1213 (2.4)	165 (1.5)	226 (3.2)	822 (2.6)
Healthy weight (18.5-24.9)	15906 (31.6)	1959 (17.8)	2332 (32.6)	11615 (36.1)
Overweight (25.0-29.9)	12438 (24.7)	2169 (19.7)	1788 (25.0)	8481 (26.4)
Obesity 1 (30.0-34.9)	8812 (17.5)	2244 (20.4)	1214 (17.0)	5354 (16.6)
Obesity 2 (35.0-39.9)	5564 (11.0)	1854 (16.8)	765 (10.7)	2945 (9.2)
Obesity 3 (≥40)	5458 (10.8)	2394 (21.7)	678 (9.5)	2386 (7.4)

IQR, interquartile range.

a Numbers and percentages may not add up to total N or 100% due to missingness. Missingness rates were <1% for sociodemographic characteristics, and <2% for body mass index at baseline and family history of PCOS.

b Those who self-identified categories of “American Indian or Alaska Native”, “Middle Eastern or North African”, “Native Hawaiian or Pacific Islander”, or “none of these fully describe me” were collapsed as “other races” due to relatively small sample sizes. Multiple races included those who self-identified more than one option.

Mental health conditions were assessed by participants’ responses to, “Have you ever been diagnosed with any of the following by a doctor or other care provider? Select all that apply” with the options of, “Anorexia”, “Anxiety disorder”, “Attention deficit & hyperactivity disorder (ADHD)”, “Bipolar disorder”, “Bulimia”, “Depression”, “Panic disorder”, “None of the above”, and “I prefer not to answer.” Responses of “I prefer not to answer” were considered as missing values.

To assess overall health, participants were asked, “How would you describe your health compared to other people your age?” with the options of, “Much better”, “Slightly better”, “About the same”, “Slightly worse”, “Much worse”, and “I prefer not to answer”.

For all relevant health conditions, age at diagnosis was asked by the participant entering the age (integer) in years to answer the question, “How old were you when you were diagnosed with this condition? It’s okay to estimate.”

### Lifestyle characteristics

Nutrition was evaluated whether the participant reported following a diet (“Do you follow a special diet? Select all that apply”) with options of “Low calorie”, “Low carb”, “Low fat”, “High fat”, “High protein”, “Low sodium”, “Vegetarian”, “Vegan”, “No gluten”, “No dairy”, “Other special diet”, “No special diet”, and “I prefer not to answer”. Additionally, participants were asked, “In the past calendar month, how frequently did you eat fruits and vegetables?” with options of, “Fewer than 3 times a week”, “4–7 times a week”, “8–14 times a week”, “15 or more times a week”, and “I prefer not to answer”.

Physical activity was assessed by exercise minutes per week (“How much exercise do you usually get per week? Include any moderate to vigorous leisure time activity, such as brisk walking, running, cycling, dancing, strength training, or playing soccer.”) with options of, “none”, “1–75 minutes”, “76–150 minutes”, “151–300 minutes”, “>300 minutes”, and “I prefer not to answer.” Exercise duration was categorized into two groups: “≥151 minutes” and “≤150 minutes” per week. This classification was based on the recommendations of the *Physical Activity Guidelines for Americans (PAG)*, which advocate for at least 150 minutes of physical activity weekly for adults (32).

A self-assessment of overall physical activity level was also asked (“How would you describe your overall physical activity level? Select all that apply”), with “I don’t do any physical activity”, “I participate in light activities, such as walking or light housework”, “I participate in moderate activities, such as brisk walking or yard work”, “I participate in vigorous activities, such as running or carrying heavy loads”, “I participate in strenuous activities, such as competitive sports or endurance events like marathons”, and “I prefer not to answer” as response options.

Stress levels were assessed by four questions using the Perceived Stress Scale 4 (PSS - 4), asking participants about their feelings and thoughts during the past month. Following the standard scoring instructions (33), we determined the total PSS - 4 score by adding together the scores of each of the four questions. Specifically, “never”, “almost never”, “sometimes”, “fairly often”, and “very often” were coded as 0, 1, 2, 3, 4 respectively, for the questions “In the last month, how often have you felt that you were unable to control the important things in your life?” and “In the last month, how often have you felt difficulties were piling up so high that you could not overcome them?”, and responses were reversely coded as 4, 3, 2, 1, 0 respectively, for the questions “In the last month, how often have you felt confident about your ability to handle your personal problems?” and “In the last month, how often have you felt that things were going your way?”. Indicating “I prefer not to answer” or not responding to any of the four questions were considered as missing values.

Substance use was assessed by asking questions about alcohol, tobacco, e-cigarette, and marijuana use. Alcohol consumption was evaluated on frequency (“How often do you have a drink containing alcohol in the past year?”) with options of “Never”, “Monthly or less”, “Two to four times a month”, “Two to three times a week”, “Four or more times a week”, and “I prefer not to answer”, and by quantity (“On a typical day when you drink, how many drinks do you have?”) with options of “1 or 2”, “3 or 4”, “5 or 6”, “7 to 9”, “10 or more”, and “I prefer not to answer”. A combined alcohol variable (categorized by “No or light alcohol consumption”, “light consistent or rare binge drinker”, and “moderate-to-heavy consistent drinker or frequent binge drinker”) was created based on The National Institute on Alcohol Abuse and Alcoholism’s (NIAAA) definition of heavy drinking (4 or more drinks/day or 8 or more drinks/week for women). This variable accounts for both frequency (number of times in a month) and quantity (drinks/day) (34).

Tobacco use was defined by responses to questions regarding cigarette smoking habits (“Have you smoked at least 100 cigarettes in your entire life?”). Participants who responded “No” were designated as “Never smokers,” aligning with the CDC’s definition of individuals who have smoked fewer than 100 cigarettes in their lifetime (35). Participants who answered “Yes” were subsequently asked, “Do you now smoke cigarettes every day, some days, or not at all?” with available responses being “Every day,” “Some days,” “Not at all,” and “I prefer not to answer.” Those who selected “Every day” or “Some days” were classified as “Current smokers,” while respondents who answered, “Not at all,” were categorized as “Past smokers.”

E-cigarette use was assessed by asking, “Do you now use electronic nicotine products every day, some days or not at all?”, with options of, “Every day”, “Some days”, “Not at all”, and “I prefer not to answer”. Those who indicated never using e-cigarette were merged into the “Not at all” group. Similarly, the frequency of marijuana use was captured by asking, “How often do you currently use marijuana in any form?”, with options of, “Every day”, “Some days”, “Not at all”, and, “I prefer not to answer”. Those who indicated never using marijuana were merged into the “Not at all” group.

Sleep was assessed by asking participants the following questions, “What time do you usually (fall asleep/wake up) on (weekdays or workdays/weekends or non-workdays)?” Typical sleep durations were then calculated by the hour difference between the reported asleep and wake up times. Sleep apnea/breathing disturbances were assessed via two questions. One question asked, “Have you ever been diagnosed with any of the following by a doctor or other care provider?” with “sleep apnea” as one of the options. Another question asked, “In the past 12 months, how often did you snort, gasp, or stop breathing while you were asleep?”, with “never”, “rarely (1 – 2 nights a week)”, “occasionally (3 – 4 nights a week)”, “frequently (5 or more nights a week)”, “I don’t know”, and “I prefer not to answer” as response options. Responses of “I prefer not to answer” were considered as missing values. Responses of “I don’t know” were preserved as one of the response categories for this question.

### Statistical analysis: main analysis

We summarized the baseline sociodemographic characteristics, BMI, and family history of PCOS, among all 50,357 participants, as well as by PCOS/possible PCOS status, calculating median (interquartile range [IQR]) for continuous variables and number (percentages) for binary/categorical variables. For all health and lifestyle characteristics, we calculated the mean (SD) for continuous variables and number (percentages) for binary/categorical variables, stratified by PCOS/possible PCOS/no PCOS groups. The 95% confidence intervals (CIs) of the mean or percentage values were further calculated as a measure of degree of uncertainty for the estimated population proportions within each group. Overall differences across the PCOS/possible PCOS/no PCOS groups were evaluated using Chi-square tests (for binary/categorical variables) and Kruskal-Wallis tests (for continuous variables).

### Statistical analysis: secondary and sensitivity analyses

In a secondary analysis, we compared those with possible PCOS to those with PCOS by fitting all baseline sociodemographic characteristics, BMI, and family history of PCOS in a logistic regression with possible PCOS as the outcome. Covariates with a positive odds ratio were considered to be potential predictors for not reporting having received a PCOS diagnosis despite presenting relevant symptoms that meet the diagnostic criteria.

Additionally, we conducted a sensitivity analysis to better understand the participants who did not respond to the two surveys relevant to our possible PCOS definition (subests of participants shown in study flowchart, **Supplementary Figure S1**). We compared baseline sociodemographic and health characteristics of participants who completed the medical history survey but did not report a PCOS diagnosis (n=75,931; Subset 1); participants who completed the medical history and the reproductive history surveys (n=72,533; Subset 2); and participants who completed the medical history, reproductive history, and hormonal symptoms surveys (n=41,197; Subset 3).

In the AWHS participants can choose to link their clinical health records to the Health app and can consent to share this data with the Research app. We ran a sensitivity analysis comparing the PCOS-relevant lab values by PCOS status to test the robustness of our analysis (total testosterone [T] in serum/plasma, free T in serum/plasma, follicle-stimulating hormone [FSH] in serum/plasma, luteinizing hormone [LH] in serum/plasma, estradiol [E2] in serum/plasma, thyroid-stimulating hormone [TSH] also known as thyrotropin in serum/plasma, prolactin in serum/plasma, 17-hydroxyprogesterone [17-OHP] in serum/plasma, dehydroepiandrosterone sulfate [DHEA-S] in serum/plasma, hemoglobin A1c [HbA1c] in blood, and high-density lipoprotein [HDL] in serum/plasma).

Previous research and recommendations from the World Health Organization has suggested that the BMI cutoffs used for the general population may not be sufficient for Asian populations. Rather, the obesity cut off for Asian Americans should be 27 kg/m^2^ as opposed to 30 kg/m^2^ (36, 37). Therefore, we ran a sensitivity analysis of BMI at baseline and at the 5 age assessment points with the Asian population BMI cutoff thresholds (underweight: <18.5 kg/m^2^, healthy weight 18.5 - 22.9 kg/m^2^, overweight: 23.0 - 26.9 kg/m^2^, obesity: ≥27.0 kg/m^2^).

To further understand participants’ characteristics across the life course and explore whether these variations may explain life course BMI variations across different age groups in this cohort, we also evaluated sociodemographic characteristics and overall health among all participants stratified by age groups. Furthermore, to account for the potential age or birth cohort impacts on behavioral and lifestyle factors, we summarized the lifestyle characteristics by PCOS/possible PCOS status within each age group. These age-stratified sensitivity analyses were conducted to explore overall trends and assess the robustness of our main findings. As such, we did not perform Chi-square or Kruskal-Wallis tests on these sensitivity analyses.

Data processing and statistical analyses were conducted in Python (version 3.6) and R (version 4.1.2). All statistical tests were 2-sided. Results with P-values <.05 were considered statistically significant. Significant results with relatively large magnitude or clinical relevance are discussed in the main text.

## Results

### Sociodemographic characteristics

Among the 87,487 participants who completed the medical history survey at enrollment, 12.6% (n=11,022) reported physician diagnosed PCOS, and among the analyzed subset without a PCOS diagnosis and with reproductive history and hormone symptom data (n=41,197), 17.4% (n=7,152) were assigned possible PCOS (**Table 1**; **Supplementary Figure S1**). The median (IQR) age at baseline of the cohort was 35 (27 - 43) years, and those with PCOS and possible PCOS reported a moderately lower median age at baseline than those without PCOS (PCOS: 33 [27 - 40] years; possible PCOS: 33 [25 - 43] years; no PCOS: 36 [28 - 44] years; **Table 1**). A total of 6,209 participants were over 50 years old at enrollment. Those with possible PCOS tended to be either younger (≤29 years) or older (≥50 years) at baseline (39.8% ≤29 years; 15.6% ≥50 years) compared to those with PCOS (32.3% ≤29 years; 5.9% ≥50 years) and without PCOS (30.6% ≤29 years; 13.8% ≥50 years). Those with PCOS reported age at baseline within early adulthood (38.2% 29 – 39 years) more than those with presumed PCOS (27.6%) and without PCOS (31.3%).

The majority of participants were non-Hispanic White (74.2%) which was similar when stratified by PCOS status. Additionally, most participants had received some level of post-high school education (85.1% some college/tech school or higher). Those with possible PCOS reported high school or below as the highest level of education obtainment (17.3%) more than those with PCOS (14.5%) or without PCOS (14.0%). Those without PCOS also reported earning a graduate degree (25.7%) more than those with PCOS (20.5%) or possible PCOS (20.8%).

Participants with PCOS reported a low subjective SES the most (32.7%), followed by participants with possible PCOS (31.6%), then participants without PCOS (23.5%). The inverse trend applies to high subjective SES; those with PCOS reported a high subjective SES the least (24.3%), followed by participants with possible PCOS (27.6%), then participants without PCOS (34.7%).

Lastly, almost a quarter of participants with PCOS (24.1%) reported a family history of PCOS as compared to 6.7% and 5.0% for participants with possible PCOS and without PCOS, respectively.

### Health characteristics

#### Overall

**Figure 2:**
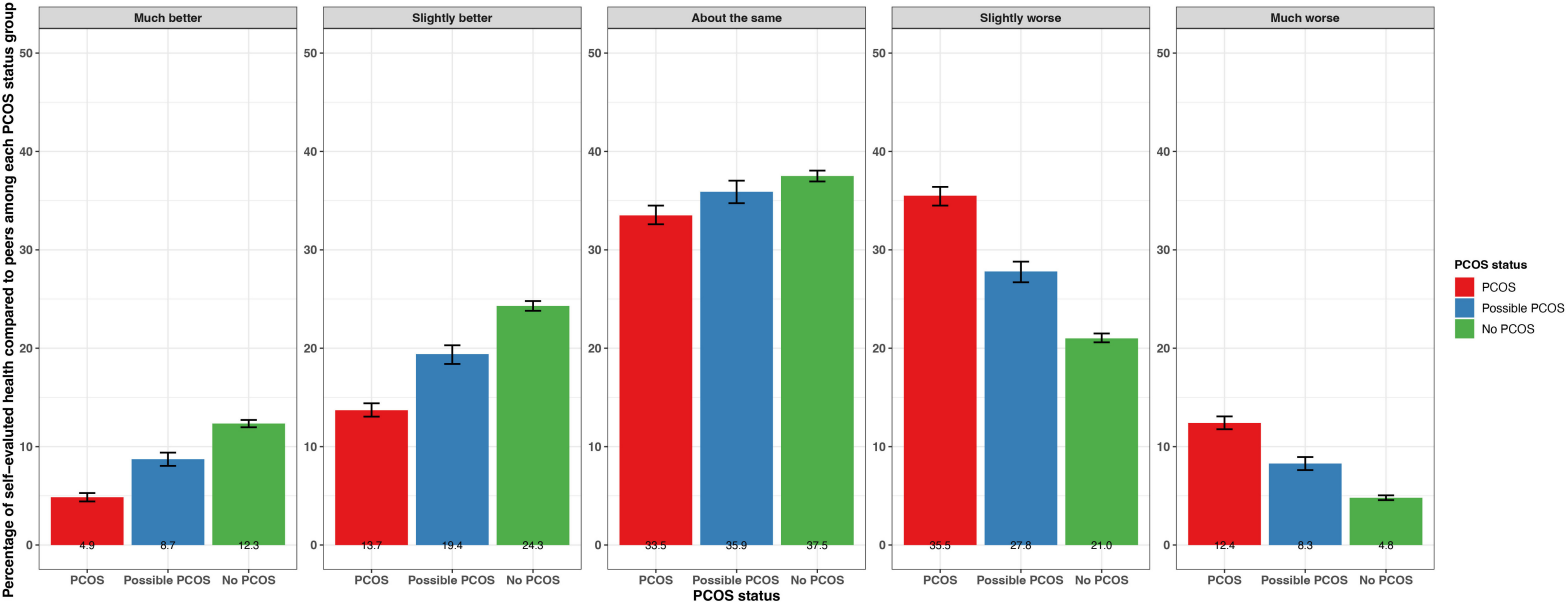
Percentage of self-evaluated overall health compared to others of the same age with 95% confidence intervals among AWHS participants, stratified by PCOS status (PCOS, possible PCOS, no PCOS). Error bars represent 95% confidence intervals. AWHS, Apple Women’s Health Study; PCOS, polycystic ovary syndrome.

When asked about overall health compared to others their age, participants with PCOS and possible PCOS reported “much worse” (PCOS: 12.4% [95% CI 11.8 - 13.1%], possible PCOS: 8.3% [95% CI 7.6 - 8.9%]) and “slightly worse” (PCOS: 35.5% [95% CI 34.5 - 36.4%], possible PCOS: 27.8% [95% CI 26.7 - 28.8%]) more frequently than participants without PCOS (much worse: 4.8% [95% CI 4.6 - 5.1%], slightly worse: 21.0% [95% CI 20.6 - 21.5%]). Participants without PCOS reported an overall health “slightly better” (24.3% [95% CI 23.8 - 24.8%]), and “much better” (12.3% [95% CI 12.0 - 12.7%]) than others their age more frequently than participants with PCOS (slightly better: 13.7% [95% CI 13.1 - 14.4%], much better: 4.9% [95% CI 4.4 - 5.3%]) or possible PCOS (slightly better: 19.4% [95% CI 18.4 - 20.3%], much better: 8.7% [95% CI 8.0 - 9.4%]) (**Figure 2**; **Supplementary Table S2**).

#### Menarche

**Figure 3:**
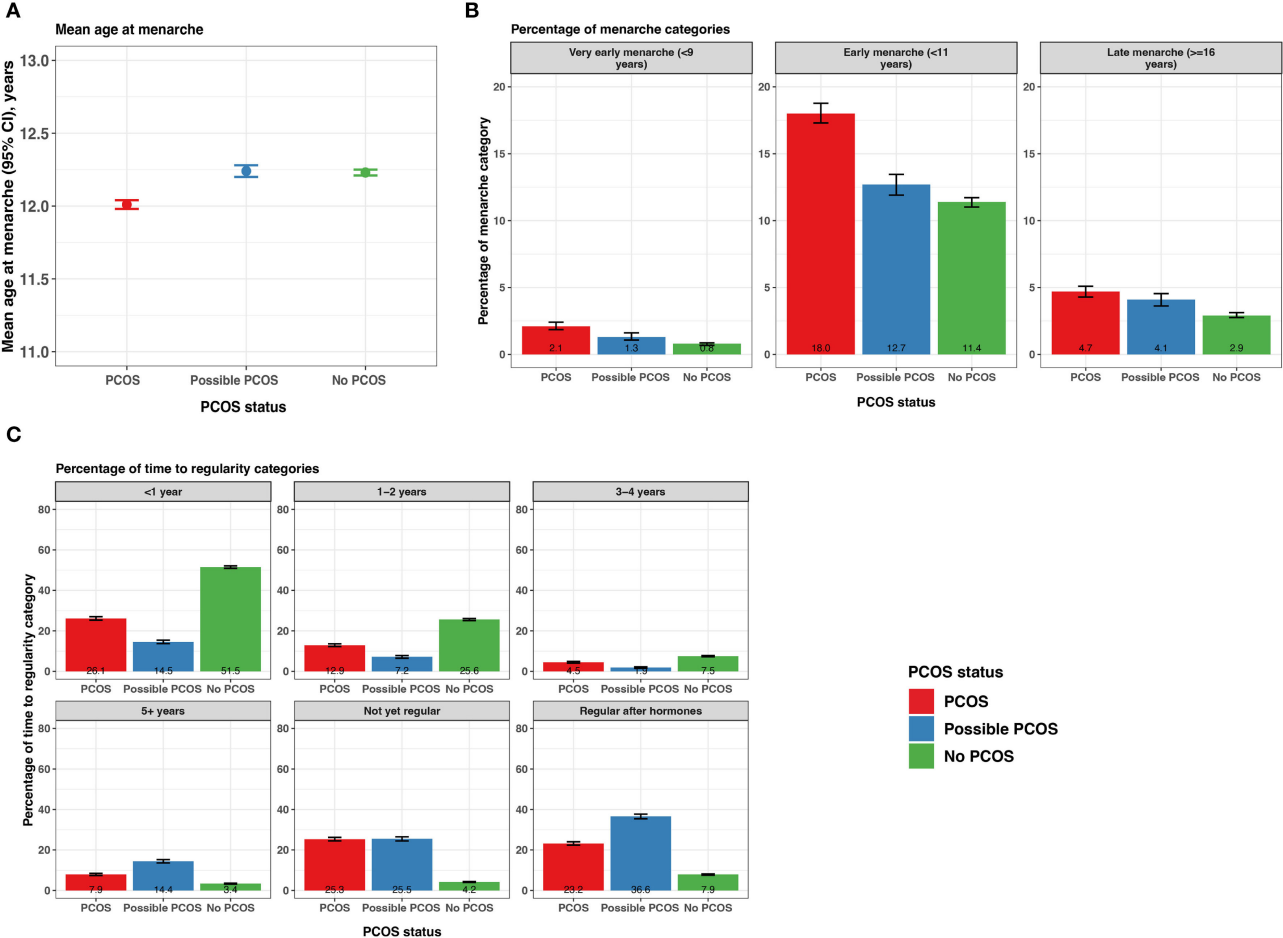
Menarche and time from menarche to cycle regularity among AWHS participants, stratified by PCOS status (PCOS, possible PCOS, no PCOS). **(A)** Mean age at menarche with 95% confidence intervals; **(B)** Percentage of participants with very early, early, and late menarche with 95% confidence intervals; **(C)** Percentage of self-reported time to regularity with 95% confidence intervals. Error bars represent 95% confidence intervals. AWHS, Apple Women’s Health Study; PCOS, polycystic ovary syndrome.

Age at menarche was lower for participants with PCOS (12.0 [95% CI 11.98 - 12.04] years) compared to those with possible PCOS and no PCOS (12.2 [95% CI 12.2 - 12.3] years and 12.2 [95% CI 12.2 - 12.3] years, respectively; **Figure 3**; **Supplementary Table S2**). Those with PCOS also reported very early menarche (2.1% [95% CI 1.9 - 2.4%]) and early menarche (18.0% [95% CI 17.3 - 18.8%]) significantly more than those with possible PCOS (very early: 1.3% [95% CI 1.1 - 1.6%], early: 12.7% [95% CI 11.9 - 13.5%]), followed by those without PCOS (very early: 0.8% [95% CI 0.7 - 0.9%], early: 11.4% [95% CI 11.0 - 11.7%]; **Figure 3**; **Supplementary Table S2**). The PCOS and possible PCOS groups reported late menarche (PCOS: 4.7% [95% CI 4.3 - 5.1%], possible PCOS: 4.1% [95% CI 3.6 - 4.5%]) more than the no PCOS group (2.9% [95% CI 2.8 - 3.1%]). Lastly, the majority of participants without PCOS achieved cycle regularity within 2 years of menarche (77.1% [95% CI: 76.6 - 77.6%]) compared to 39.0% [95% CI: 38.1 - 40.0%] of participants with PCOS and 21.7% [95% CI: 20.7, 22.6%] of participants with possible PCOS. Around half of the participants with PCOS (48.5%) and with possible PCOS (52.1%) were not yet regular at the time of assessment or had only achieved regularity after hormone use (**Figure 3**; **Supplementary Table S2**). Of note, the mean age ± standard deviation (SD) at PCOS diagnosis was 23.0 ± 7.01 years.

### Fertility and pregnancy complications

**Figure 4:**
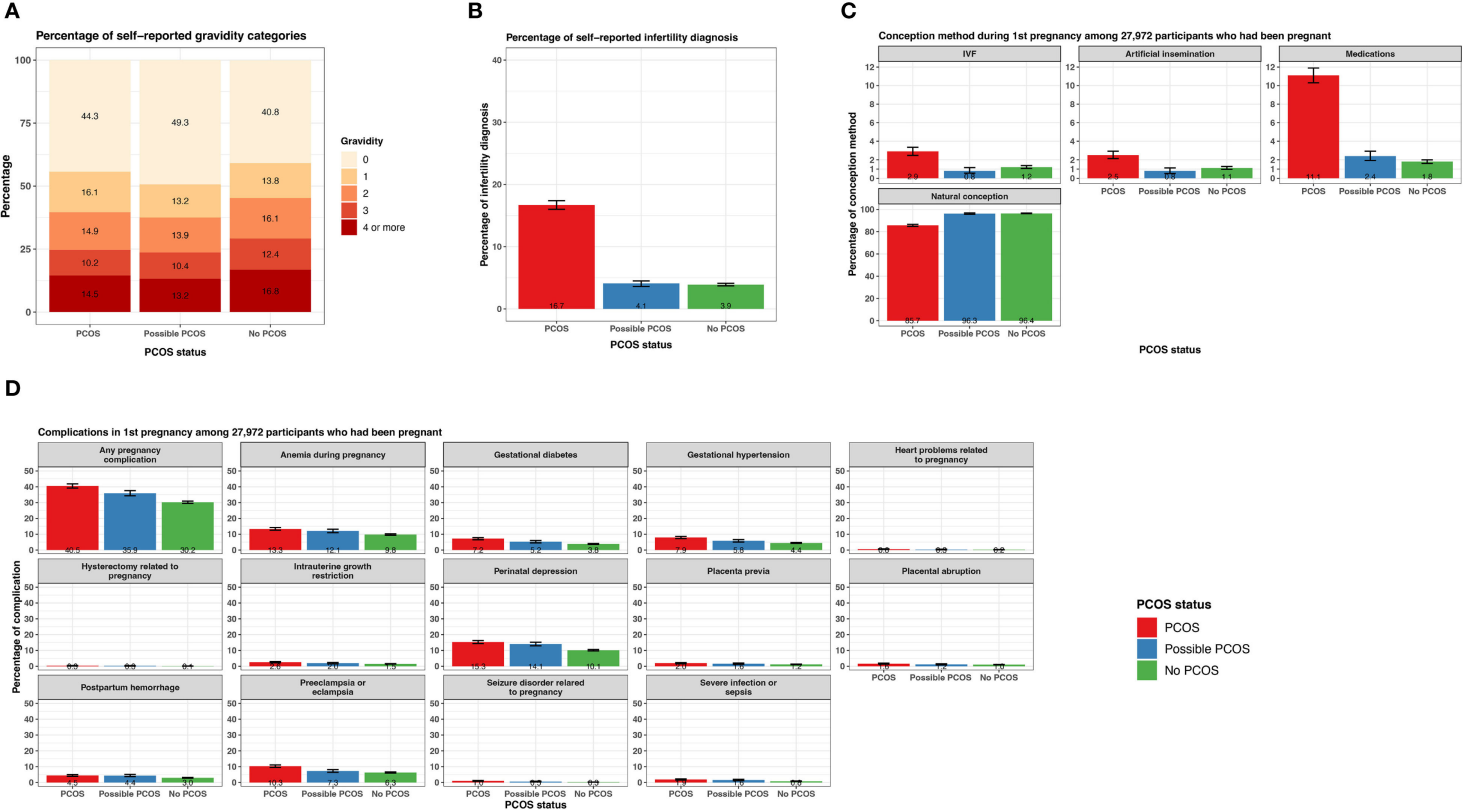
Fertility and pregnancy-related characteristics among AWHS participants, stratified by PCOS status (PCOS, possible PCOS, no PCOS). **(A)** Percentage of gravidity categories; **(B)** Percentage of infertility diagnosis with 95% confidence intervals; **(C)** Percentage of conception methods for first pregnancy with 95% confidence intervals, among a subset of participants who had been pregnant; **(D)** Percentage of complications relevant to first pregnancy with 95% confidence intervals, among a subset of participants who had been pregnant. Error bars represent 95% confidence intervals. AWHS, Apple Women’s Health Study; PCOS, polycystic ovary syndrome.

Those with PCOS reported an infertility diagnosis (16.7% [95% CI 16.0 - 17.4%]) over 4 times greater than those with possible PCOS (4.1% [95% CI 3.6 - 4.5%]) or without PCOS (3.9% [95% CI 3.7 - 4.1%]; **Figure 4**; **Supplementary Table S2**). The average age ± SD at the infertility diagnosis was youngest for those with PCOS (28.4 ± 8.0 years) and oldest for those without PCOS (32.9 ± 8.3 years; **Table 2**). Almost half of participants with possible PCOS (49.3% [95% CI: 48.1 - 50.4%]) reported never being pregnant, followed by participants with PCOS (44.3% [95% CI: 43.3 - 45.2%]), then participants without PCOS (40.8% [95% CI: 40.2 - 41.3%]). As gravidity increases, this trend inverses; participants without PCOS report a gravidity of 2, 3, and 4+ more (16.1% [95% CI: 15.7 - 16.5%], 12.4% [95% CI: 12.1 - 12.8], 16.8% [95% CI:16.4-17.3%], respectively) than those with PCOS (14.9% [95% CI: 14.2 - 15.6%], 10.2% [95% CI: 9.6 - 10.8%], 14.5% [95% CI: 13.9 - 15.2%], respectively) and possible PCOS (13.9% [95% CI: 13.1 - 14.7%], 10.4% [95% CI: 9.7 - 11.1%], 13.2% [95% CI: 12.4 - 14.0%], respectively; **Figure 4**; **Supplementary Table S2**).

**Table 2:** Age at diagnosis for health conditions by PCOS status, among subsets of Apple Women’s Health Study participants who provided data on age at diagnosis.

Health conditions	N who provided age at diagnosis information / N with condition	Mean ± SD of age at diagnosis, years			P-value^a^
		PCOS	Possible PCOS	No PCOS	
Infertility	669 / 3380	28.4 ± 8.0	31.1 ± 9.3	32.9 ± 8.3	<.0001
Prediabetes	2223 / 5514	27.7 ± 9.9	36.4 ± 13.1	37.1 ± 12.1	<.0001
Type 2 diabetes	748 / 1883	32.2 ± 10.3	37.3 ± 12.4	38.2 ± 11.5	<.0001
Hypertension	2721 / 6944	30.5 ± 9.3	35.7 ± 13.3	36.6 ± 11.6	<.0001
High cholesterol	2996 / 7566	30.7 ± 10.3	36.4 ± 13.3	35.5 ± 12.8	<.001
Arrhythmia	914 / 2279	28.8 ± 13.3	28.5 ± 14.3	31.1 ± 16.5	.10
Congestive heart failure	136 / 306	33.6 ± 13.4	35.8 ± 19.9	40.0 ± 15.2	.06
Coronary artery disease	80 / 210	37.5 ± 11.2	47.9 ± 13.9	54.0 ± 11.1	<.0001
Heart attack	143 / 300	36.9 ± 11.5	41.3 ± 13.7	41.4 ± 13.4	.15
Stroke	143 / 383	35.3 ± 9.6	33.5 ± 12.7	34.9 ± 12.4	.75
Transient ischemic attack (mini-stroke)	232 / 553	31.6 ± 10.5	33.7 ± 15.1	36.1 ± 12.9	.11
Endometrial cancer	59 / 143	33.8 ± 12.0	32.3 ± 17.7	41.9 ± 14.5	.05
Endometrial hyperplasia	158 / 378	30.1 ± 9.1	29.4 ± 9.2	31.4 ± 11.4	.91
Anorexia	856 / 2147	17.3 ± 6.9	16.7 ± 5.3	17.4 ± 6.2	.33
Anxiety	10503 / 26067	21.3 ± 8.9	21.9 ± 9.7	23.3 ± 9.9	<.0001
Attention deficit & hyperactivity disorder (ADHD)	5356 / 12190	21.8 ± 11.2	22.2 ± 11.8	23.6 ± 12.1	<.0001
Bipolar disorder	2084 / 4855	22.1 ± 8.3	22.5 ± 9.3	23.6 ± 9.0	.006
Bulimia	498 / 1311	16.8 ± 4.9	18.3 ± 5.6	18.9 ± 7.1	.01
Depression	10502 / 26524	20.1 ± 8.1	21.1 ± 9.2	22.3 ± 9.4	<.0001
Panic disorder	2339 / 5889	21.1 ± 9.7	21.7 ± 9.7	23.1 ± 9.6	<.0001

PCOS, polycystic ovary syndrome.

a P-values from Kruskal-Wallis test.

The following pregnancy-related data pertain to outcomes during a participant’s first pregnancy. Of the 27,972 participants that reported a gravidity ≥1 and shared their pregnancy history information, those without PCOS conceived naturally at a high frequency (96.4% [95% CI 96.2 - 96.8%]) similar to those with possible PCOS (96.3% [95% CI 95.7 - 96.9%]). Those with PCOS conceived naturally at a lower frequency (85.7% [95% CI 84.8 - 86.9%]) and conceived with methods such as IVF (2.9% [95% CI 2.5 - 3.3%]), artificial insemination (2.5% [95% CI 2.1 - 2.9%]), and medications (11.1% [95% CI 10.3 - 11.9%]) more than those with possible PCOS (IVF: 0.8% [95% CI 0.6 - 1.2%], artificial insemination: 0.8% [95% CI 0.5 - 1.1%], medications: 2.4% [95% CI 1.9 - 2.9%]) and without PCOS (IVF: 1.2% [95% CI 1.1 - 1.4%], artificial insemination: 1.1% [95% CI 1.0 - 1.3%], medications: 1.8% [95% CI 1.6 - 2.0%]; **Figure 4**; **Supplementary Table S2**).

There were differences in prevalence for all pregnancy conditions when comparing the three PCOS status groups (**Supplementary Table S2**). Of note, any pregnancy complications, gestational diabetes, and gestational hypertension were experienced at the highest frequency for participants with PCOS (any: 40.5% [95% CI 39.2 - 41.8%], gestational diabetes: 7.2% [95% CI 6.5 - 7.9%], gestational hypertension: 7.9% [95% CI 7.2 - 8.6%]), followed by participants with possible PCOS (any: 35.9% [95% CI 34.3 - 37.5%], gestational diabetes: 5.2% [95% CI 4.5 - 6.0%], gestational hypertension: 5.8% [95% CI 5.0 - 6.6%]), then participants without PCOS (any: 30.2% [95% CI 29.5 - 30.9%], gestational diabetes: 3.8% [95% CI 3.5 - 4.1%], gestational hypertension: 4.4% [95% CI 4.1 - 4.7%]; **Figure 4**; **Supplementary Table S2**). Preeclampsia was experienced more frequently among participants with PCOS (10.3% [95% CI 9.6 - 11.1%]) than participants with possible PCOS (7.3% [95% CI 6.5 - 8.2%]) or without PCOS (6.3 [95% CI 6.0 - 6.7%]; **Figure 4**; **Supplementary Table S2**). Lastly, anemia during pregnancy and perinatal depression were experienced at similar frequencies for participants with PCOS and possible PCOS (PCOS: anemia 13.3% [95% CI 12.5 - 14.2%], perinatal depression: 15.2 [95% CI 14.4 - 16.2%]; possible PCOS: anemia 12.1% [95% CI 11.0 - 13.1%], perinatal depression: 14.1% [95% CI 12.9 - 15.2%]) which was more than participants without PCOS (anemia 9.8% [95% CI 9.4 - 10.2%], perinatal depression: 10.1% [95% CI 9.7 - 10.6%]; **Figure 4**; **Supplementary Table S2**).

#### Chronic diseases

**Figure 5:**
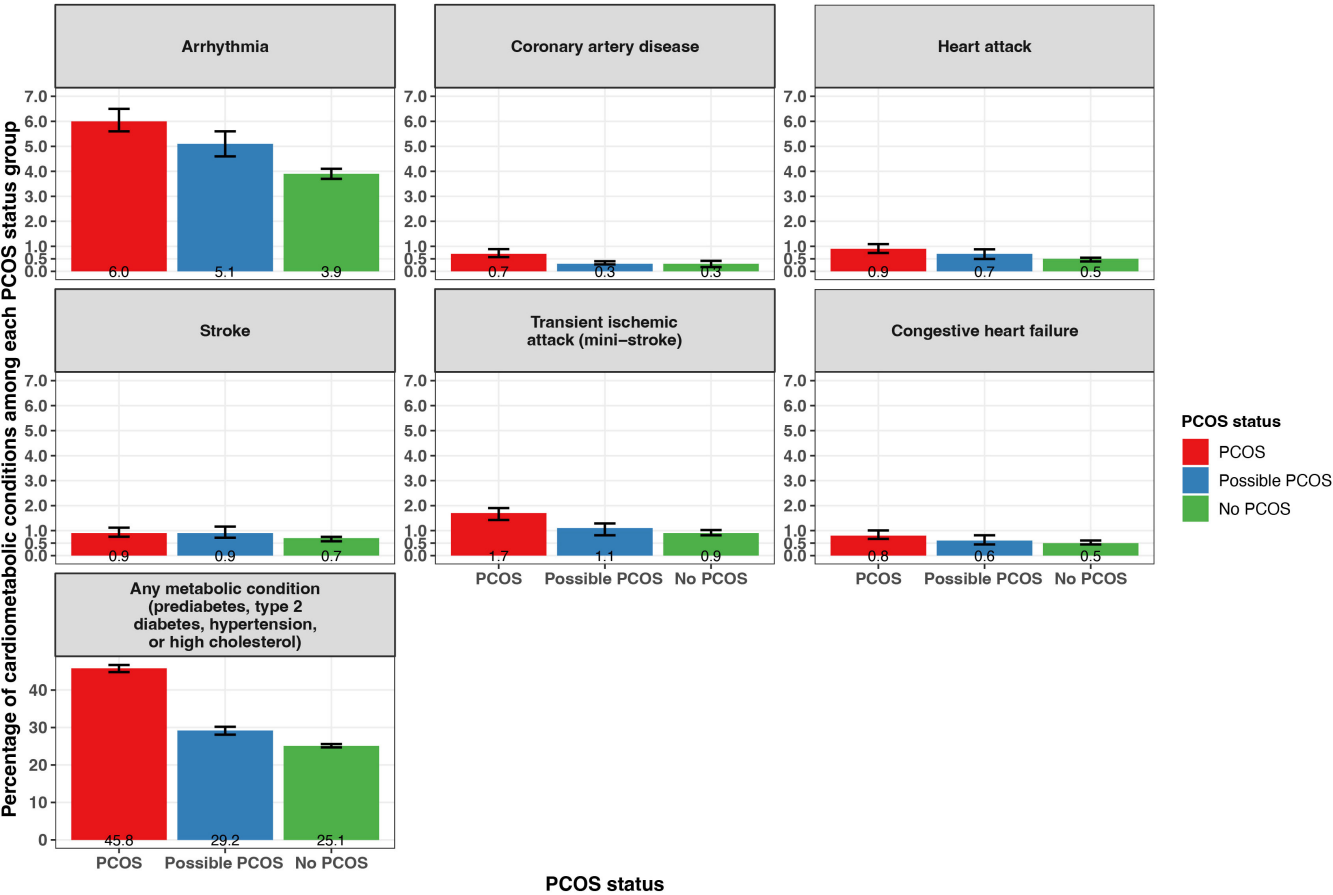
Percentage of self-reported metabolic and cardiometabolic conditions with 95% confidence intervals among AWHS participants, stratified by PCOS status (PCOS, possible PCOS, no PCOS). Error bars represent 95% confidence intervals. AWHS, Apple Women’s Health Study; PCOS, polycystic ovary syndrome.

Reported prevalence of all cardiometabolic and metabolic conditions differed when comparing PCOS status groups (**Figure 5**; **Supplementary Table S2**). Notably, any metabolic condition (prediabetes, type 2 diabetes [T2DM], hypertension, or high cholesterol) was reported at the highest frequency for participants with PCOS (45.8% [95% CI 44.8 - 46.7%]), followed by those with possible PCOS (29.2% [95% CI 28.1 - 30.2%]), then participants without PCOS (25.1% [95% CI 24.7 - 25.6%]). Arrhythmia was the cardiometabolic condition reported at the highest frequency along with the largest magnitude difference across the three groups (PCOS: 6.0% [95% CI 5.6 - 6.5%], possible PCOS: 5.1% [95% CI 4.6 - 5.6%], no PCOS: 3.9% [95% CI 3.7 - 4.1%]). Prediabetes, T2DM, hypertension, and coronary artery disease were all diagnosed earlier in life for individuals with PCOS, followed by those with possible PCOS, and later in life for individuals without PCOS (**Figure 5**; **Table 2**). Notably, prediabetes and coronary artery disease were diagnosed 9.4 and 16.5 years earlier, respectively, for the PCOS group compared to the no PCOS group. High cholesterol was diagnosed latest in life for those with possible PCOS. There was no significant difference in age at diagnosis for arrhythmia, congestive heart failure, heart attack, stroke, or transient ischemic attack across PCOS status (**Table 2**).

**Figure 6:**
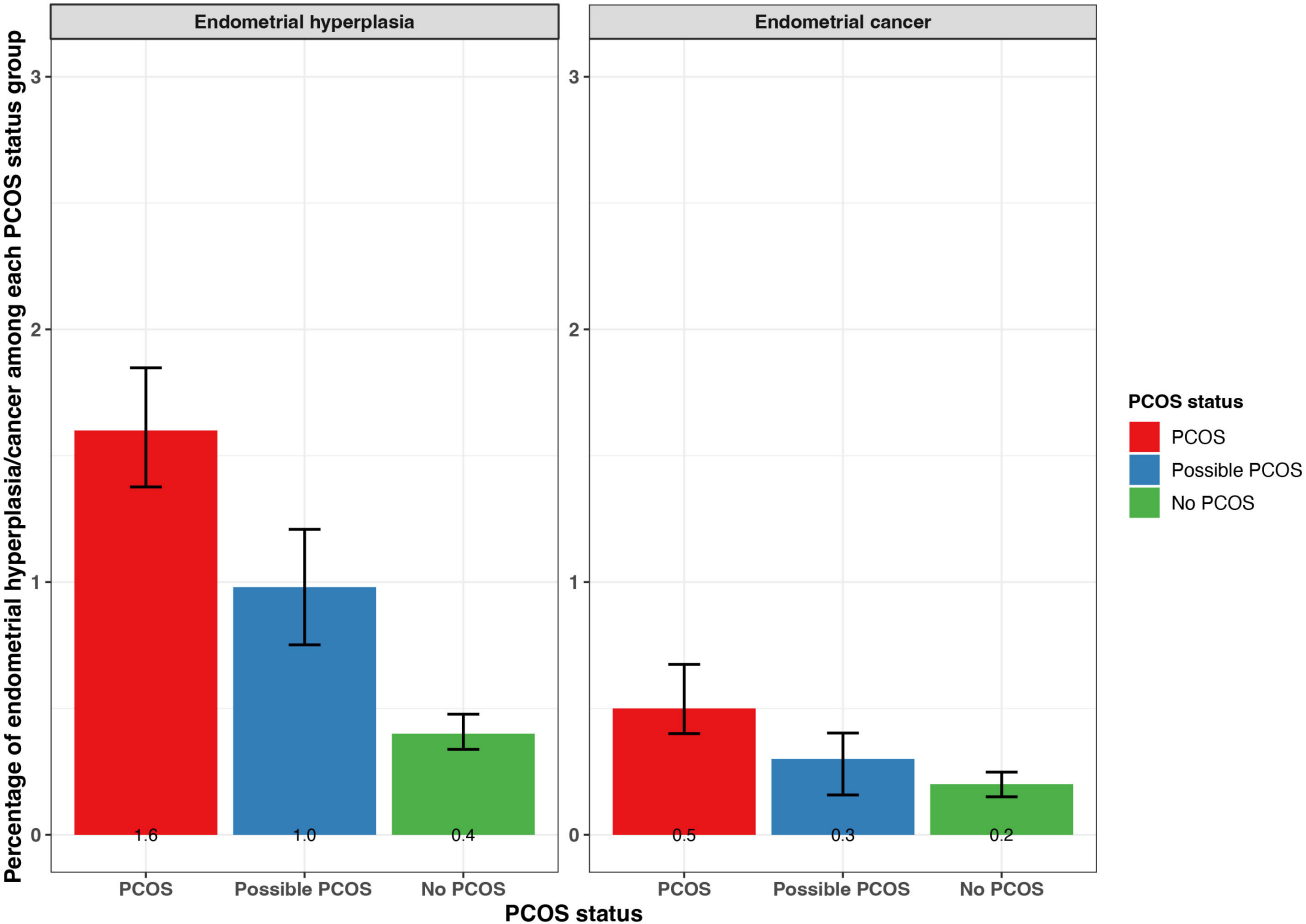
Percentage of self-reported endometrial cancer and endometrial hyperplasia with 95% confidence intervals among AWHS participants, stratified by PCOS status (PCOS, possible PCOS, no PCOS). Error bars represent 95% confidence intervals. AWHS, Apple Women’s Health Study; PCOS, polycystic ovary syndrome.

Additionally, endometrial cancer was reported most by participants with PCOS (0.5% [95% CI 0.4 - 0.7%]), followed by those with possible PCOS (0.3% [95% CI 0.2 - 0.4%]), then those without PCOS (0.2% [95% CI 0.15 - 0.25%]; **Figure 6**). Endometrial hyperplasia was reported at similar frequencies for participants with PCOS (1.6% [95% CI 1.4 - 1.8%]) and possible PCOS (1.0 [95% CI 0.8 - 1.2]) when compared to no PCOS participants (0.4% [95% CI 0.3 - 0.5%]; **Figure 6**). Endometrial cancer was diagnosed earliest among those with possible PCOS (32.3 years), then for those with PCOS (33.8 years), and 8.1 years later for those without PCOS (41.9 years; **Table 2**).

#### Mental health

**Figure 7:**
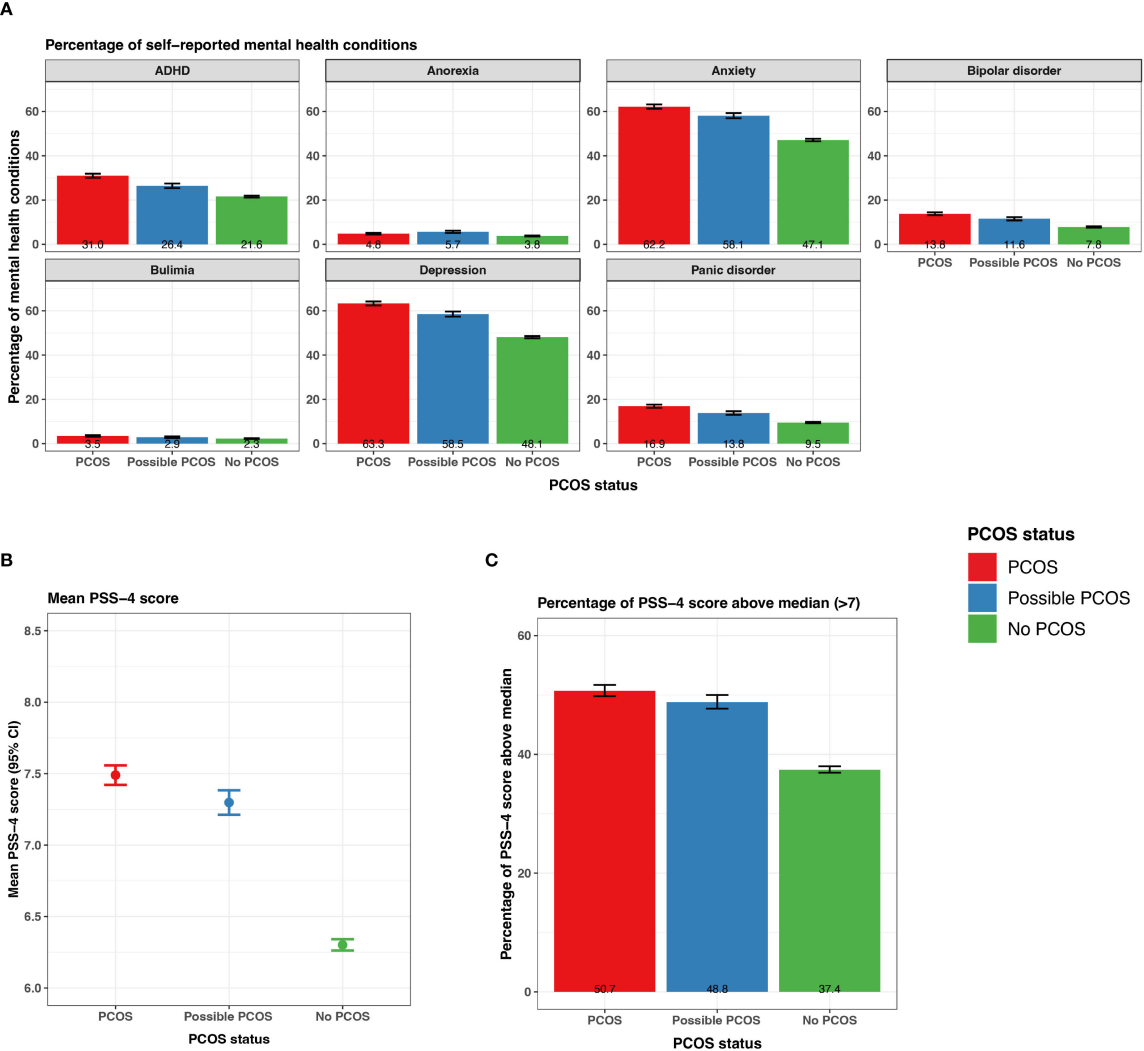
Mental health characteristics among AWHS participants, stratified by PCOS status (PCOS, possible PCOS, no PCOS). **(A)** Percentage of self-reported mental health conditions with 95% confidence intervals; **(B)** Mean PSS - 4 score with 95% confidence intervals; **(C)** Percentage of PSS - 4 scores above the study population median (>7) with 95% confidence intervals. Error bars represent 95% confidence intervals. AWHS, Apple Women’s Health Study; PCOS, polycystic ovary syndrome; PSS: perceived stress scale.

All mental health conditions with the exception of eating disorders (anorexia, bulimia) were experienced at the highest frequency for those with PCOS, followed by those with possible PCOS, and those without PCOS had the lowest incidence frequencies (**Figure 7**; **Supplementary Table S2**). Anorexia and bulimia were reported at similar frequencies for both the PCOS and possible PCOS groups (anorexia: PCOS 4.8% [95% CI 4.4 - 5.2%], possible PCOS 5.7% [95% CI 5.1 - 6.2%]; bulimia: PCOS 3.5% [95% CI 3.1 - 3.8%], possible PCOS 2.9% [95% CI 2.5 - 3.3%]) compared to the no PCOS group (anorexia: 3.8% [95% CI 3.6 - 4.0%], bulimia: 2.3% [95% CI 2.1 - 2.4%]; **Figure 7**; **Supplementary Table S2**). Additionally, all mental health conditions with the exception of anorexia were diagnosed at different ages when comparing by PCOS status. On average, mental health conditions were diagnosed 2 years earlier in participants with PCOS compared to those without (**Table 2**).

#### BMI

The median BMI was highest in the PCOS group (32.4 kg/m^2^), followed by the possible PCOS group (27.5 kg/m^2^), then the no PCOS group (26.6 kg/m^2^; **Table 1**). Additionally, participants in the PCOS group had a BMI that fell into the obesity 1, 2, and 3 categories at the highest proportion (obesity 1: 20.4%, 2: 16.8%, 3: 21.7%), followed by those with possible PCOS (obesity 1: 17.0%, 2: 10.7%, 3: 9.5%), then those without PCOS (obesity 1: 16.6%, 2: 9.2%, 3: 7.4%; **Table 1**). Those with possible PCOS had the highest proportion of participants underweight (PCOS: 1.5%, possible PCOS: 3.2%, no PCOS: 2.6%). Lastly, those without PCOS reported the highest proportion of participants in the healthy (36.1%) and overweight (26.4%) categories, although the possible PCOS group has similar frequencies (healthy: 32.6%, overweight: 25.0%) whereas fewer people with PCOS were healthy or overweight (healthy: 17.8%, overweight: 19.7%; **Table 1**).

The BMI sensitivity analysis revealed that when using the categories the WHO recommends for Asians rather than the CDC BMI cutoff thresholds for the general public, the percentage of participants in the underweight, healthy, overweight, and obesity categories at baseline are similar by PCOS status (**Table 1**; **Supplementary Tables S2**, **S3**).

**Figure 8:**
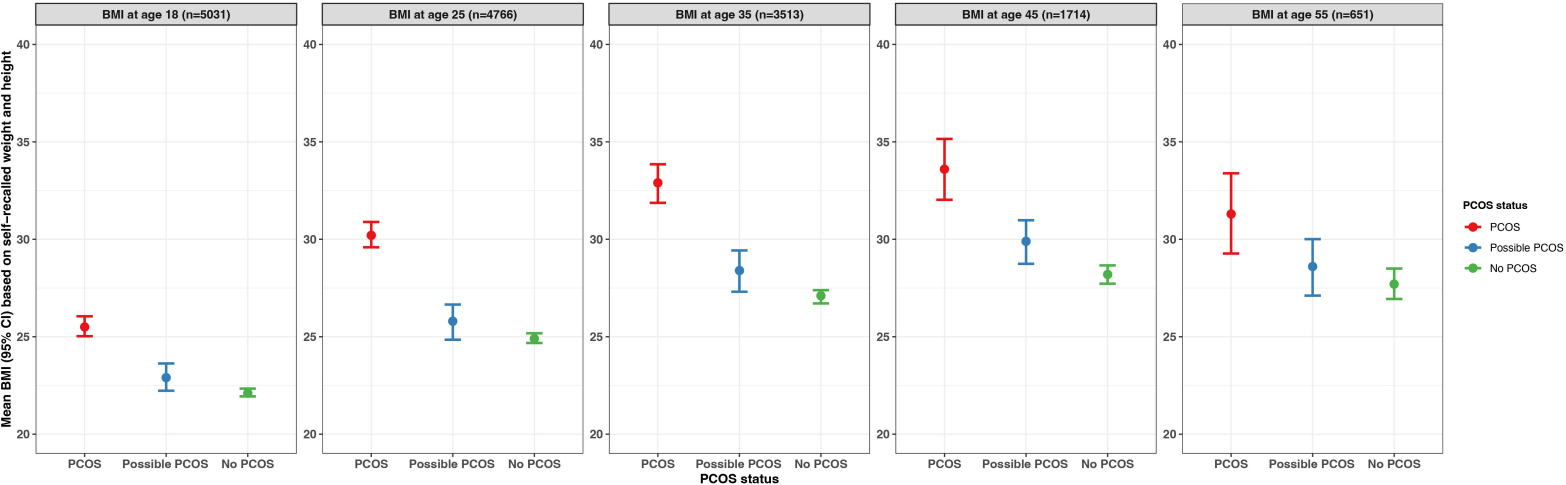
Average BMI by age (18, 25, 35, 45, 55 years) with 95% confidence intervals among subsets of AWHS participants who provided information on recalled BMI, stratified by PCOS status (PCOS, possible PCOS, no PCOS). Error bars represent 95% confidence intervals. AWHS, Apple Women’s Health Study; PCOS, polycystic ovary syndrome; BMI: body mass index.

When BMI was assessed by age (18, 25, 35, 45, 55 years) among the subset of participants who recalled their BMI history, for all groups, the average BMI increased as age increased until the last assessment (age 55 years) when the average BMI decreased. For each age period assessed with the exception of age strata 55 years, the average BMI was highest in the PCOS group and similar across the possible PCOS and no PCOS groups (**Figure 8**; **Supplementary Table S2**). The BMI differences across groups were smallest during the age 18 years evaluation (PCOS: 25.5 kg/m^2^; possible PCOS: 22.9 kg/m^2^; no PCOS: 22.1 kg/m^2^) and largest differences at the age 35-year evaluation (PCOS: 32.9 kg/m^2^; possible PCOS: 28.4 kg/m^2^; no PCOS: 27.1 kg/m^2^). After the 18-year age evaluation, at all age timepoints the average BMI for the PCOS group fell into the obesity 1 category. The average BMI for the possible PCOS group fell into the overweight category, and participants without PCOS had an average BMI that was considered overweight for the last three age assessments (**Supplementary Table S2**).

### Lifestyle behaviors

#### Nutrition

**Figure 9:**
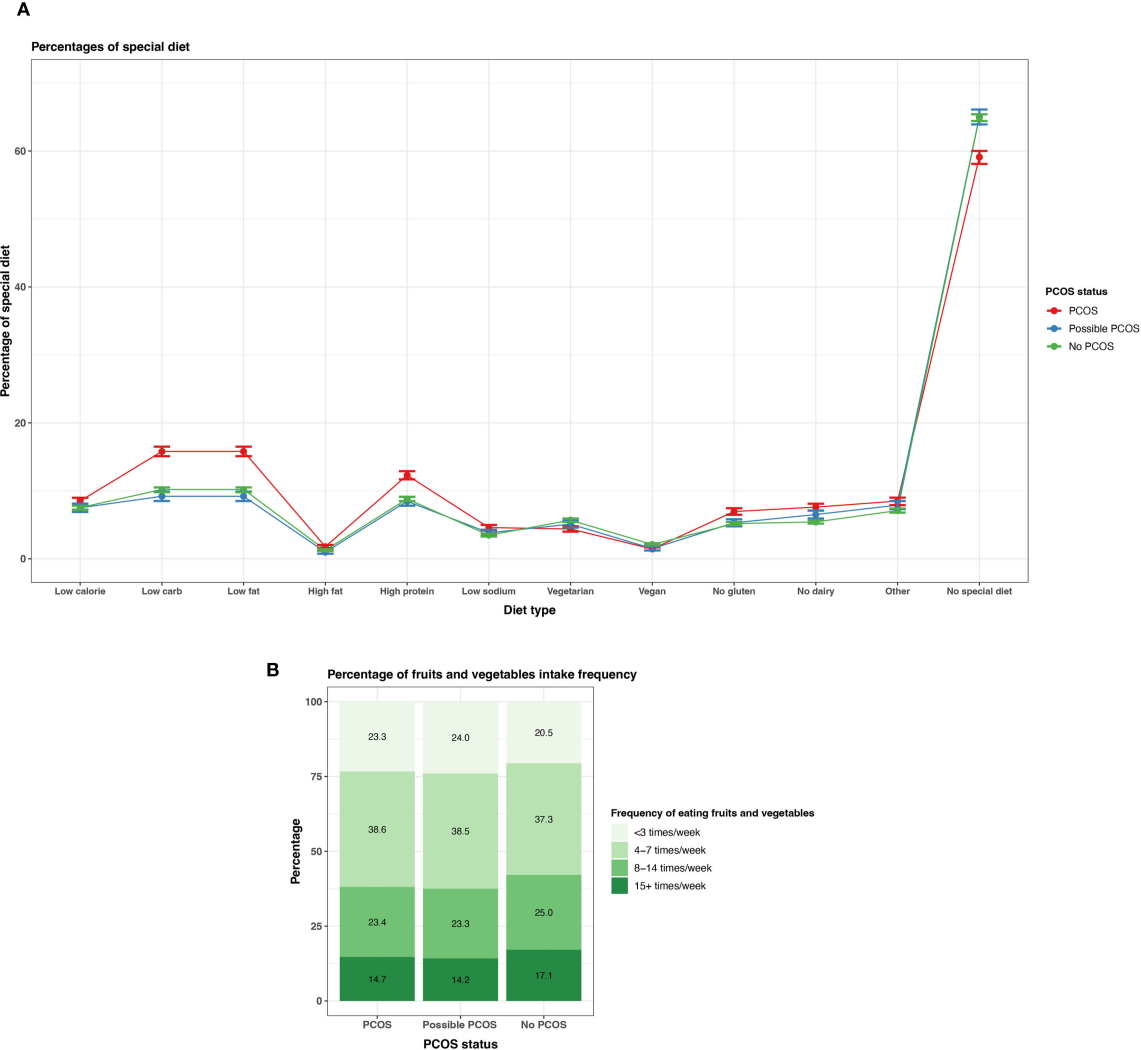
Dietary patterns among AWHS participants, stratified by PCOS status (PCOS, possible PCOS, no PCOS). **(A)** Percentages of self-reported special diets with 95% confidence intervals; **(B)** Percentages of typical weekly fruit and vegetable intake Error bars represent 95% confidence intervals. AWHS, Apple Women’s Health Study; PCOS, polycystic ovary syndrome.

Participants with PCOS reported following at least one special diet (40.9% [95% CI 40.0 - 41.9%]) more so than those with possible PCOS (35.1% [95% CI 34.6 - 35.6%]) or without PCOS (35.0% [95% CI 33.9 - 36.1%]). There is a suggested trend that participants with PCOS follow low carbohydrates, low fat, high protein, gluten free, and dairy free diets (**Figure 9**, **Supplementary Table S4**). There is also a trend of participants with PCOS or possible PCOS reporting fruit and vegetable consumption less than participants without PCOS (<3 times/week PCOS: 23.3% [22.5 - 24.1%], possible PCOS: 24.0% [95% CI 23.0 - 25.0%], no PCOS: 20.5% [95% CI 20.0 - 20.9%]; **Figure 9**; **Supplementary Table S4**).

#### Physical activity

**Figure 10:**
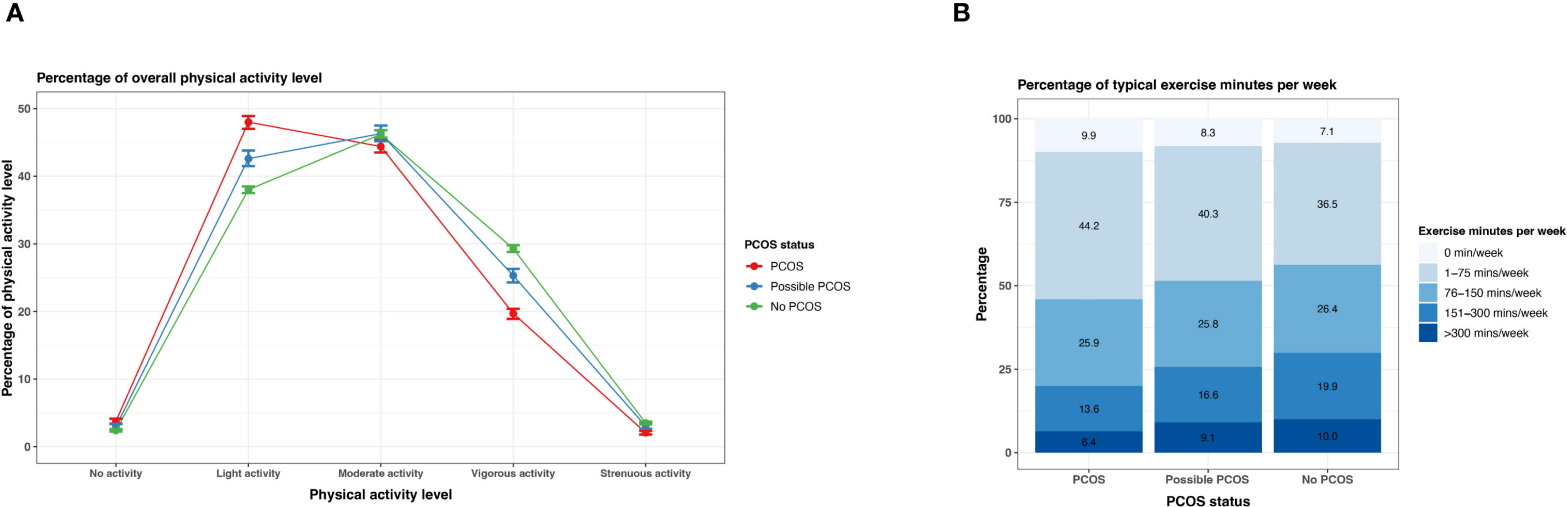
Physical activity among AWHS participants, stratified by PCOS status (PCOS, possible PCOS, no PCOS). **(A)** Percentages of self-reported overall physical activity levels with 95% confidence intervals; **(B)** Percentages of weekly exercise minutes. Error bars represent 95% confidence intervals. AWHS, Apple Women’s Health Study; PCOS, polycystic ovary syndrome.

Reporting none or light physical activity was most prevalent in the PCOS group (none: 3.8% [95% CI 3.4 - 4.1%], light 48.0% [95% CI 47.0 - 48.9%]), followed by the possible PCOS group (none: 3.0% [95% CI 2.6 - 3.4%], light 42.6% [95% CI 41.5 - 43.8%]), then the no PCOS group (none: 2.4% [95% CI 2.3 - 2.6%], light 38.0% [95% CI 37.5 - 38.5%]). This trend inversed for vigorous and strenuous exercise as those without PCOS show a higher reporting prevalence (**Figure 10**; **Supplementary Table S4**). The same trend is prevalent for reported weekly exercise minutes; reporting ≤150 minutes was more prevalent for the PCOS group (35.8%) followed by the possible PCOS group (34.1%), but the pattern inverses for reported weekly exercise minutes >150 minutes (**Figure 10**; **Supplementary Table S4**).

#### Stress

Those with PCOS reported a mean PSS-4 score of 7.5 (95% CI 7.4 - 7.6) which was slightly higher than those with possible PCOS (7.3 [95% CI 7.2 - 7.4]), while those without PCOS reported a mean PSS score of 6.3 (95% CI 6.26 - 6.34; **Figure 7**). Around half of participants with PCOS (50.7% [95% CI 49.8 - 51.7%]) and participants with possible PCOS (48.8% [47.7 - 50.0%]) reported a PSS-4 higher than the study median PSS score (7). Fewer participants without PCOS (37.4% [36.9 - 38.0%]) reported a PSS-4 score greater than the study population median (**Figure 7**; **Supplementary Table S4**).

#### Substance use

**Figure 11:**
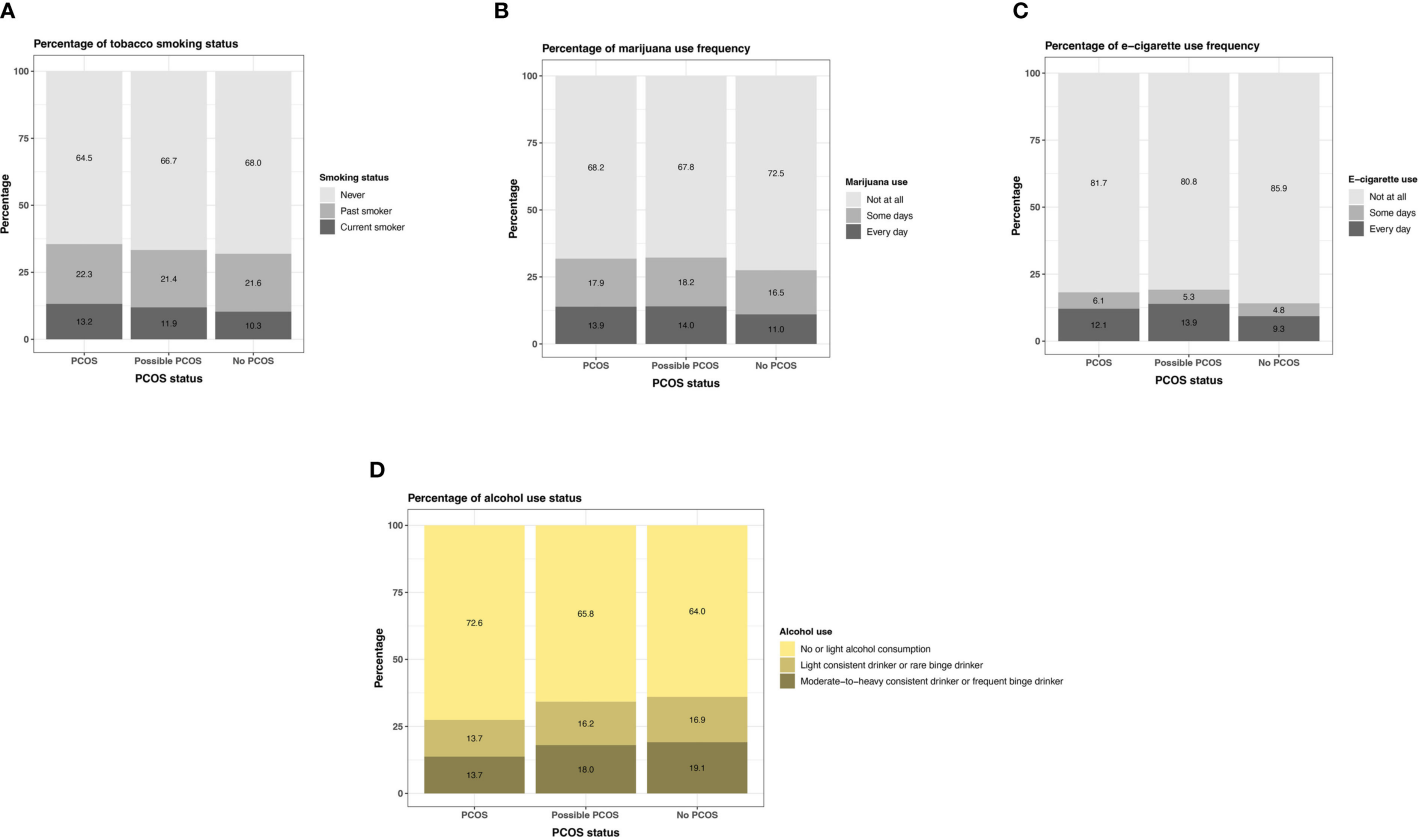
Tobacco, marijuana, e-cigarette, and alcohol use among AWHS participants, stratified by PCOS status (PCOS, possible PCOS, no PCOS). **(A)** Percentages of self-reported tobacco use; **(B)** Percentages of self-reported marijuana use; **(C)** Percentages of self-reported e-cigarette use; **(D)** Percentages of self-reported alcohol use. AWHS, Apple Women’s Health Study; PCOS, polycystic ovary syndrome.

Participants with PCOS and possible PCOS reported currently smoking tobacco and marijuana at similar greater proportions (PCOS: tobacco 13.2% [95% CI: 12.6 - 13.9%], marijuana 13.9% [95% CI: 13.3 - 14.6%]; possible PCOS: tobacco 11.9% [95% CI: 11.2 - 12.7%], marijuana 14.0% [95% CI: 13.2 - 14.8%]), than those without PCOS (tobacco 10.3% [95% CI: 10.0 - 10.7%], marijuana 11.0% [95% CI: 10.7 - 11.4%]; **Figure 11**). Daily e-cigarette use was highest for those with possible PCOS (13.9% [95% CI: 13.1 - 14.7%]), followed by those with PCOS (12.1% [95% CI: 11.5 - 12.8%]), then participants without PCOS (9.3% [95% CI: 9.0 - 9.6%]; **Figure 11**; **Supplementary Table S4**).

Participants with PCOS reported not drinking (13.4% [95% CI: 12.7 - 14.1%]) more than those with possible PCOS (12.2% [95% CI: 11.4 - 13.0%]) and without PCOS (11.9% [95% CI: 11.5 - 12.3%]). The number of drinks on a typical night remain similar across groups (**Supplementary Table S4**). When comparing the combined alcohol variable, those with PCOS follow a no or light alcohol consumption trend (72.6% [95% CI: 71.7 - 73.6%]) more than the other groups (possible PCOS: 65.8% [95% CI: 64.6 - 67.0%], without PCOS: 64.0% [95% CI: 63.4 - 64.6%]) and those without PCOS are most frequently in the moderate-to-heavy consistent category (PCOS: 13.7% [95% CI: 12.9 - 14.4%], possible PCOS: 18.0% [95% CI: 17.0 - 19.0%], without PCOS: 19.1% [95% CI: 18.6 - 19.6%]; **Figure 11**; **Supplementary Table S4**).

#### Sleep

**Figure 12:**
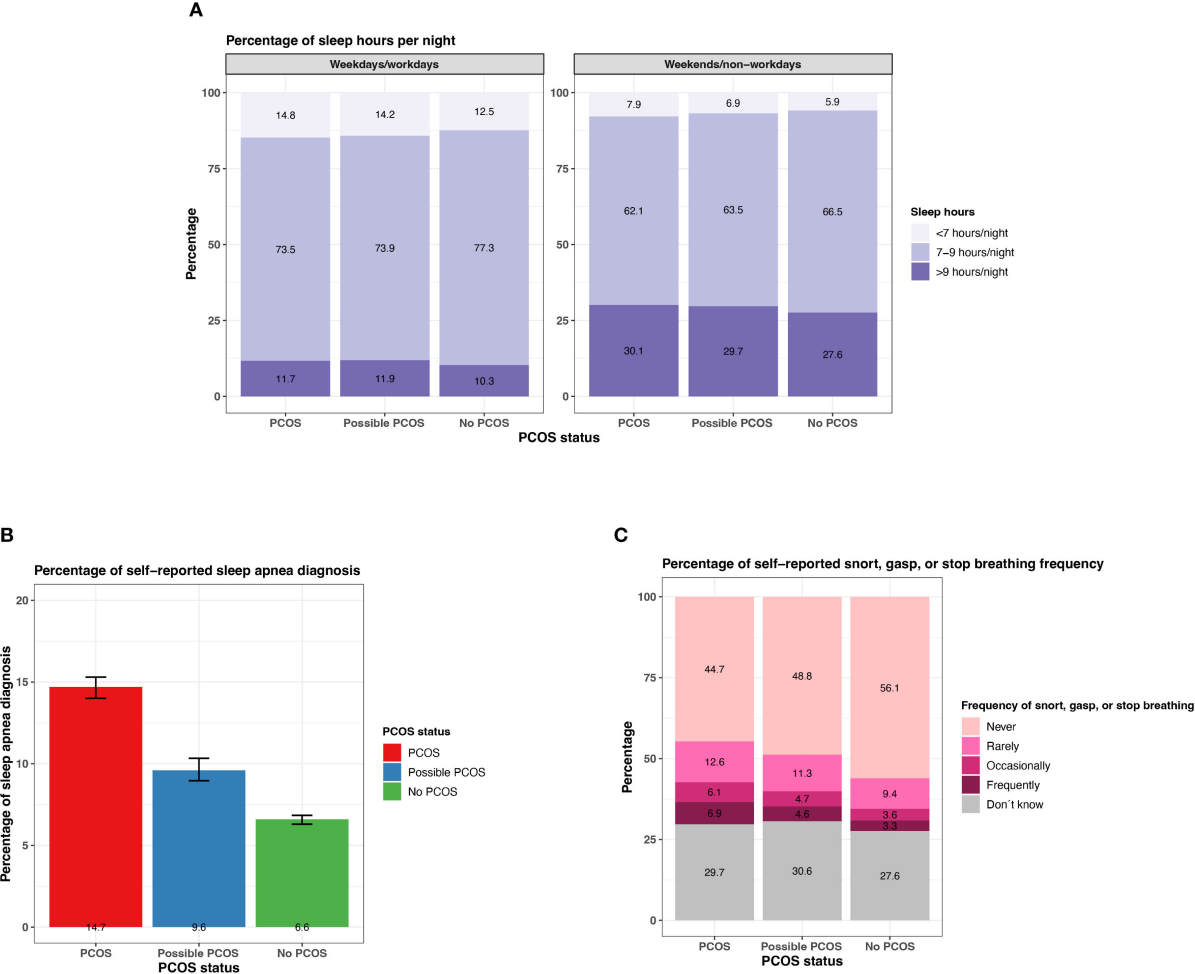
Sleep characteristics among AWHS Study participants, stratified by PCOS status (PCOS, possible PCOS, no PCOS). **(A)** Percentages of self-reported sleep hours on weekdays/workdays and on weekends/non-workdays; **(B)** Percentages of self-reported sleep apnea diagnoses with 95% confidence intervals; **(C)** Percentages of self-reported snorting, gasping, and stopping breathing frequency. Error bars represent 95% confidence intervals. AWHS, Apple Women’s Health Study; PCOS, polycystic ovary syndrome.

Participants with PCOS and possible PCOS report <7 hours or >9 hours more than those without PCOS on typical weekdays/workdays (<7 hours: PCOS: 14.8% [95% CI: 14.2 - 15.5%], possible PCOS: 14.2% [95% CI: 13.4 - 15.0%], no PCOS: 12.5% [95% CI: 12.1 - 12.8%]; >9 hours: PCOS: 11.7% [95% CI: 11.1 - 12.3%], possible PCOS: 11.9% [95% CI: 11.2 - 12.7%], no PCOS: 10.3% [95% CI: 9.9 - 10.6%]; **Figure 12**; **Supplementary Table S4**). Similar differences are found for sleep hours on weekends/non-workdays. There is also a higher proportion of participants with PCOS reporting a sleep apnea diagnosis (14.7% [95% CI: 14.0 - 15.3%]), followed by those with possible PCOS (9.6% [95% CI: 9.0 - 10.3%]), then those without PCOS (6.6% [95% CI:6.3-6.8%]; **Figure 12**). Participants with PCOS also reported breathing disturbances (snorting, gasping, or stopping breathing while asleep) more frequently (occasionally/frequently: 13.0% [95% CI: 12.4 - 13.6%]), followed by those with possible PCOS (occasionally/frequently: 9.3% [95% CI: 8.6 - 9.9%]), then those without PCOS (occasionally/frequently: 6.9% [95% CI: 6.6 - 7.2%]; **Figure 12**; **Supplementary Table S4**).

#### Secondary analysis: possible PCOS

The logistic regression model highlighted multiple characteristics that were associated with the likelihood of not reporting having received a PCOS diagnosis despite having the relevant symptoms to meet the diagnostic criteria (i.e. possible PCOS). Being younger or older than adulthood/reproductive stage at baseline was associated with a higher likelihood of possible PCOS. For example, age 18 – 19 years at baseline was associated with 2.47 times higher odds of not reporting having received a PCOS diagnosis (OR 2.47 [95% CI 2.02 - 3.01]) and being 50 or older was associated with 3.65 higher odds (OR 3.65 [95% CI 3.24 - 4.12]; **Table 3**).

**Table 3:** Comparing those who had possible PCOS but did not report having received a diagnosis to those who reported a PCOS diagnosis, fitting all baseline characteristics in a logistic regression with possible PCOS as the outcome (i.e., a possibly missed PCOS diagnosis).

Baseline predictor variables	Odds ratio of a possibly missed PCOS diagnosis	95% CI of odds ratio	P-value
Age, years			
18-19	2.47	2.02 – 3.01	<.0001
20-29	1.49	1.38 – 1.62	<.0001
30-39 (REF)	REF	REF	REF
40-49	1.20	1.09 – 1.32	<.001
≥50	3.65	3.24 – 4.12	<.0001
Race and ethnicity			
Non-Hispanic white (REF)	REF	REF	REF
Non-Hispanic Black	1.03	0.86 – 1.22	.74
Asian	0.40	0.32 – 0.50	<.0001
Hispanic	0.79	0.69 – 0.90	<.001
Multiple/other races	0.88	0.80 – 0.97	.01
Education level			
High school or below	1.24	1.10 – 1.40	<.001
Some college or tech school	1.05	0.95 – 1.15	.37
College degree	1.07	0.97 – 1.18	.18
Graduate degree	REF	REF	REF
Subjective socioeconomic status (SES)			
Low (0-3)	1.14	1.04 – 1.26	<.01
Medium (4-5)	1.02	0.94 – 1.11	.61
High (6-9)	REF	REF	REF
Body mass index, kg/m^2^			
Underweight (<18.5)	1.58	1.26 – 1.98	<.0001
Healthy weight (18.5-24.9)	1.44	1.31 – 1.58	<.0001
Overweight (25.0-29.9)	REF	REF	REF
Obesity 1 (30.0-34.9)	0.65	0.59 – 0.71	<.0001
Obesity 2 (35.0-39.9)	0.49	0.44 – 0.55	<.0001
Obesity 3 (≥40)	0.34	0.31 – 0.38	<.0001
Reported family history of PCOS, yes vs. no	0.23	0.21 – 0.26	<.0001

PCOS, polycystic ovary syndrome.

Additionally, individuals of low educational obtainment (high school or below: OR 1.24 [95% CI 1.10 - 1.40]), of low subjective SES (OR 1.14 [95% CI 1.04 - 1.26]), and being underweight (OR 1.58 [95% CI 1.26 - 1.98]) or healthy weight (OR 1.44 [95% CI 1.31 - 1.58]) at baseline increased the odds of not reporting having received a PCOS diagnosis despite having symptoms that meet diagnostic criteria. However, self-reported race/ethnicity of Asian (OR 0.40 [95% CI 0.32 - 0.50]), Hispanic (OR 0.79 [95% CI 0.69 - 0.90]), or multiple/other races (OR 0.88 [95% CI 0.80 - 0.97]); being obese (obesity 1: OR 0.65 [95% CI 0.59 - 0.71], obesity 2: OR 0.49 [95% CI 0.44 - 0.55], obesity 3: OR 0.34 [95% CI 0.31 - 0.38]); or reporting a family history of PCOS (OR 0.23 [95% CI 0.21 - 0.26]) at baseline increased the odds of reporting having received a PCOS diagnosis (**Table 3**).

#### Sensitivity analyses

The analysis comparing the three subsets of participants (completed different quantities of surveys relevant to the possible PCOS definition) revealed minor differences (**Supplementary Table S5**). Subset 1 was very similar to Subset 2. The participants in Subset 3 were slightly older, more educated, of higher SES, and reported Non-Hispanic White more frequently. However, all proportion differences between subsets were less than 3.2%. Of note, BMI and reporting a family history of PCOS were very similar across subsets. Additionally, prolonged time to cycle regularity was very similar between the Subset 2 and 3 (**Supplementary Table S5**).

When analyzing sociodemographic and health characteristics stratified by age, the group of participants ≥50 years at baseline appear to be mostly White (82.6%), of higher SES (50.5% score 6 - 9) and education level (64.1% college degree or higher), and report much better overall health compared to peers their age (24.8%) compared to the other age groups (**Supplementary Table S6**). Additionally, the older age group at baseline (≥50 years) reported healthier lifestyle trends (greater fruit and vegetable consumption, greater exercise minutes, less stress, less smoking, greater quantity of 7 – 9 hours of sleep per night) compared to younger participants (**Supplementary Figures S2** and **S6**).

The selected lab biomarker concentrations by PCOS status among the subset of participants with lab biomarker data linked from their health records can be found in **Supplementary Table S7**. The possible PCOS group had the highest free T values of the three groups (PCOS: 2.1 [2.1 - 6.8] pg/mL, possible PCOS: 3.6 [1.7 - 6.9] pg/mL, no PCOS: 1.6 [1.2 - 3.0] pg/mL) and had elevated total T values compared to the no PCOS group (PCOS: 36.0 [24.8 - 52.3] ng/dL, possible PCOS: 31.0 [21.5 - 95.0] ng/dL, no PCOS: 26.0 [18.0 - 45.4] ng/dL). Additionally, the possible PCOS group had the lowest estradiol levels (PCOS: 55 [35 - 99] pg/mL, possible PCOS: 44 [24 - 84] pg/mL, no PCOS: 73 [39 - 126] pg/mL). TSH and prolactin levels were similar across all groups (TSH PCOS: 1.8 [1.2 - 2.9] µIU/mL, possible PCOS: 2.1 [1.3 - 4.5] µIU/mL, no PCOS: 2.0 [1.2 - 3.4] µIU/mL; prolactin: PCOS: 11.8 [7.5 - 16.7] ng/mL, possible PCOS: 11.7 [6.7 - 15.4] ng/mL, no PCOS: 11.7 [8.1 - 17.6] ng/mL). 17-OHP was highest in the PCOS group (53 [31 - 77] ng/dL), followed by the possible PCOS group (45 [37 - 66] ng/dL), then the no PCOS group (39 [30 - 63] ng/dL).

## Discussion

By identifying participants with PCOS, possible PCOS, and without PCOS in a large, prospectively designed, digital application-based cohort, we were able to cross-sectionally assess sociodemographic, health, and lifestyle characteristic differences throughout the lifespan by PCOS status. We also evaluated time of diagnosis for PCOS related health conditions. The results of our main analyses, paired with our secondary analysis of what characteristics are at play in not reporting a PCOS diagnosis when presenting with the relevant clinical manifestations, may be used to inform the clinical model of care.

### Sociodemographic characteristics

There were minimal racial/ethnical differences by PCOS status in our study. However, a previous study using electronic health record (EHR) data reported a more diverse possible PCOS population (lower percentage of White individuals and higher percentage of Black individuals) compared to the diagnosed PCOS group (24). The study did not include individuals without PCOS, limiting further comparisons (24). The AWHS consists of participants that own an iPhone, provided informed consent for participation in the study, were the sole user of the phone, had access to the internet, and participated without monetary compensation, which may lead to selection bias and lack of generalizability not seen in a hospital EHR cohort, particularly in a safety net hospital like that used in Silva et al. (24, 27). Lower subjective SES and lower educational obtainment for the PCOS and possible PCOS group had not yet been detected in the literature, but one study reported fewer participants with PCOS having an annual household income of >$70,000 (51.5%) when compared to possible PCOS (58.7%) and those without PCOS (67.8%) (20). However, previous literature does compare SES among PCOS participants to no PCOS participants. Low SES has been associated with adverse health behaviors that are connected to PCOS sequalae and symptoms, such as obesity, decreased treatment management, and geographic locations which could be related to environmental exposure disparities. Therefore, the gap in SES between our PCOS and no PCOS group is not surprising (38).

### Health and lifestyle conditions

Our results emphasize that several comorbid health conditions are prevalent across the life course of individuals with PCOS, an expanded perspective relative to the traditional yet narrow focus of PCOS only affecting women during their reproductive years. The detected health and lifestyle differences for those with PCOS compared to those without PCOS are consistent with previous literature (39). However, our additional analysis of a possible PCOS sub-cohort contributes to the literature by potentially highlighting a population that could be served by counseling to reduce unfavorable health outcomes. We have identified some trends that need to be explored in future work to better understand the possible mechanisms of these findings.

Age at menarche was not found to be significantly different in previous literature that compared PCOS/possible PCOS status, and time to cycle regularity was not assessed (20, 23). While our analysis detected frequency differences for all assessed health conditions when stratified by PCOS status, previous literature reported health differences only for gestational glucose tolerance for index pregnancy (gestational diabetes mellitus, impaired glucose tolerance, transient hyperglycemia, and normoglycemia), obesity/BMI, infertility, high blood pressure, high cholesterol, high blood sugar, and clinical depression (p<0.05) (20, 23). No mental health conditions other than depression have been investigated when stratified by PCOS and including possible PCOS as an independent group (23). Additionally, we were the first, to our knowledge, to detect pregnancy complications at different frequencies by PCOS status (20, 23).

We were able to compare age at diagnosis by PCOS status, including possible PCOS, and our results are in line with the minimal existing previous literature that assessed age at diagnosis for cardiometabolic and metabolic conditions. A recent analysis using the same AWHS cohort found similar differences in the age of diagnosis for the onset of cardiometabolic conditions when comparing PCOS to no PCOS groups (40). Although our definition of metabolic syndrome varied compared to previous literature, Peng et al. predicted an earlier age at onset for the PCOS group (included possible PCOS cases) compared to the no PCOS group (48.7 years v. 51.5 years). Additionally, Hillman et al. reported a higher relative risk for metabolic syndrome for those with PCOS that were younger than 20 years compared to those aged 20 – 34 years, stratified by race (<20 years White: RR 3.72 [1.90 - 7.25]; Black: RR 10.13 [5.10 - 20.13]) (10). Vine et al. detected earlier age at diagnosis differences when comparing PCOS to no PCOS participants for conditions we did not study (circulatory diseases [3 – 4 years earlier], dyslipidemia [3 years earlier], Parkinson’s/unspecific dementia [19 years earlier]). Unlike our study, Vine et al. found congestive heart failure was reported 1 year earlier for no PCOS patients and found no difference among T2DM age at diagnoses between groups (8). Previous literature incorporated lab values, medications, waist circumference, and International Classification of Disease (ICD) codes in their metabolic syndrome definition whereas we used self-reported components of metabolic syndrome (8–10). Lastly, also in line with our findings, in a meta-analysis using the results of 10 studies, endometrial cancer risk increased among PCOS patients as age decreased (<54 years: OR 5.14 [3.22, 8.21]; no age exclusion: OR 4.07 [2.13, 7.78]) (41).

Our study is the first, that we know of, to analyze any lifestyle behaviors stratified by PCOS status when including possible PCOS, and we did such in a multi-faceted way exploring variables of nutrition, physical activity, stress, substance use, and sleep. We observed that those with diagnosed PCOS have slightly less healthy lifestyle choices followed by the possible PCOS group when compared to the no PCOS group (i.e., higher daily marijuana and e-cigarette use, lower exercise minutes/vigor, too little/much sleep, high stress) with the exception of diet and alcohol use for the diagnosed PCOS group. While we cannot infer causality from our analytical data, it is possible that those who received a PCOS diagnosis are potentially modifying their dietary choices and alcohol consumption which could represent learned behavior influenced by advice from healthcare providers after receiving a diagnosis. In a previous study surveying 493 women with PCOS collecting information on lifestyle modifications, 82% of women reported altering their diet and 73% reported regular exercise of which 60% reported exercising to manage their PCOS. However, despite effort to modify, only 13% reported achieving their self-determined health goal (42). When assessing lifestyle characteristics, it is important to recognize other variables at play influencing behavioral choice. For example, in the survey conducted by Arentz et al., those that did not modify their exercise behavior reported embarrassment, financial cost, and time constraints as barriers to exercising (42).

### Secondary analysis: possible PCOS

While we found those who reported Asian or Hispanic race/ethnicities were more likely to report having received a PCOS diagnosis when presenting clinical symptoms, Silva et al. found that Black/African American participants were more likely to have a missed PCOS diagnosis (OR:1.69 [1.28,2.24]). As previously mentioned, the cohort demographics of the AWHS may play a role in differing results to Silva et al. To our knowledge, education level had not been found to increase the odds of receiving a PCOS diagnosis (24). Previous PCOS awareness studies found that education level, particularly higher education in a medical field, was a strong predictor in PCOS awareness. Additionally, experiencing PCOS symptoms was associated with higher awareness scores, and many participants learned about PCOS symptoms through family members (43–45). This aligns with our finding of a higher odds of reporting having received the diagnosis when reporting a family history of PCOS. PCOS awareness, via education level or heritability, could link to patient understanding, advocacy, and willingness to seek care, thus increasing the odds of reporting having received a diagnosis.

Obesity and/or insulin and glucose homeostasis are common PCOS comorbidities. With the relationship between PCOS and increased risk of obesity, as expected, we see that patients in this cohort with higher BMI have higher odds of reporting having received the diagnosis (46). Lean PCOS, those with a PCOS diagnosis and lower BMI, were less likely to report having received the diagnosis which is a critical finding when rethinking the clinical care model, especially because those with possible PCOS still reported cardiometabolic conditions more than no PCOS participants. This is a potential detected clinical bias that was not captured in previously published results (24). Silva et al., however, reported a higher prevalence of under/normal weight for the possible PCOS group and higher prevalence of overweight and obese for the diagnosed PCOS group that was not seen in other studies (23, 24). Similarly and in line with our findings, previous literature reported a higher prevalence of obese BMIs in the PCOS and possible PCOS groups when compared to the no PCOS group (20, 23).

Of note, previous work used the Agency for Toxic Substances and Disease Registry Social Vulnerability Index (SVI) which incorporates other individual-level metrics beyond SES such as transportation availability, housing type, and healthcare access (24). Our subjective measure of SES may not capture the nuance of participant vulnerability, and additional metrics could provide a more comprehensive assessment of vulnerability and odds of a missed diagnosis.

Age was a significant predictor in reporting having received a diagnosis in our model, matching the previously existing models (24), likely due to patients receiving a PCOS diagnosis during an infertility evaluation (23). This is further supported by the high percentage of infertility reported among those with PCOS compared to the other groups in our study. Although those with possible PCOS reported lower infertility diagnoses similar to those without PCOS, they may be experiencing increased time to conception, though we cannot evaluate that in this study. Further supporting this point, those with possible PCOS reported a gravidity of 0 more than the other groups despite a similar average age at enrollment.

Lastly, those assigned to the possible PCOS category in our study may not fit full PCOS diagnostic criteria, and a formal clinical evaluation would be necessary. Those with possible PCOS may have other conditions that present with similar symptoms or sequalae, such as hypothalamic amenorrhea, non-classic congenital adrenal hyperplasia (NC-CAH), hypercortisolemia, hypogonadism, primary ovarian insufficiency (POI), and hypo-/hyperthyroidism (47). However, the biomarker comparison data by PCOS status may help us confirm that some of the endocrinopathies (i.e., hypothyroidism as reflected by TSH levels, hyperprolactinemia as reflected by prolactin levels) are unlikely to be contributing to the possible PCOS cases identified. Not all endocrinopathies, such as NC-CAH or POI, can be ruled out, and the biomarker distributions are a small subset. Additionally, research has shown that patients presenting with persistent irregular cycles experience health outcomes like ones discussed in this paper. So, if a participant with possible PCOS does not have PCOS but rather a different health condition, seeking clinical evaluation could be beneficial to minimize health effects later in life (48).

### Strengths and limitations

This study was the first, to our knowledge, to detect certain health and any lifestyle characteristic differences by PCOS status when including a possible PCOS group. Previous studies that compared sociodemographic and health characteristics of PCOS/possible PCOS/no PCOS groups had smaller cohorts compared to the AWHS (other studies PCOS: n=56, n=54, possible PCOS: n=64, n=51 v. AWHS PCOS: n=11,022, possible PCOS: n=7,152) (20, 23). Another strength includes the wide participant age range to assess characteristics across the lifespan. The diverse survey questions of the cohort also allowed us to evaluate multiple health and lifestyle characteristics associated with PCOS across the life course, from puberty to post menopause. Lastly, participants came from an unselected population and without clinical bias, which could help diversify the PCOS experiences compared to clinical cohorts (46, 49).

While our results demonstrate sociodemographic, health, and lifestyle characteristic differences among participants by PCOS status, our study is limited regarding identification of the underlying sociopsychological determinants of health. Nonetheless, it is crucial to consider potential underlying beliefs and behavioral patterns related to individual and structural factors that may drive and sustain these differences. One framework to interpret our work includes the health belief model, which explains how individuals perceive health threats and how the threats influence their behavior (50). Those with possible PCOS may perceive their symptoms as less severe or may encounter greater barriers (e.g., stigma, lack of information, cultural beliefs about menstruation and fertility) that discourage engagement with the healthcare system (20, 51). These barriers can be exacerbated in underserved populations, contributing to underdiagnosis and poorer health outcomes (52, 53). A review article of 16 studies found that across multiple clinical domains, delays to timely diagnosis were at the socioeconomic and sociocultural level (e.g., low health literacy, distrust in health systems, cultural and linguistic barriers), the provider level (e.g., cognitive bias [for instance weight stigmatization], lack of disease knowledge), and the health system level (e.g., administrative barriers, fragmented care environment). Bias within the healthcare system and differences in resource access for different minority groups can create delayed or missed diagnoses and limit engagement in preventive health behaviors (53). Lastly, stigma surrounding PCOS symptoms, such as hirsutism or infertility, may be compounded for certain ethnic or cultural groups, affecting help-seeking behavior, self-efficacy, and mental health. Therefore, it is imperative that the clinical model of care take into consideration psychological and social determinants of health of a patient alongside their biological symptoms.

There are other limitations with a digital application-based epidemiological study. Beyond the previously mentioned selection bias, there is also potential of recall error (misclassification) due to the survey response method. This error is important to note given the assessment of possible PCOS, such as hirsutism, was assigned by self-reported survey responses rather than clinical records (i.e., ICD codes, laboratory values, vital statistics, ultrasounds). Furthermore, survey responses may not capture the ethnic and racial variation of hirsutism (39). Our sensitivity analysis with comparing lab biomarker concentrations by PCOS status supports our consideration of possible PCOS category, but there were limited participants with laboratory values available. Additionally, lifestyle characteristics are multi-factorial, and our survey questions do not offer extensive information to accurately capture a holistic summary of participant lifestyle characteristics. For example, the nutritional assessment used survey questions asking about following a diet and general fruit and vegetable consumption. At the risk of increasing participant burden, a full food and drink intake assessment would more comprehensively capture nutritional lifestyle behaviors. Additionally, physical activity was assessed in minutes per week and did not formally assess heart rate or stratify by type of exercise in our analysis. This is one area where sensor data could be particularly useful in the future. Furthermore, there are health outcomes that are not covered in this review, particularly in senior adulthood, that should be considered in future PCOS studies on health over the life course. The cross-sectional design is another limitation of this study. Some outcomes would be better assessed using a prospective design, for example the BMI variable. Given the results of our sensitivity analysis, the final BMI assessment at age 55 years should be interpreted with caution due to the potential for survival bias. Future work should include a BMI trajectory model to follow participants as individuals over time (54). We also acknowledge the limitation of using BMI which does not discern location or proportions of fat and lean tissue on the body. Other measures of central adiposity, such as waist circumference or imaging, were not assessed in the AWHS. These measurements could be more inclusive and relevant for certain populations, such Asian Americans (36). Our sensitivity analysis using Asian population BMI cutoff threshold attempts to be more inclusive in body weight-health assessment. Previous work considered sociodemographic characteristics (SES) throughout the life course, which we did not capture retrospectively or in our cross-sectional design (55). To best capture life course health, a discrete choice was made to include an older population in this cohort, but due to the cross-sectional design, androgen excess, specifically hirsutism, was reported at enrollment to assess possible PCOS status. We are comfortable using this definition due to previous work out of the AWHS that found as age increased, reporting “a few” chin hairs increased, but reporting “several” or “a lot” of chin hairs (responses used to assess possible PCOS) was consistent across age, suggesting our possible PCOS definition is suitable (28). Finally, there were reduced response rates for surveys relevant to defining possible PCOS. However, our sensitivity analysis comparing the three subsets displayed minor differences, so we decided to compare PCOS, possible PCOS, and no PCOS groups using different cohort sizes. In particular, the similarity in BMI and family history of PCOS suggest the reduced survey response is unlikely to have biased health outcomes.

### Clinical implications

In 2023, the International Evidence-based Guideline for the Assessment and Management of PCOS released recommendations, including 1) strengthening recognition of broader PCOS features (i.e., metabolic risk factors, cardiovascular disease, sleep apnea, psychological features, and adverse outcomes during pregnancy), 2) improving models of care and shared decision making to improve patient experience, alongside greater research, and 3) maintaining emphasis on healthy lifestyle and emotional wellbeing. As previously mentioned, currently, PCOS diagnostics is often centered around fertility care (23); however, our study emphasizes how PCOS impacts health across the lifespan. It is imperative that the improved model of clinical care takes into consideration the effect of PCOS during the entire life course, including an emphasis on lifestyle behaviors. Previous research has reported that exercise changes may not improve PCOS patients’ weight, but a holistic emphasis on healthy behaviors can help overall wellness and management of other chronic conditions (i.e., insulin/glucose homeostasis, lipid profiles) (56). Weaving lifestyle medicine into the improved model of care is imperative for developing a full model of care for PCOS patients. To implement model of care change, information about PCOS can come from clinicians outside of the reproductive, endocrinology, and infertility spaces whom patients would interact with earlier in life, such as pediatricians, possibly due to PCOS driven symptoms and health conditions (i.e., irregular periods, obesity). Additionally, a collaborative effort of physicians, nurse practitioners, physician assistants, and other medical teams can disseminate health information regarding prevention and health maintenance earlier in life, including the incorporation of lifestyle medicine techniques. This may help decrease the high underdiagnosis rate, minimize delay in diagnosis, and overall improve the patient diagnostic experience. By receiving a diagnosis earlier in life, patients can begin treatment/symptom management to minimize health effects later in life. This is particularly important for PCOS patients experiencing infertility; preventative care could help maximize the fertility window (57). Predictive models can be considered in faciliting earlier diagnoses (58) as well as in providing precision risk prediction for PCOS sequalae. Future studies can determine the impact of these models for precision risk prediction, counseling, and health optimization across the life course. Implementation of predictive models within the electronic health records may facilitate early detection for health optimization and risk reduction among patients with PCOS. Ultimately, shifting towards improved models of care requires PCOS health assessment across the life course with consideration of factors relevant to missed diagnoses and the incorporation of lifestyle characteristic modifications to improve the patient experience and health span.

## Data Availability

The data analyzed in this study is subject to the following licenses/restrictions: Aggregated data that support the findings of this study may be available upon reasonable request from the corresponding author & senior author. Any request for data will be evaluated and responded to in a manner consistent with policies intended to protect participant confidentiality and language in the study protocol and informed consent form. Requests to access these datasets should be directed to applewomenshealthstudy@hsph.harvard.edu.
