# Supplementary material for: Utilizing a digital cohort to understand the health burden and lifestyle characteristics across the life course in individuals with polycystic ovary syndrome and possible PCOS
Source: Front Endocrinol (Lausanne). 2025 Sep 19;16:1585628. doi: 10.3389/fendo.2025.1585628 (PMC12491046; doi:10.3389/fendo.2025.1585628)
Supplement: Supplementary file 1 [file DataSheet1.docx]

Supplementary Material

# Supplementary Data

Supplementary Material should be uploaded separately on submission. Please include any supplementary data, figures and/or tables.

Supplementary material is not typeset so please ensure that all information is clearly presented, the appropriate caption is included in the file and not in the manuscript, and that the style conforms to the rest of the article.

# Supplementary Figures and Tables

For more information on Supplementary Material and for details on the different file types accepted, please see [here](https://www.frontiersin.org/guidelines/author-guidelines#supplementary-material).

## Supplementary Figures


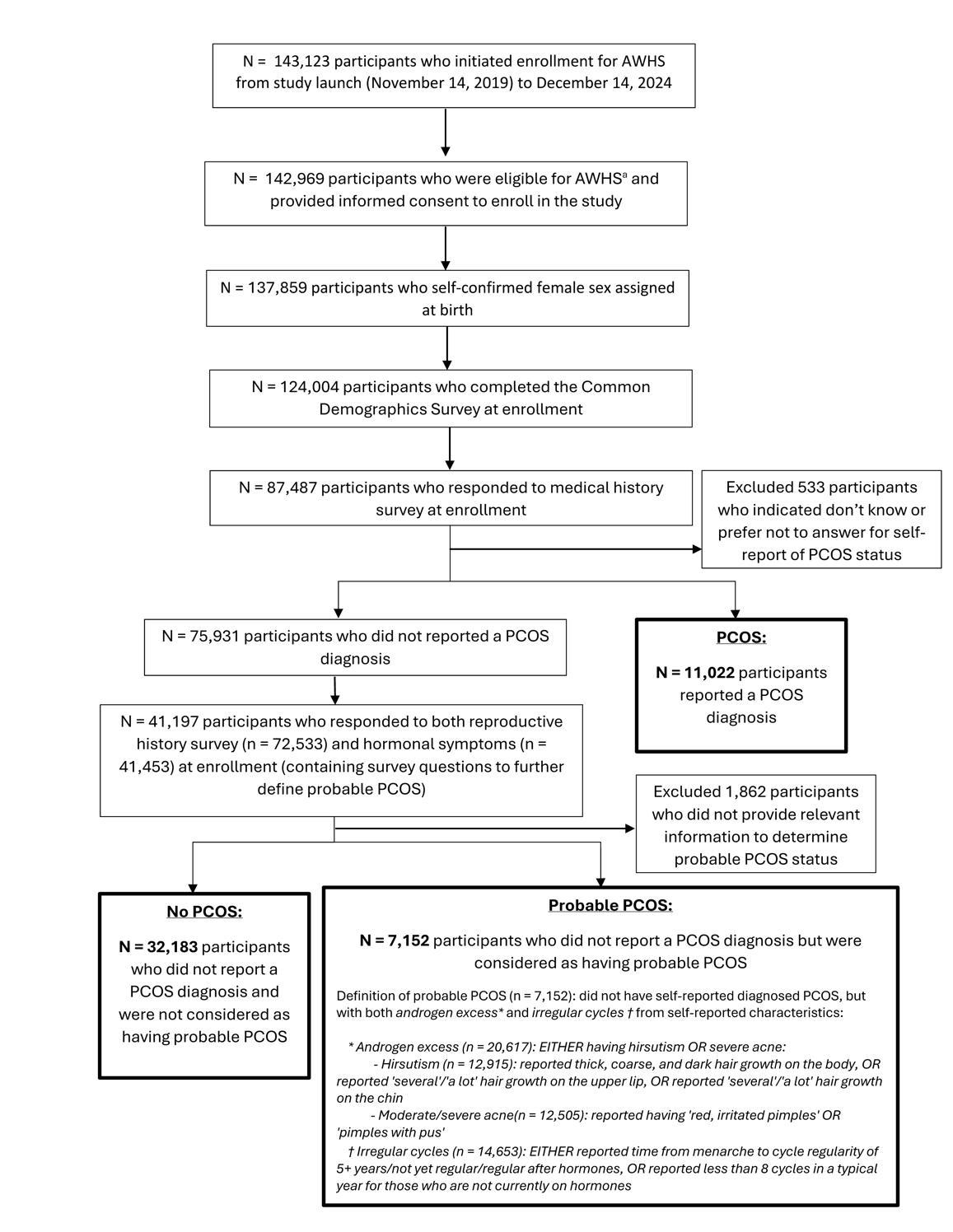


**Supplementary Figure S1.** Flowchart of Apple Women’s Health Study (AWHS) participants in this study.

^a^Eligibility criteria for AWHS include the following: (1) Have menstruated at least once; (2) Be at least 18 years old (at least 19 years old in Alabama and Nebraska, at least 21 years old in Puerto Rico); (3) Live in the United States; (4) Be comfortable in communicating in written and spoken English; (5) Have installed the Apple Research app on an iPhone; (6) Do not share iCloud account or iPhone with anyone else; and (6) Be willing and able to provide informed consent for participation.

**(A)**

**
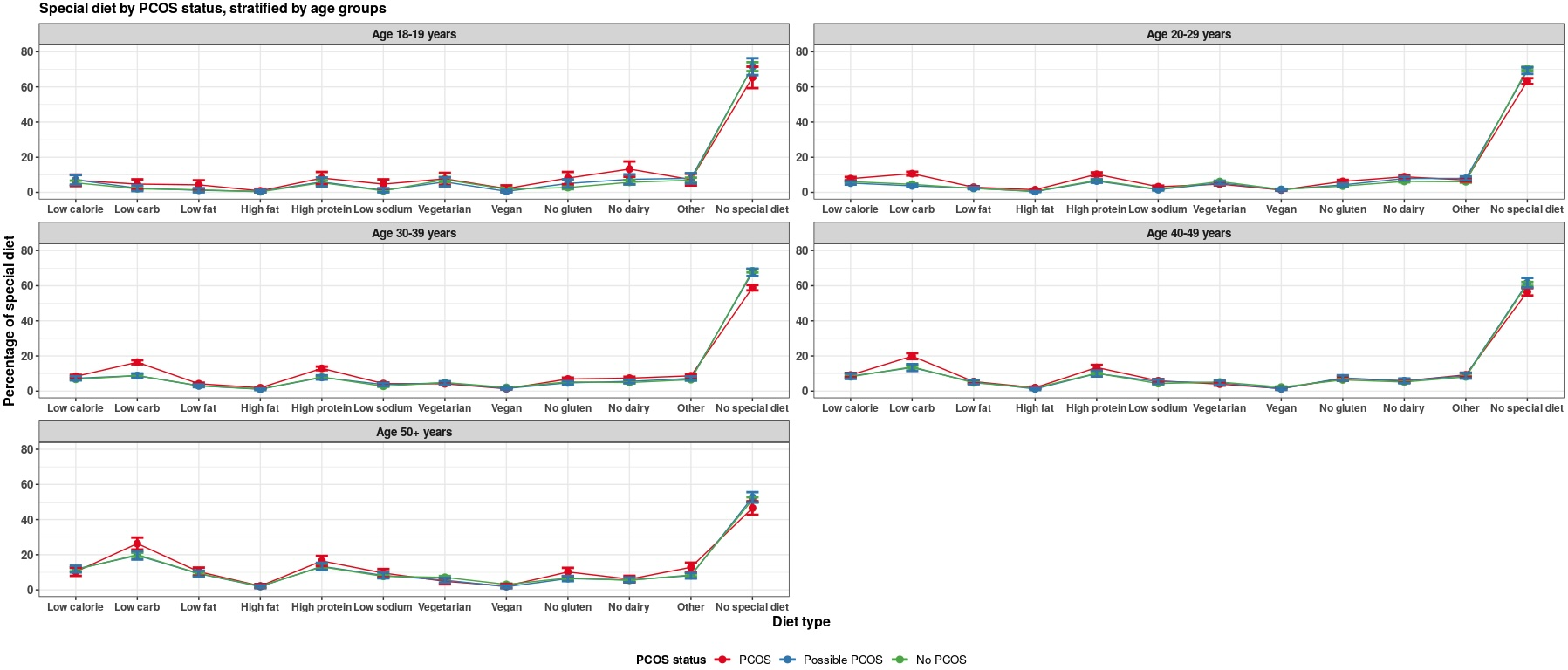
**

**(B)**

**
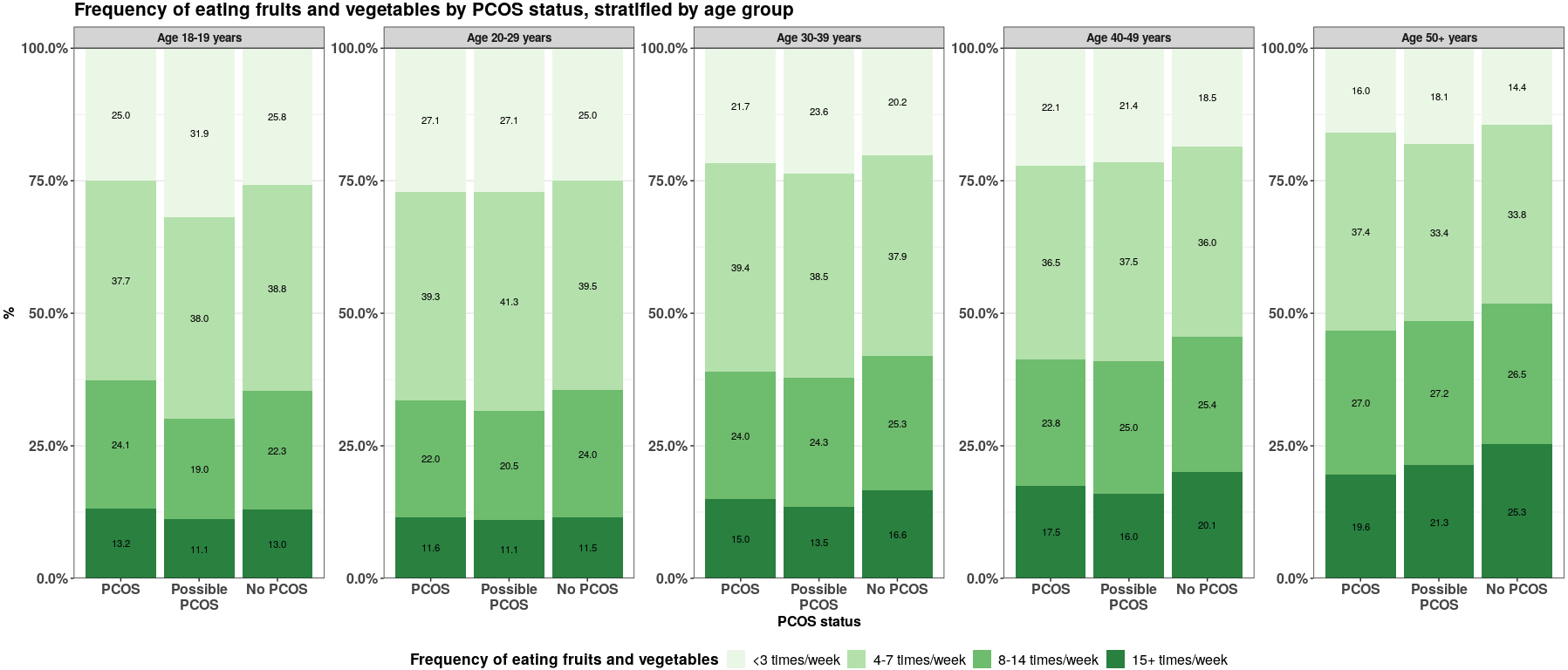
**

**Supplementary Figure S2.** Nutrition by PCOS status, further stratified by age groups.

(A) Special diet patterns by PCOS status, further stratified by age groups; (B) Frequency of eating fruits and vegetables by PCOS status, further stratified by age groups.

Error bars represent 95% CIs.

**(A)**

**
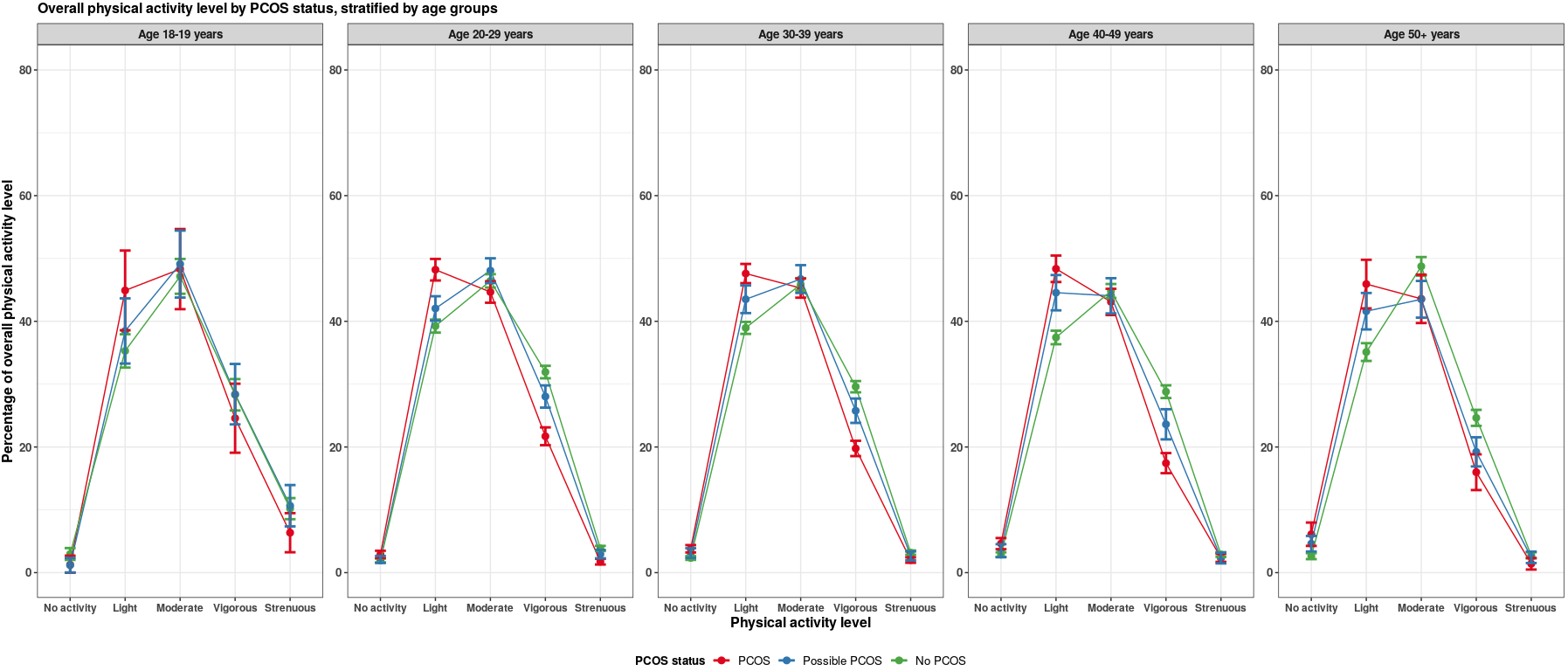
**

**(B)**

**
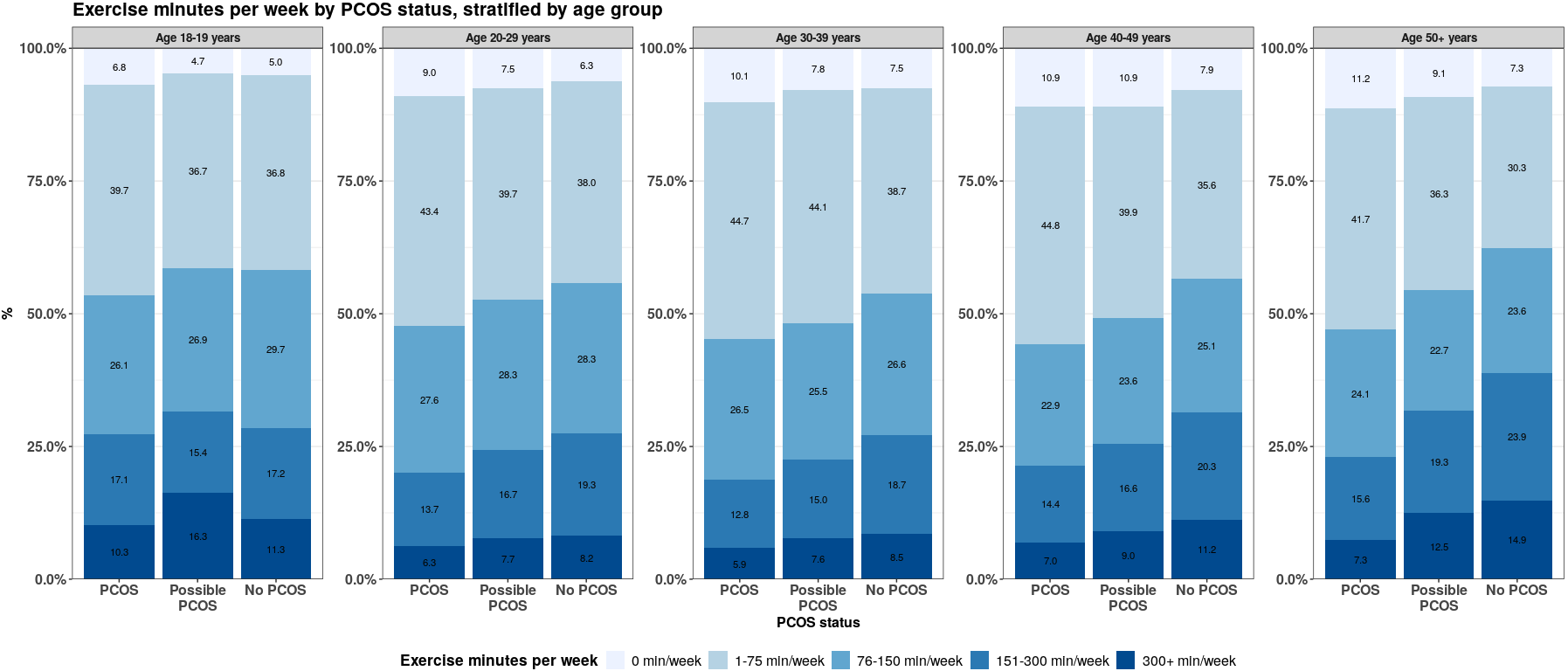
**

**Supplementary Figure S3.** Physical activity by PCOS status, further stratified by age groups.

(A) Overall physical activity levels by PCOS status, further stratified by age groups; (B) Exercise minutes per week by PCOS status, further stratified by age groups.

Error bars represent 95% CIs.

**(A)**

**
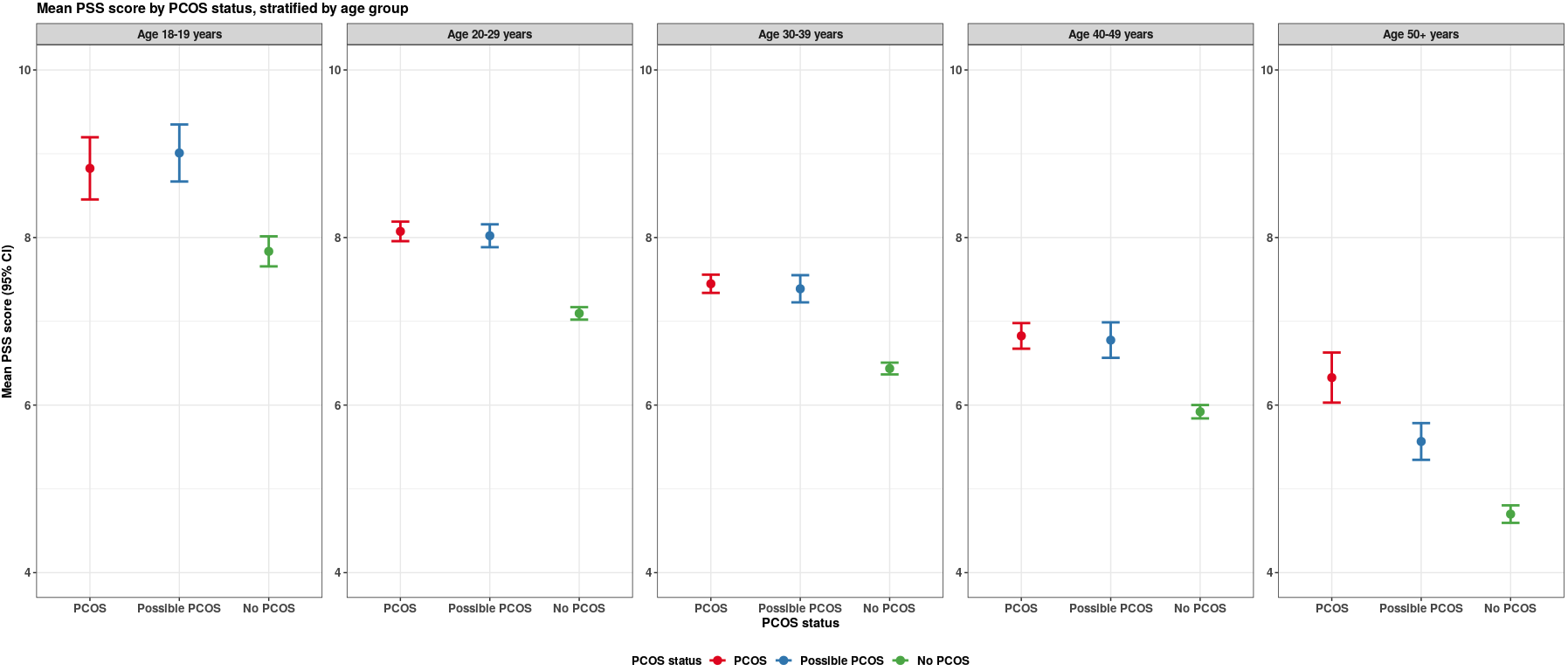
**

**(B)**

**
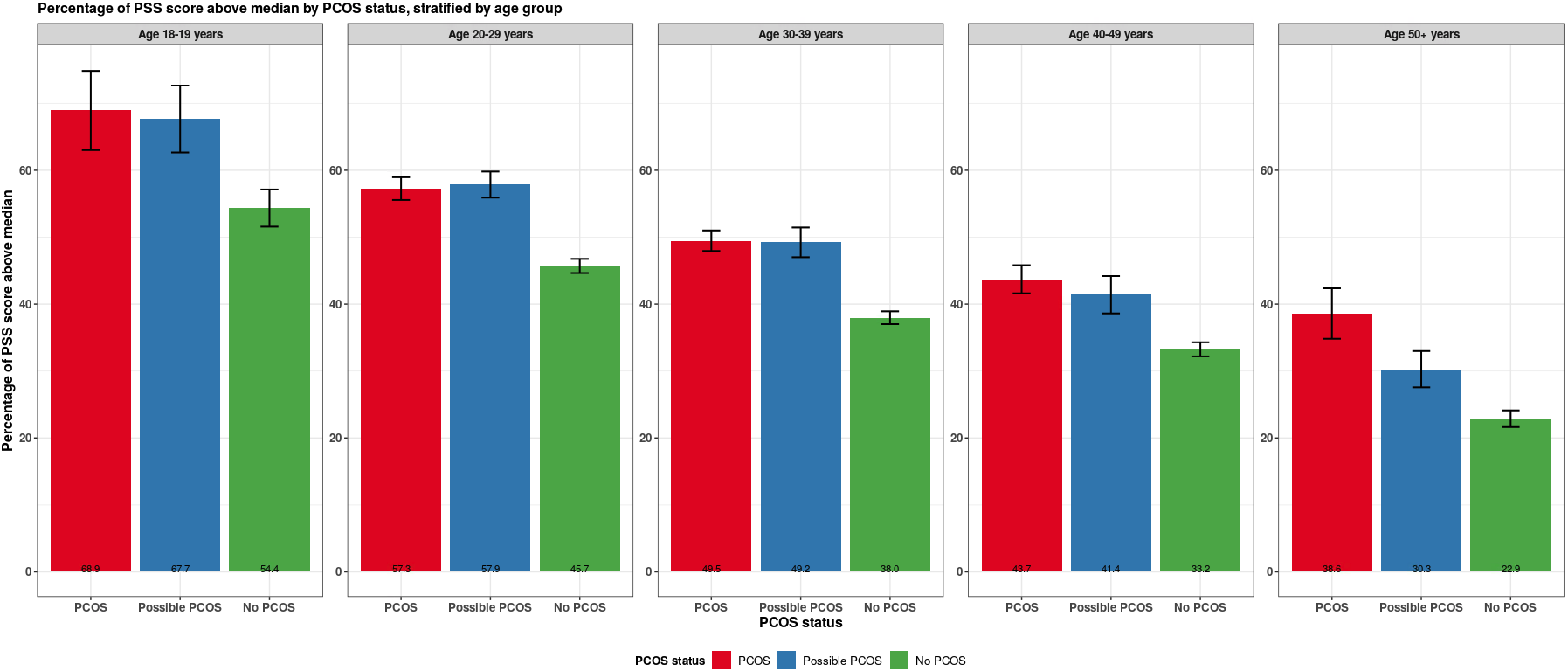
**

**Supplementary Figure S4.** Stress by PCOS status, further stratified by age groups.

(A) Mean PSS-4 score by PCOS status, further stratified by age groups; (B) Percentage of PSS-4 score above the cohort median (=7) by PCOS status, further stratified by age groups.

Error bars represent 95% CIs.

**(A)**

**
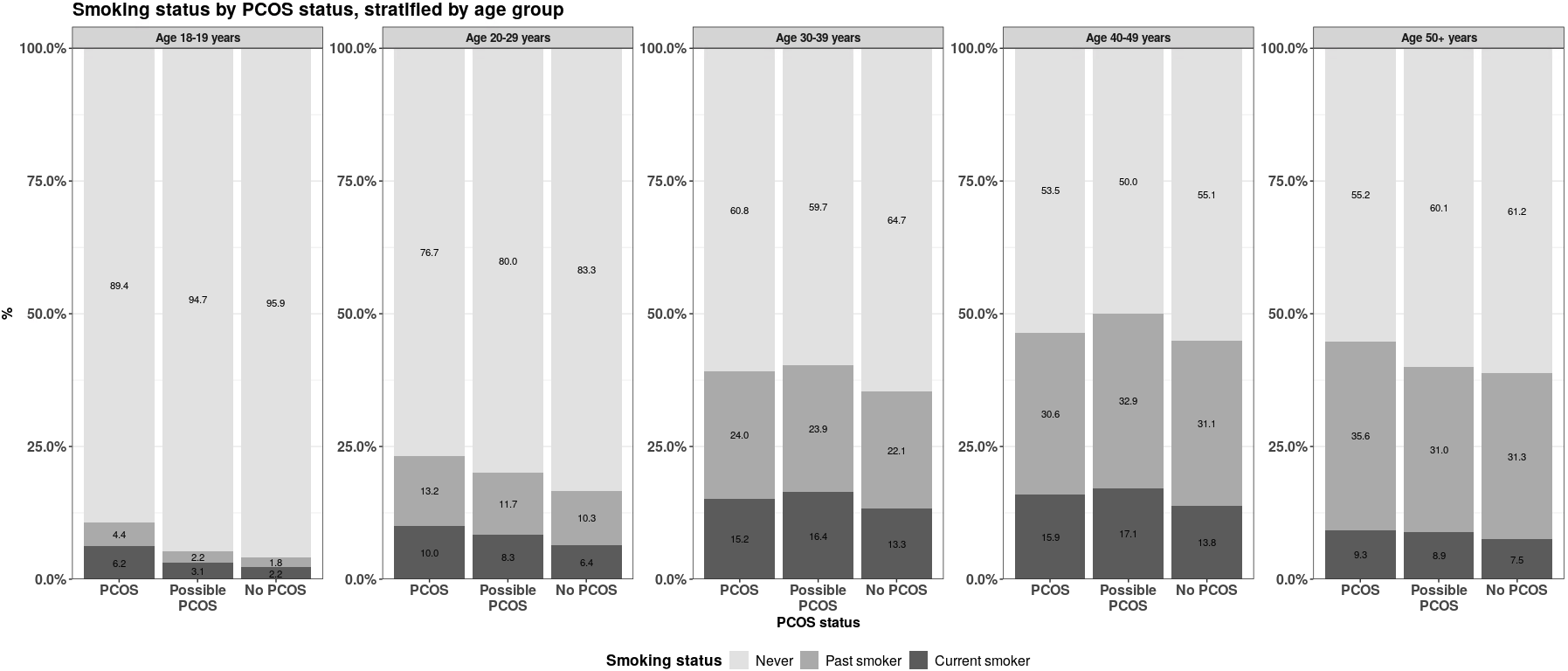
**

**(B)**

**
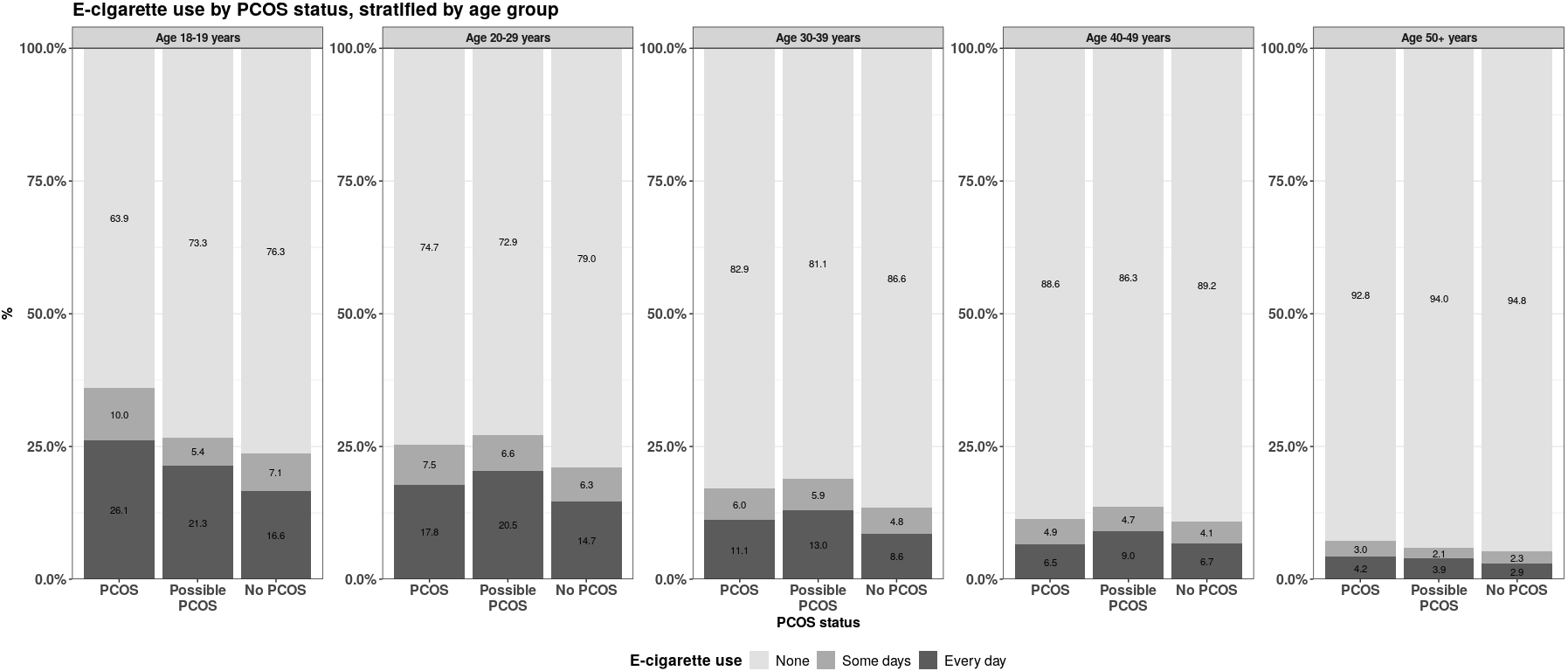
**

**(C)**

**
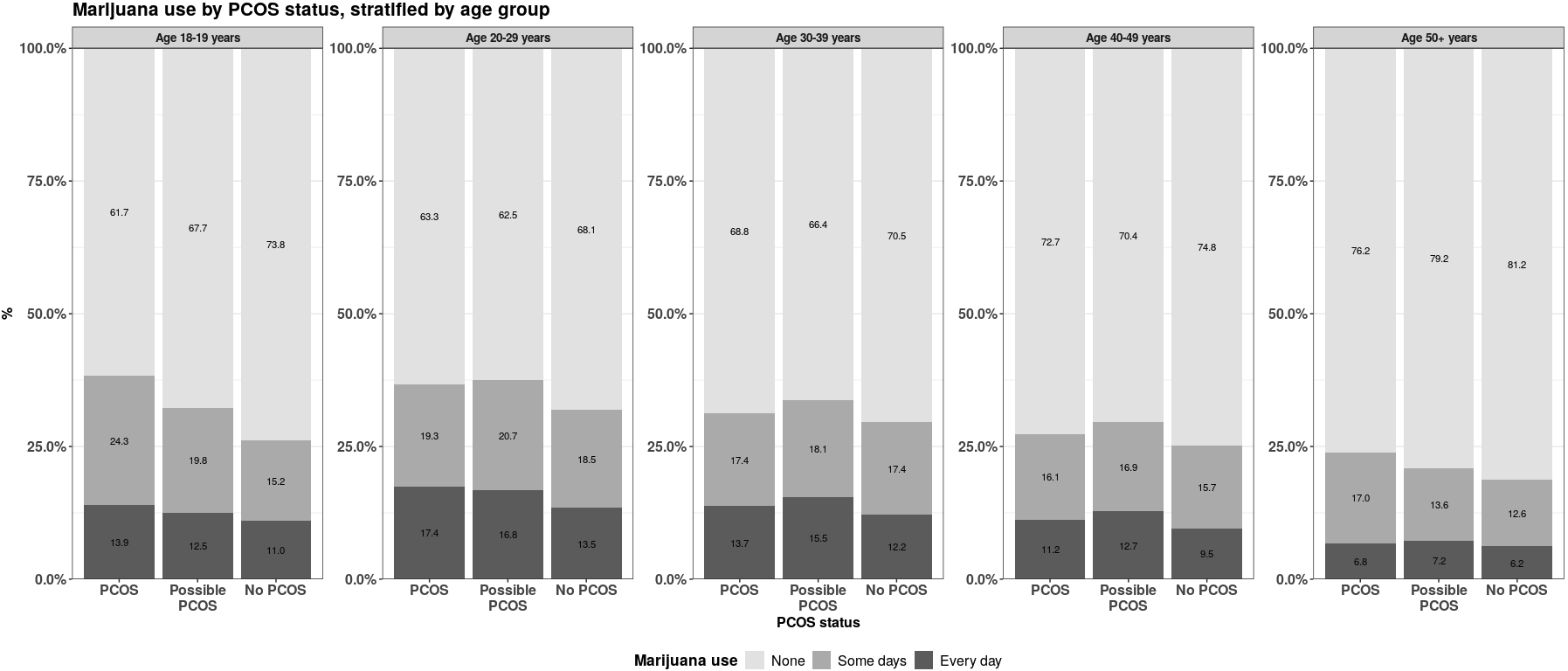
**

**(D)**

**
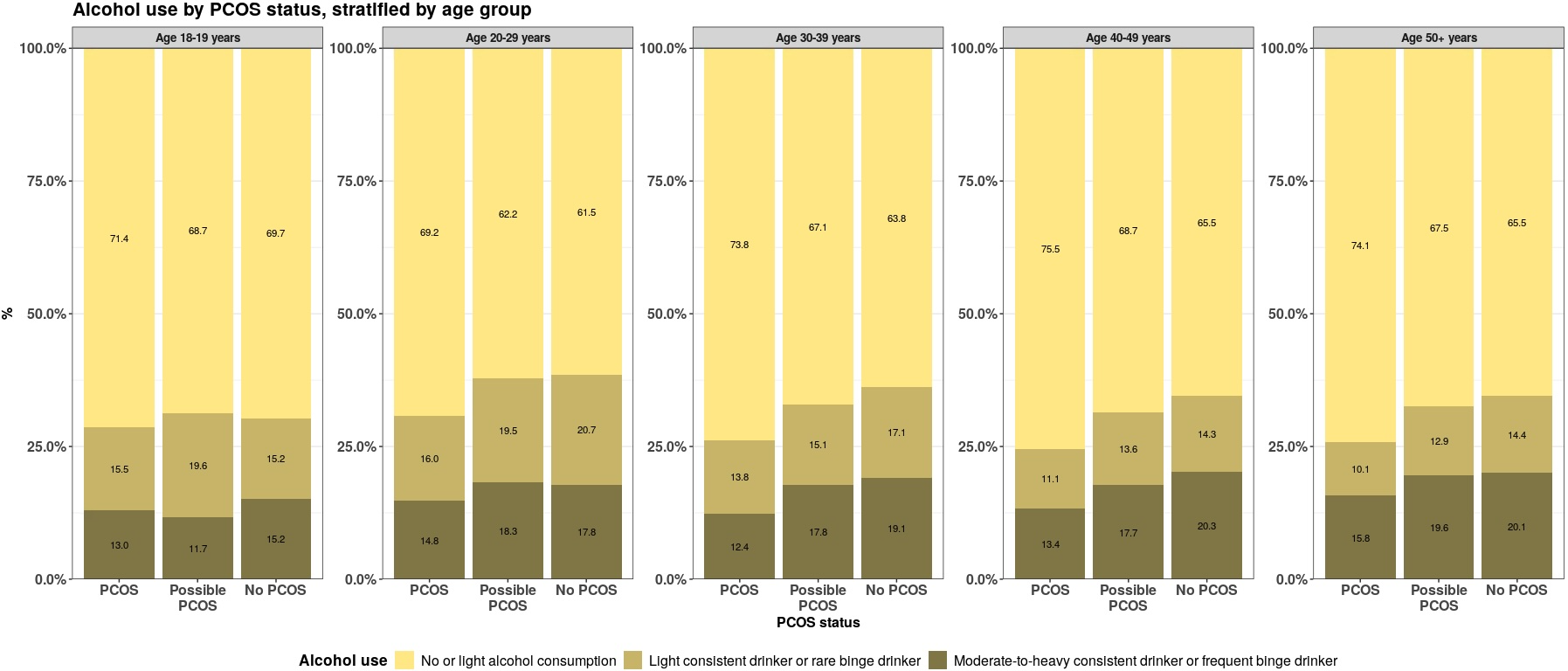
**

**Supplementary Figure S5.** Substance use by PCOS status, further stratified by age groups.

(A) Smoking status by PCOS status, further stratified by age groups; (B) E-cigarette use by PCOS status, further stratified by age groups; (C) Marijuana use by PCOS status, further stratified by age groups; (D) Alcohol use by PCOS status, further stratified by age groups.

**(A)**

**
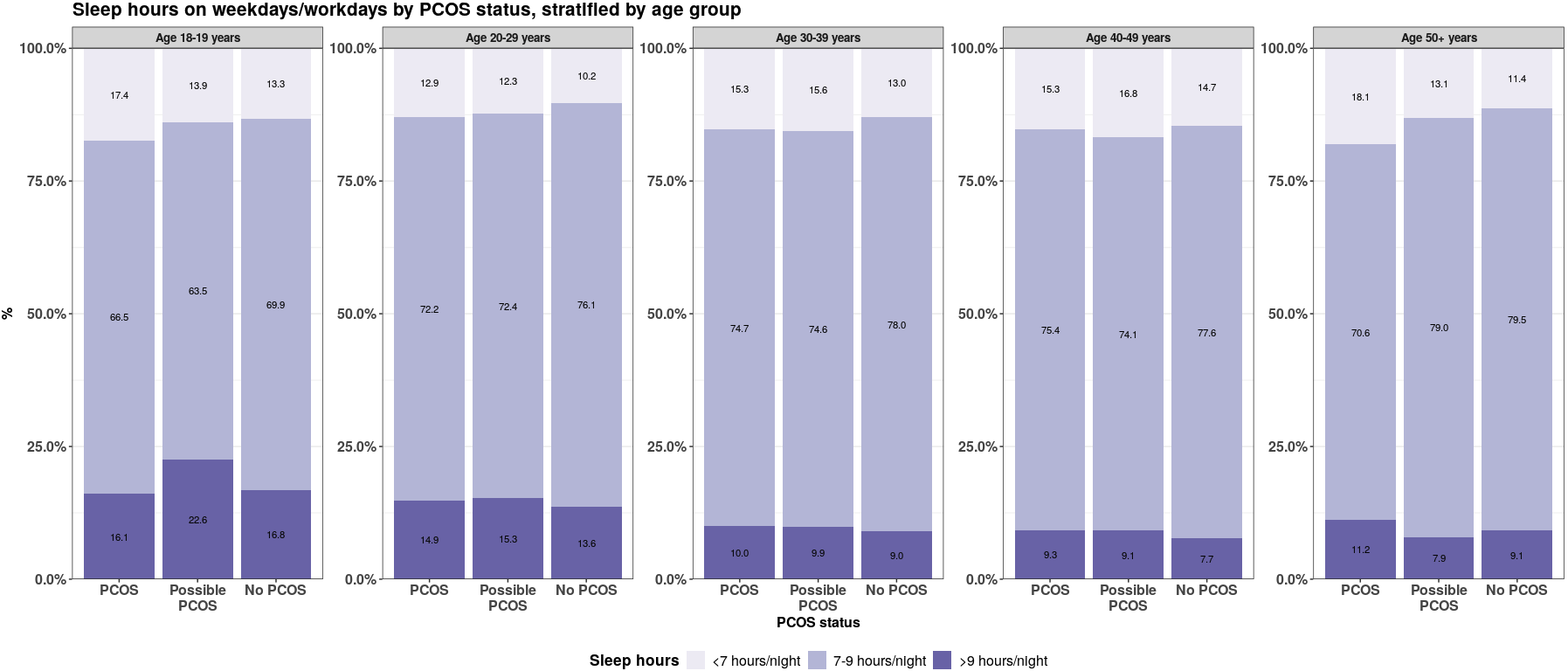
**

**(B)**

**
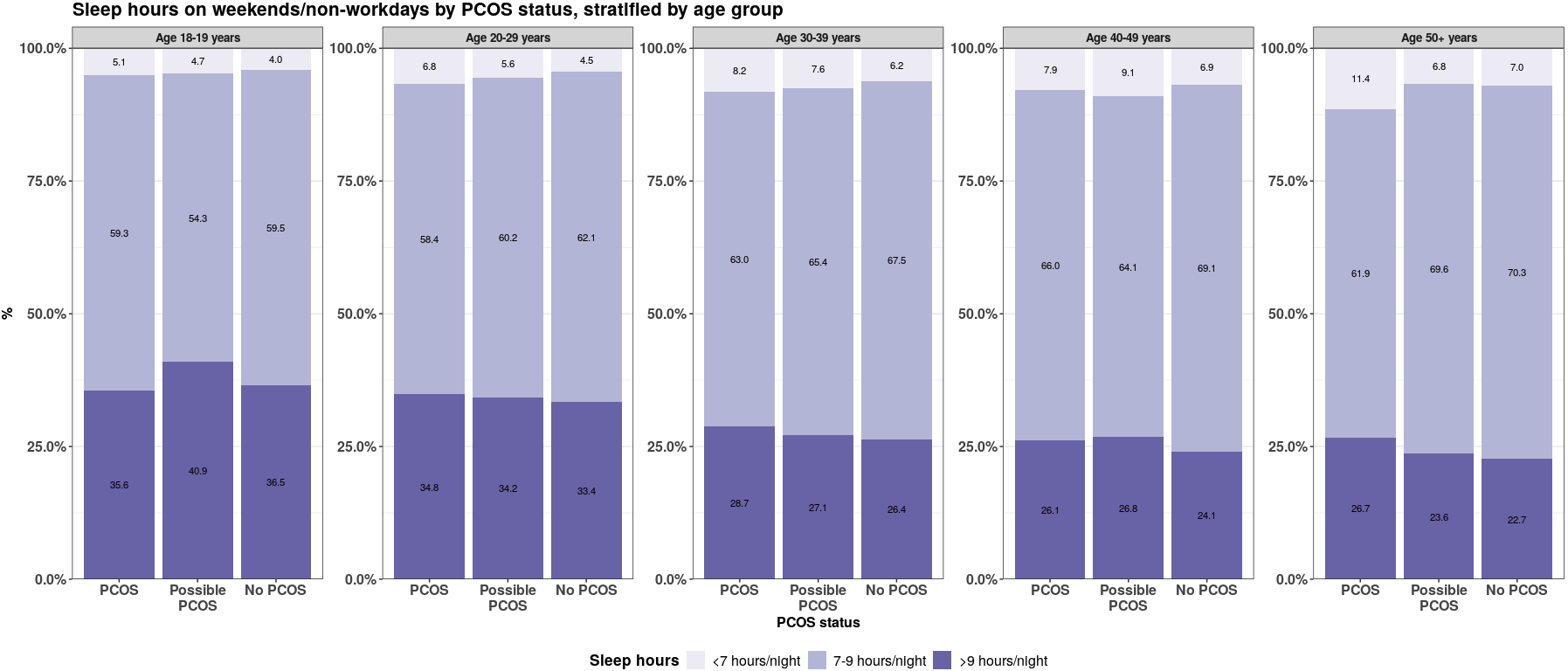
**

**Supplementary Figure S6.** Sleep hours by PCOS status, further stratified by age groups.

(A) Sleep hours on weekdays/workdays by PCOS status, further stratified by age groups; (B) Sleep hours on weekends/non-workdays, further stratified by age groups.

**Supplemental Table S1.** Survey questions asked to Apple Women Health Study participants regarding relevant sociodemographic, health, and lifestyle conditions.

| **Relevant Outcome** | **Survey Question** |
| --- | --- |
| Presumed PCOS: Hirsutism | Do you typically grow thick, course, and dark hair on any of these parts of your body?  Select all that apply.   - Upper lip - Chin - Breasts - Chest between the breasts - Back - Abdomen - Upper arms - Upper thighs - None of the above - I don’t know - I prefer not to answer |
| Presumed PCOS: Hirsutism | How much coarse or thick hair do you have on your upper lip? [ASSOCIATED IMAGES]  Select one.   - None - A few - Several - A lot - I prefer not to answer |
| Presumed PCOS: Hirsutism | How much coarse or thick hair do you have on your chin? [ASSOCIATED IMAGES]  Select one.   - None - A few - Several - A lot - I prefer not to answer |
| Presumed PCOS: Hirsutism | Thinking about your face or back, how would you describe your skin?  Select all that apply   - No acne - Small, raised pimples - Red, irritated pimples - Pimples that have pus - Scars from acne - I don’t know - I prefer not to answer |
| PCOS Family History | Have your biological mother, sister, or daughter ever been diagnosed with any of the following? Select all that apply.   - Breast cancer - Cervical cancer - Cervical dysplasia (pre-cancer) - Colon/rectal cancer - Endometrial cancer (cancer of uterus) - Endometrial hyperplasia (pre-cancer of uterus) - Endometriosis - Fibroids - High cholesterol - Hypertension (high blood pressure) - Infertility - Obesity - Polycystic ovarian syndrome (PCOS) - Premenstrual syndrome/Premenstrual dysphoric disorder (PMS/PMDD) - Thyroid disease - Type 1 diabetes - Type 2 diabetes - None of the above - I prefer not to answer |
| Age at Menarche | At what age did you have your first menstrual period?  It’s okay to estimate.   - 7 years old or younger - 8 years old - 9 years old - 10 years old - 11 years old - 12 years old - 13 years old - 14 years old - 15 years old - 16 years old or older - I don’t know - I prefer not to answer |
| Time to Regularity/Presumed PCOS: Cycle Irregularity | After your first menstrual cycle, how long did it take for your cycle to become regular?  In other words, when could you start predicting the beginning of your next menstrual cycle?   - Less than 1 year - 1-2 years - 3-4 years - More than 5 years - After using hormones (e.g. birth control pills) - They’re not yet regular - I don't know - I prefer not to answer |
| Presumed PCOS: Cycles per year | In a typical year how many periods would you have?  _________ (numeric: whole number, min: 1, max 24) period(s)   - I don’t know - I prefer not to answer |
| Socioeconomic Status | **Think of this ladder as representing where people stand in the country you live in.** At the **top** of the ladder are the people who are the best off – those who have the most money, the most education, and the most respected jobs. At the **bottom** are the people who are the worst off – those who have the least money, least education, the least respected jobs, or no job. The higher up you are on this ladder, the closer you are to the people at the very top; the lower you are, the closer you are to the people at the very bottom.  Where would you place yourself on this ladder?  Please select where you think you stand at this time in your life relative to other people around you.   - Ladder 0 (worst off) to 10 (best off) |
| Race/ethnicity | Which categories describe you? [Select all that apply.]   - American Indian or Alaska Native - Asian - Black, African American, or African - Hispanic, Latino, or Spanish - Middle Eastern or North African - Native Hawaiian or other Pacific Islander - White - None of these fully describe me - I prefer not to answer |
| Education Level | What is the highest grade or year of school you completed?   - Never attended school or only attended kindergarten - Grades 1 through 4 (Primary) - Grades 5 through 9 (Middle school) - Grades 9 through 11 (Some high school) - Grade 12 or GED (High school graduate) - 1 to 3 years after high school (Technical school) - 1 to 3 years after high school (Some college, Associate’s degree) - College 4 years or more (College graduate) - Master’s degree - Doctorate degree - I prefer not to answer |
| Infertility | Have you ever been diagnosed with any of the following by a doctor or other care provider?  Select all that apply.   - Abnormal Pap smear - Adenomyosis - Endometriosis - Fibroids - Infertility - Polycystic ovarian syndrome (PCOS) - Polyps (Uterus or cervix) - Premenstrual syndrome (PMS) or Premenstrual dysphoric disorder (PMDD) - None of the above - I prefer not to answer |
| BMI at enrollment | What is your height?  _________ (Feet, inches) **units relevant to iPhone setting**   - I prefer not to answer   What is your current weight?  _________ (numeric: whole number, min: 40, max 800) pounds |
| BMI history | What was your height when you were 18 years old?  It’s okay to estimate.  _________ (Feet, inches)   - I prefer not to answer     What was your weight when you were 18 years old?  If you were pregnant at this time, estimate your weight prior to that pregnancy.  _________ (numeric: whole number, min: 40, max 800) pounds   - I prefer not to answer     What was your weight when you were 25 years old?  If you were pregnant at this time, estimate your weight prior to that pregnancy.  _________ (numeric: whole number, min: 40, max 800) pounds   - I prefer not to answer or not applicable   What was your weight when you were 35 years old?  If you were pregnant at this time, estimate your weight prior to that pregnancy.  _________ (numeric: whole number, min: 40, max 800) pounds   - I prefer not to answer or not applicable   What was your weight when you were 45 years old?  If you were pregnant at this time, estimate your weight prior to that pregnancy.  _________ (numeric: whole number, min: 40, max 800) pounds   - I prefer not to answer or not applicable     What was your weight when you were 55 years old?  It’s okay to estimate.  _________ (numeric: whole number, min: 40, max 800) pounds   - I prefer not to answer or not applicable |
| Pregnancy History | How many times have you been pregnant? If you are currently pregnant, do not include this pregnancy.  _____ time(s) (numeric: whole number, min: 0, max: 50)   - I prefer not to answer |
| Pregnancy: Artificial Reproductive Technology | Did you conceive with help of any of these methods? Select all that apply.   - Yes, with artificial insemination - Yes, with medication - Yes, with IVF - No (exclusive choice) - I don’t know - I prefer not to answer |
| Pregnancy Complications | Did you have any complications related to this pregnancy? Select all that apply.   - Gestational diabetes (diabetes only during pregnancy) - Gestational hypertension (high blood pressure only during pregnancy) - Preeclampsia or eclampsia (high blood pressure leading to other complications) - Heart problems (heart failure or heart attack) - Postpartum hemorrhage (very heavy bleeding during delivery) - Anemia (low blood count in pregnancy) - Placental abruption (separation of the placenta from uterus) - Placenta previa (placenta covering the cervix) - Intrauterine growth restriction (fetus was too small for weeks of pregnancy) - Perinatal depression (depression during or after pregnancy) - Hysterectomy (surgery to remove your uterus) - Severe infection or sepsis - Seizure disorder - None of the above - I prefer not to answer |
| Metabolic conditions | Have you ever been diagnosed with any of the following by a doctor or other care provider. Select all that apply.   - Hyperprolactinemia (high prolactin levels) - Hyperthyroidism (high thyroid levels) - Hypothyroidism (low thyroid levels) - Prediabetes - Type 1 diabetes - Type 2 diabetes - Vitamin D deficiency - None of the above - I prefer not to answer |
| Cardiometabolic conditions | Have you ever been diagnosed with any of the following by a doctor or other care provider? Select all that apply.   - Arrhythmia, such as atrial fibrillation (AFib) or atrial flutter - Congenital heart disease - Congestive heart failure - Coronary artery disease (CAD) - Heart attack - Heart valve disease - High cholesterol - Hypertension (high blood pressure) - Stroke - Transient ischemic attack (mini-stroke) - None of the above - I prefer not to answer |
| Endometrial cancer/hyperplasia | Have you ever been diagnosed with any of the following by a doctor or other care provider? Select all that apply.   - Blood cancer, such as leukemia or lymphoma - Breast cancer - Cervical cancer - Cervical dysplasia (pre-cancer) - Colon or rectal cancer - Endometrial cancer (cancer of uterus) - Endometrial hyperplasia (pre-cancer of uterus) - Ovarian cancer - Thyroid cancer - None of the above - I prefer not to answer |
| Mental Health | Have you ever been diagnosed with any of the following by a doctor or other care provider? Select all that apply.   - Anorexia - Anxiety disorder - Attention deficit & hyperactivity disorder (ADHD) - Autism spectrum disorder - Bipolar disorder - Bulimia - Depression - Panic disorder - Post-traumatic stress disorder (PTSD) - None of the above - I prefer not to answer |
| Overall Health | How would you describe your health compared to other people your age?   - Much better - Slightly better - About the same - Slightly worse - Much worse - I prefer not to answer |
| Age at diagnosis | How old were you when you were diagnosed with this condition? It’s okay to estimate.  _____ years old (numeric: whole number, min: 1, max: 99)   - I prefer not to answer |
| Nutrition | In the past calendar month, how frequently did you eat fruits and vegetables?   - Fewer than 3 times a week - 4-7 times a week - 8-14 times a week - 15 or more times a week - I prefer not to answer |
| Nutrition | Do you follow a special diet? Select all that apply.   - Low calorie - Low carb - Low fat - High fat - High protein - Low sodium - Vegetarian - Vegan - No gluten - No dairy - Other special diet - No special diet - I prefer not to answer |
| Physical Activity | How much exercise do you usually get per week? Include any moderate to vigorous leisure time activity, such as brisk walking, running, cycling, dancing, strength training, or playing soccer.   - None - 1-75 minutes - 76-150 minutes - 151-300 minutes - >300 minutes - I prefer not to answer |
| Physical Activity | How would you describe your overall physical activity level? Select all that apply.   - I don’t do any physical activity (exclusive choice) - I participate in light activities, such as walking or light housework - I participate in moderate activities, such as brisk walking or yard work - I participate in vigorous activities, such as running or carrying heavy loads - I participate in strenuous activities, such as competitive sports or endurance events like marathons - I prefer not to answer |
| Stress | In the last month, how often have you felt that you were unable to control the important things in your life?   - Never - Almost never - Sometimes - Fairly often - Very often - I prefer not to answer |
| Stress | In the last month, how often have you felt confident about your ability to handle your personal problems?   - Never - Almost never - Sometimes - Fairly often - Very often - I prefer not to answer |
| Stress | In the last month, how often have you felt that things were going your way?   - Never - Almost never - Sometimes - Fairly often - Very often - I prefer not to answer |
| Stress | In the last month, how often have you felt difficulties were piling up so high that you could not overcome them?   - Never - Almost never - Sometimes - Fairly often - Very often - I prefer not to answer |
| Sleep: Weekday/Workday | What time do you usually fall asleep on weekdays or workdays?  value picker: 12:00 am / 1:00 am / … / 11:00 am / 12:00 pm / 1:00 pm / … / 10:00 pm / 11:00 pm / I don’t know / I prefer not to answer  What time do you usually wake up on weekdays or workdays?  value picker: 12:00 am / 1:00 am / … / 11:00 am / 12:00 pm / 1:00 pm / … / 10:00 pm / 11:00 pm / I don’t know / I prefer not to answer |
| Sleep: Weekends/Non-workdays | What time do you usually fall asleep on weekends or non-workdays?  value picker: 12:00 am / 1:00 am / … / 11:00 am / 12:00 pm / 1:00 pm / … / 10:00 pm / 11:00 pm / I don’t know / I prefer not to answer  --  What time do you usually wake up on weekends or non-workdays?  value picker: 12:00 am / 1:00 am / … / 11:00 am / 12:00 pm / 1:00 pm / … / 10:00 pm / 11:00 pm / I don’t know / I prefer not to answer |
| Sleep: Apnea | Have you ever been diagnosed with any of the following by a doctor or other care provider? Select all that apply.   - Asthma - Chronic obstructive pulmonary disease (COPD) - Cystic fibrosis - Sleep apnea - None of the above - I prefer not to answer |
| Sleep: Apnea Symptoms | In the past 12 months, how often did you snort, gasp, or stop breathing while you were asleep?   - Never - Rarely (1-2 nights a week) - Occasionally (3-4 nights a week) - Frequently (5 or more nights a week) - I don’t know - I prefer not to answer |
| Substance Use: Alcohol | How often did you have a drink containing alcohol in the past year? By a “drink,” we mean a can or bottle of beer, a glass of wine or a wine cooler, a shot of liquor, or a mixed drink with liquor in it.   - Never - Monthly or less - Two to four times a month - Two to three times a week - Four or more times a week - I prefer not to answer |
| Substance Use: Alcohol | On a typical day when you drink, how many drinks do you have? By a “drink,” we mean a can or bottle of beer, a glass of wine or a wine cooler, a shot of liquor, or a mixed drink with liquor in it.   - 1 or 2 - 3 or 4 - 5 or 6 - 7 to 9 - 10 or more - I prefer not to answer |
| Substance Use: Tobacco | Have you smoked at least 100 cigarettes in your entire life?   - Yes - No - I don’t know - I prefer not to answer |
| Substance Use: Tobacco | Do you now smoke cigarettes every day, some days, or not at all?   - Every day - Some days - Not at all - I prefer not to answer |
| Substance Use: E-cigarette | Do you now use electronic nicotine products every day, some days, or not at all? Electronic nicotine products include e-cigarettes, vape pens, hookah pens, personal vaporizers and mods, e-cigars, e-pipes, and e- hookahs.   - Every day - Some days - Not at all - I prefer not to answer |
| Substance Use: Marijuana | How often do you currently use marijuana in any form? This includes smoking, electronic cigarettes, vaping products, and edible cannabis products.   - Every day - Some days - Not at all - I prefer not to answer |

**Supplemental Table S2.** Self-reported baseline health characteristics using mean (standard deviation) for continuous variables and number (percentages) for binary/categorical variables, stratified by PCOS/possible PCOS/non-PCOS groups among Apple Women’s Health Study participants enrolled from 11/14/2019 to 12/14/2024 and responded to the relevant survey questions.

| **Self-reported baseline health characteristics^a^** | **PCOS**  **(N = 11,022)** | **Possible PCOS**  **(N = 7,152)** | **No PCOS**  **(N = 32,183)** | **P-value** |
| --- | --- | --- | --- | --- |
| Age at menarche, years, mean ± SD | 12.0 ± 1.8 | 12.2 ± 1.7 | 12.2 ± 1.5 | <.0001 |
| Very early menarche (age <9 years), n (%) | 224 (2.1) | 95 (1.3) | 245 (0.8) | <.0001 |
| Early menarche (age <11 years), n (%) | 1895 (18.0) | 897 (12.7) | 3619 (11.4) | <.0001 |
| Late menarche (age ≥16 years), n (%) | 493 (4.7) | 289 (4.1) | 936 (2.9) | <.0001 |
| Time from menarche to cycle regularity, n (%) |  |  |  | <.0001 |
| Less than 1 year | 2561 (26.1) | 1001 (14.5) | 13999 (51.5) |  |
| 1-2 years | 1267 (12.9) | 493 (7.2) | 6969 (25.6) |  |
| 3-4 years | 437 (4.5) | 131 (1.9) | 2042 (7.5) |  |
| 5+ years | 774 (7.9) | 993 (14.4) | 914 (3.4) |  |
| Not yet regular | 2483 (25.3) | 1755 (25.5) | 1132 (4.2) |  |
| Regular after hormone use | 2276 (23.2) | 2520 (36.6) | 2138 (7.9) |  |
| Infertility diagnosis, n (%) | 1845 (16.7) | 291 (4.1) | 1244 (3.9) | <.0001 |
| Gravidity, n (%) |  |  |  | <.0001 |
| 0 | 4638 (44.3) | 3490 (49.3) | 13013 (40.8) |  |
| 1 | 1685 (16.1) | 937 (13.2) | 4402 (13.8) |  |
| 2 | 1562 (14.9) | 983 (13.9) | 5149 (16.1) |  |
| 3 | 1072 (10.2) | 736 (10.4) | 3966 (12.4) |  |
| 4+ | 1523 (14.5) | 937 (13.2) | 5376 (16.8) |  |
| Any metabolic condition (prediabetes, type 2 diabetes, hypertension, high cholesterol), n (%) | 5024 (45.8) | 2080 (29.2) | 8074 (25.1) | <.0001 |
| Arrhythmia, n (%) | 662 (6.0) | 365 (5.1) | 1252 (3.9) | <.0001 |
| Congestive heart failure, n (%) | 92 (0.8) | 45 (0.6) | 169 (0.5) | <.0001 |
| Coronary artery disease, n (%) | 80 (0.7) | 21 (0.3) | 109 (0.3) | <.0001 |
| Heart attack, n (%) | 100 (0.9) | 49 (0.7) | 151 (0.5) | <.0001 |
| Stroke, n (%) | 103 (0.9) | 67 (0.9) | 213 (0.7) | <.0001 |
| Transient ischemic attack (mini-stroke), n (%) | 183 (1.7) | 75 (1.1) | 295 (0.9) | <.0001 |
| Endometrial cancer, n (%) | 59 (0.5) | 20 (0.3) | 64 (0.2) | <.0001 |
| Endometrial hyperplasia, n (%) | 177 (1.6) | 70 (1.0) | 131 (0.4) | <.0001 |
| Anorexia, n (%) | 527 (4.8) | 404 (5.7) | 1216 (3.8) | <.0001 |
| Anxiety, n (%) | 6825 (62.2) | 4239 (58.1) | 15103 (47.1) | <.0001 |
| Attention deficit & hyperactivity disorder (ADHD), n (%) | 3399 (31.0) | 1882 (26.4) | 6909 (21.6) | <.0001 |
| Bipolar disorder, n (%) | 1518 (13.8) | 823 (11.6) | 2514 (7.8) | <.0001 |
| Bulimia, n (%) | 379 (3.5) | 206 (2.9) | 726 (2.3) | <.0001 |
| Depression, n (%) | 6939 (63.3) | 4167 (58.5) | 15418 (48.1) | <.0001 |
| Panic disorder, n (%) | 1857 (16.9) | 984 (13.8) | 3048 (9.5) | <.0001 |
| Self-evaluated overall health compared to other peers, n (%) |  |  |  | <.0001 |
| Much better | 484 (4.9) | 586 (8.7) | 3691 (12.3) |  |
| Slightly better | 1368 (13.7) | 1302 (19.4) | 7276 (24.3) |  |
| About the same | 3343 (33.5) | 2413 (35.9) | 11215 (37.5) |  |
| Slightly worse | 3534 (35.5) | 1866 (27.8) | 6288 (21.0) |  |
| Much worse | 1238 (12.4) | 557 (8.3) | 1437 (4.8) |  |
| BMI at age 18 (using CDC cutoff values for everyone)^b^, among N = 5,031 participants who provided a recall of this information, mean ± SD | 25.5 ± 8.6 | 22.9 ± 9.2 | 22.1 ± 5.8 | <.0001 |
| Underweight | 130 (12.3) | 130 (19.5) | 621 (18.8) |  |
| Healthy weight | 477 (45.0) | 393 (59.1) | 2066 (62.5) |  |
| Overweight | 232 (21.9) | 71 (10.7) | 398 (12.0) |  |
| Obesity | 222 (20.9) | 71 (10.7) | 220 (6.7) |  |
| BMI at age 25 (using CDC cutoff values for everyone)^b^, among N = 4,766 participants who were ≥25 years old and provided a recall of this information, mean ± SD | 30.2 ± 10.4 | 25.8 ± 11.4 | 24.9 ± 7.2 | <.0001 |
| Underweight | 42 (4.3) | 53 (8.6) | 207 (6.5) |  |
| Healthy weight | 307 (31.4) | 318 (51.5) | 1841 (58.1) |  |
| Overweight | 205 (21.0) | 126 (20.4) | 587 (18.5) |  |
| Obesity | 424 (43.4) | 121 (19.6) | 535 (16.9) |  |
| BMI at age 35 (using CDC cutoff values for everyone)^b^, among N = 3,513 participants who were ≥35 years old and provided a recall of this information, mean ± SD | 32.9 ± 12.8 | 28.4 ± 11.4 | 27.1 ± 8.6 | <.0001 |
| Underweight | 8 (1.3) | 12 (2.7) | 63 (2.6) |  |
| Healthy weight | 142 (22.4) | 184 (41.9) | 1167 (47.8) |  |
| Overweight | 137 (21.6) | 115 (26.2) | 590 (24.2) |  |
| Obesity | 348 (54.8) | 128 (29.2) | 619 (25.4) |  |
| BMI at age 45 (using CDC cutoff values for everyone)^b^, among N = 1,714 participants who were ≥45 years old and provided a recall of this information, mean ± SD | 33.6 ± 12.2 | 29.9 ± 9.0 | 28.2 ± 8.4 | <.0001 |
| Underweight/healthy weight^c^ | 47 (20.1) | 95 (38.6) | 539 (43.7) |  |
| Overweight | 57 (24.4) | 56 (22.8) | 320 (25.9) |  |
| Obesity | 130 (55.6) | 95 (38.6) | 375 (30.4) |  |
| BMI at age 55 (using CDC cutoff values for everyone)^b^, among N = 651 participants who were ≥55 years old and provided a recall of this information, mean ± SD | 31.3 ± 8.7 | 28.6 ± 7.5 | 27.7 ± 8.8 | <.001 |
| Underweight/healthy weight^c^ | 16 (23.5) | 39 (37.5) | 208 (43.4) |  |
| Overweight | 20 (29.4) | 37 (35.6) | 130 (27.1) |  |
| Obesity | 32 (47.1) | 28 (26.9) | 141 (29.4) |  |
| Among N = 27,972 participants with reported gravidity ≥1 and agreed to share pregnancy history information, evaluating pregnancy-related information during 1^st^ pregnancy: | | | | |
| Conceived naturally, n (%)^d^ | 4926 (85.7) | 3412 (96.3) | 17937 (96.4) | <.0001 |
| Conceived with IVF, n (%)^d^ | 167 (2.9) | 30 (0.8) | 226 (1.2) | <.0001 |
| Conceived with artificial insemination, n (%)^d^ | 145 (2.5) | 29 (0.8) | 208 (1.1) | <.0001 |
| Conceived with medications, n (%)^d^ | 640 (11.1) | 86 (2.4) | 334 (1.8) | <.0001 |
| Age at pregnancy, years, mean ± SD | 24.3 ± 5.8 | 23.7 ± 5.5 | 24.5 ± 5.9 | <.0001 |
| No complications, n (%)^e^ | 3398 (59.5) | 2260 (64.1) | 12938 (69.8) | <.0001 |
| Any complications, n (%)^e^ | 2313 (40.5) | 1266 (35.9) | 5598 (30.2) |  |
| Gestational diabetes, n (%)^e^ | 410 (7.2) | 185 (5.2) | 708 (3.8) | <.0001 |
| Gestational hypertension, n (%)^e^ | 452 (7.9) | 205 (5.8) | 820 (4.4) | <.0001 |
| Preeclampsia or eclampsia, n (%)^e^ | 591 (10.3) | 258 (7.3) | 1168 (6.3) | <.0001 |
| Heart problems related to pregnancy, n (%)^e^ | 32 (0.6) | 11 (0.3) | 45 (0.2) | <.001 |
| Postpartum hemorrhage, n (%)^e^ | 255 (4.5) | 156 (4.4) | 560 (3.0) | <.0001 |
| Anemia during pregnancy, n (%)^e^ | 762 (13.3) | 425 (12.1) | 1816 (9.8) | <.0001 |
| Placental abruption, n (%)^e^ | 93 (1.6) | 42 (1.2) | 189 (1.0) | <.001 |
| Placenta previa, n (%)^e^ | 113 (2.0) | 57 (1.6) | 222 (1.2) | <.0001 |
| Intrauterine growth restriction, n (%)^e^ | 146 (2.6) | 69 (2.0) | 280 (1.5) | <.0001 |
| Perinatal depression, n (%)^e^ | 873 (15.3) | 496 (14.1) | 1880 (10.1) | <.0001 |
| Hysterectomy related to pregnancy, n (%)^e^ | 19 (0.3) | 9 (0.3) | 20 (0.1) | <.001 |
| Severe infection or sepsis, n (%)^e^ | 109 (1.9) | 57 (1.6) | 145 (0.8) | <.0001 |
| Seizure disorder related to pregnancy, n (%)^e^ | 59 (1.0) | 19 (0.5) | 52 (0.3) | <.0001 |

Abbreviations: PCOS, polycystic ovary syndrome; SD, standard deviation; BMI, body mass index; CDC, Center of Disease Control and Prevention; IVF, in vitro fertilization.

^a^ Percentages for each variable calculated based on non-missing data. Denominators may be different from total N.

^b^ CDC categorizes BMI as <18.5 (underweight), 18.5 to 24.9 (healthy weight), 25 to 29.9 (overweight), 30.0 to 34.9 (obesity class 1), 35.0 to 39.9 (obesity class 2), and ≥40 kg/m^2^ (obesity class 3).

^c^ Categories aggregated due to small numbers in the relevant underweight category.

^d^ Conceived naturally was an exclusive option. If not selecting this option, participants could select all that apply for conceiving via IVF, artificial insemination, or medication.

^e^ No pregnancy complication was an exclusive option. If not selecting this option, participants could select all that apply for each of the complications listed.

P-values from Chi-square test for categorical outcomes, and from Kruskal-Wallis test for continuous outcomes.

**Supplemental Table S3.** Body mass index (BMI) categories of all among Apple Women’s Health Study participants enrolled from 11/14/2019 to 12/14/2024 and responded to the relevant survey questions, incorporating unique cutoff values for Asian/Asian American participants different from CDC cutoff values.

| **Self-reported characteristics** | **Overall** | **By PCOS status** | | |
| --- | --- | --- | --- | --- |
|  |  | **PCOS** | **Possible PCOS** | **No PCOS** |
| **N** | **50,357** | **11,022** | **7,152** | **32,183** |
| **BMI at enrollment**, kg/m^2^, n (%)^b^ |  |  |  |  |
| Underweight | 1213 (2.4) | 165 (1.5) | 226 (3.2) | 822 (2.6) |
| Healthy weight | 15682 (31.1) | 1909 (17.3) | 2314 (32.4) | 11459 (35.6) |
| Overweight | 12504 (24.8) | 2164 (19.6) | 1794 (25.1) | 8546 (26.6) |
| Obesity | 19992 (39.7) | 6547 (59.4) | 2669 (37.3) | 10776 (33.5) |
| **Among subsets of participants with recalled BMI at ages 18, 25, 35, 45, and 55:** | | | | |
| **BMI at age 18 (N = 5,031):** |  |  |  |  |
| Mean ± SD | 23.0 ± 7.1 | 25.5 ± 8.6 | 22.9 ± 9.2 | 22.1 ± 5.8 |
| n (%)^b^ |  |  |  |  |
| Underweight | 881 (17.5) | 130 (12.3) | 130 (19.5) | 621 (18.8) |
| Healthy weight | 2930 (58.2) | 477 (45.0) | 393 (59.1) | 2060 (62.3) |
| Overweight | 704 (14.0) | 230 (21.7) | 70 (10.5) | 404 (12.2) |
| Obesity | 516 (10.3) | 224 (21.1) | 72 (10.8) | 220 (6.7) |
| **BMI at age 25 (N = 4,766):** |  |  |  |  |
| Mean ± SD | 26.1 ± 8.8 | 30.2 ± 10.4 | 25.8 ± 11.4 | 24.9 ± 7.2 |
| n (%)^b^ |  |  |  |  |
| Underweight | 302 (6.3) | 42 (4.3) | 53 (8.6) | 207 (6.5) |
| Healthy weight | 2456 (51.5) | 304 (31.1) | 317 (51.3) | 1835 (57.9) |
| Overweight | 919 (19.3) | 205 (21.0) | 126 (20.4) | 588 (18.5) |
| Obesity | 1089 (22.8) | 427 (43.7) | 122 (19.7) | 540 (17.0) |
| **BMI at age 35 (N = 3,513):** |  |  |  |  |
| Mean ± SD | 28.3 ± 10.1 | 32.9 ± 12.8 | 28.4 ± 11.4 | 27.1 ± 8.6 |
| n (%)^b^ |  |  |  |  |
| Underweight | 83 (2.4) | 8 (1.3) | 12 (2.7) | 63 (2.6) |
| Healthy weight | 1484 (42.2) | 141 (22.2) | 184 (41.9) | 1159 (47.5) |
| Overweight | 842 (24.0) | 136 (21.4) | 115 (26.2) | 591 (24.2) |
| Obesity | 1104 (31.4) | 350 (55.1) | 128 (29.2) | 626 (25.7) |
| **BMI at age 45 (N = 1,714):** |  |  |  |  |
| Mean ± SD | 29.2 ± 9.3 | 33.6 ± 12.2 | 29.9 ± 9.0 | 28.2 ± 8.4 |
| n (%)^b^ |  |  |  |  |
| Underweight/Healthy weight^c^ | 676 (39.5) | 50 (20.1) | 95 (38.6) | 534 (43.2) |
| Overweight | 437 (25.5) | 57 (24.4) | 56 (22.8) | 324 (26.3) |
| Obesity | 601 (35.1) | 130 (55.6) | 95 (38.6) | 376 (30.5) |
| **BMI at age 55 (N = 651):** |  |  |  |  |
| Mean ± SD | 28.2 ± 8.6 | 31.3 ± 8.7 | 28.6 ± 7.5 | 27.7 ± 8.8 |
| n (%)^b^ |  |  |  |  |
| Underweight/Healthy weight^c^ | 263 (40.4) | 16 (23.5) | 39 (37.5) | 208 (43.4) |
| Overweight | 187 (28.7) | 20 (29.4) | 37 (35.6) | 130 (27.1) |
| Obesity | 201 (30.9) | 32 (47.1) | 28 (26.9) | 141 (29.4) |

Abbreviations: BMI, body mass index; CDC, Center for Disease Control and Prevention; SD, standard deviation.

^a^ Numbers and percentages may not add up to total N or 100% due to missingness.

^b^ BMI cutoff values used for Asians/Asian Americans: underweight (<18.5 kg/m^2^), healthy weight (18.5-22.9 kg/m^2^), overweight (23.0-26.9 kg/m^2^), obesity (≥27.0 kg/m^2^); BMI cutoff values used for other race/ethnicity groups: underweight (<18.5 kg/m^2^), healthy weight (18.5-24.9 kg/m^2^), overweight (25.0-29.9 kg/m^2^), obesity (≥30.0 kg/m^2^). References: (1) Hsu WC et al. BMI cut points to identify at-risk Asian Americans for type 2 diabetes screening. Diabetes Care. 2015 Jan;38(1):150-8. (2) Joslin Diabetes Center AADI. Asian BMI Calculator. Accessed April 14, 2025. <https://aadi.joslin.org/en/am-i-at-risk/asian-bmi-calculator>

^c^ Categories aggregated due to small numbers in the relevant underweight category.

**Supplemental Table S4.** Self-reported baseline lifestyle characteristics using number (percentages), stratified by PCOS/possible PCOS/non-PCOS groups among Apple Women’s Health Study participants enrolled from 11/14/2019 to 12/14/2024 and responded to the relevant survey questions.

| **Self-reported baseline lifestyle characteristics^a^** | **PCOS**  **(N = 11,022)** | **Possible PCOS**  **(N = 7,152)** | **No PCOS**  **(N = 32,183)** | **P-value** |
| --- | --- | --- | --- | --- |
| **Special diets:** |  |  |  |  |
| No special diet, n (%)^b^ | 6311 (59.1) | 4598 (65.0) | 20628 (64.9) | <.0001 |
| Low calorie, n (%)^b^ | 903 (8.5) | 531 (7.5) | 2392 (7.5) | <.001 |
| Low carb, n (%)^b^ | 1685 (15.8) | 650 (9.2) | 3230 (10.2) | <.0001 |
| Low fat, n (%)^b^ | 478 (4.5) | 287 (4.1) | 1292 (4.1) | .005 |
| High fat, n (%)^b^ | 192 (1.8) | 70 (1.0) | 408 (1.3) | <.0001 |
| High protein, n (%)^b^ | 1316 (12.3) | 599 (8.5) | 2798 (8.8) | <.0001 |
| Low sodium, n (%)^b^ | 491 (4.6) | 271 (3.8) | 1107 (3.5) | <.0001 |
| Vegetarian, n (%)^b^ | 469 (4.4) | 361 (5.1) | 1807 (5.7) | <.0001 |
| Vegan, n (%)^b^ | 155 (1.5) | 107 (1.5) | 680 (2.1) | <.0001 |
| No gluten, n (%)^b^ | 744 (7.0) | 375 (5.3) | 1646 (5.2) | <.0001 |
| No dairy, n (%)^b^ | 813 (7.6) | 461 (6.5) | 1724 (5.4) | <.0001 |
| Other special diet, n (%)^b^ | 903 (8.5) | 560 (7.9) | 2247 (7.1) | <.0001 |
| Frequency of eating fruits and vegetables, n (%)^c^ |  |  |  | <.0001 |
| <3 times/week | 2395 (23.3) | 1653 (24.0) | 6304 (20.5) |  |
| 4-7 times/week | 3970 (38.6) | 2654 (38.5) | 11483 (37.3) |  |
| 8-14 times/week | 2410 (23.4) | 1604 (23.3) | 7703 (25.0) |  |
| ≥15 times/week | 1511 (14.7) | 978 (14.2) | 5276 (17.1) |  |
| **Physical activity:** |  |  |  |  |
| Overall physical activity level: no activity, n (%)^d^ | 405 (3.8) | 210 (3.0) | 770 (2.4) | <.0001 |
| Overall physical activity level: light, n (%)^d^ | 5136 (48.0) | 3019 (42.6) | 12086 (38.0) | <.0001 |
| Overall physical activity level: moderate, n (%)^d^ | 4760 (44.4) | 3282 (46.3) | 14706 (46.2) | .008 |
| Overall physical activity level: vigorous, n (%)^d^ | 2106 (19.7) | 1791 (25.3) | 9320 (29.3) | <.0001 |
| Overall physical activity level: strenuous, n (%)^d^ | 222 (2.1) | 216 (3.1) | 1115 (3.5) | <.0001 |
| Exercise minutes per week (any moderate to vigorous leisure time activity), n (%) |  |  |  | <.0001 |
| None | 1057 (9.9) | 586 (8.3) | 2263 (7.1) |  |
| 1-75 | 4717 (44.2) | 2848 (40.3) | 11610 (36.5) |  |
| 76-150 | 2768 (25.9) | 1824 (25.8) | 8388 (26.4) |  |
| 151-300 | 1457 (13.6) | 1170 (16.6) | 6328 (19.9) |  |
| >300 | 684 (6.4) | 641 (9.1) | 3192 (10.0) |  |
| **Stress:** |  |  |  |  |
| PSS score, mean ± SD^e^ | 7.5 ± 3.6 | 7.3 ± 3.7 | 6.3 ± 3.6 | <.0001 |
| PSS score, median (IQR)^e^ | 8 (5 – 10) | 7 (5 – 10) | 6 (3 – 9) |  |
| PSS score > median (7), n (%)^e^ | 5413 (50.7) | 3445 (48.8) | 11891 (37.4) | <.0001 |
| **Sleep:** |  |  |  |  |
| Typical sleep duration on weekdays/work days, n (%) |  |  |  | <.0001 |
| <7 hours/night | 1591 (14.8) | 1004 (14.2) | 3967 (12.5) |  |
| 7-9 hours/night | 7884 (73.5) | 5235 (73.9) | 24612 (77.3) |  |
| >9 hours/night | 1252 (11.7) | 846 (11.9) | 3265 (10.3) |  |
| Typical sleep duration on weekends/non-work days, n (%) |  |  |  | <.0001 |
| <7 hours/night | 846 (7.9) | 486 (6.9) | 1882 (5.9) |  |
| 7-9 hours/night | 6657 (62.1) | 4497 (63.5) | 21180 (66.5) |  |
| >9 hours/night | 3224 (30.1) | 2102 (29.7) | 8782 (27.6) |  |
| **Sleep apnea:** |  |  |  |  |
| Reported physician diagnosed sleep apnea, n (%) | 1611 (14.7) | 689 (9.6) | 2112 (6.6) | <.0001 |
| Reported frequency of snort, gasp, or stop breathing while asleep in the past 12 months, n (%) |  |  |  | <.0001 |
| Never | 4789 (44.7) | 3457 (48.8) | 17862 (56.1) |  |
| Rarely (1-2 nights/week) | 1346 (12.6) | 803 (11.3) | 2993 (9.4) |  |
| Occasionally (3-4 nights/week) | 650 (6.1) | 332 (4.7) | 1142 (3.6) |  |
| Frequently (≥5 nights) | 744 (6.9) | 323 (4.6) | 1060 (3.3) |  |
| Don’t know | 3184 (29.7) | 2167 (30.6) | 8778 (27.6) |  |
| **Substance use:** |  |  |  |  |
| Smoking status, n (%) |  |  |  | <.0001 |
| Never | 6749 (64.5) | 4607 (66.7) | 21245 (68.0) |  |
| Past smoker | 2336 (22.3) | 1476 (21.4) | 6758 (21.6) |  |
| Current smoker | 1383 (13.2) | 825 (11.9) | 3232 (10.3) |  |
| Marijuana use, n (%) |  |  |  | <.0001 |
| Not at all | 7184 (68.2) | 4721 (67.8) | 22779 (72.5) |  |
| Some days | 1882 (17.9) | 1267 (18.2) | 5181 (16.5) |  |
| Every day | 1467 (13.9) | 977 (14.0) | 3458 (11.0) |  |
| E-cigarette use, n (%) |  |  |  | <.0001 |
| Not at all | 8729 (81.7) | 5697 (80.8) | 27304 (85.9) |  |
| Some days | 654 (6.1) | 376 (5.3) | 1510 (4.8) |  |
| Every day | 1296 (12.1) | 978 (13.9) | 2956 (9.3) |  |
| Alcohol frequency in the past year, n (%) |  |  |  | <.0001 |
| Never | 1255 (13.4) | 758 (12.2) | 3303 (11.9) |  |
| Monthly or less | 4290 (45.9) | 2467 (39.7) | 10150 (36.5) |  |
| 2-4 times a month | 2158 (23.1) | 1550 (24.9) | 6909 (24.8) |  |
| 2-3 times a week | 997 (10.7) | 843 (13.6) | 4355 (15.7) |  |
| ≥4 times a week | 641 (6.9) | 600 (9.6) | 3088 (11.1) |  |
| Among those ever had alcohol, number of drinks on a typical drinking day, n (%) |  |  |  | .007 |
| 1 or 2 | 5357 (72.1) | 3567 (70.4) | 16279 (72.5) |  |
| 3 or 4 | 1531 (20.6) | 1088 (21.5) | 4565 (20.3) |  |
| 5 or 6 | 382 (5.1) | 299 (5.9) | 1193 (5.3) |  |
| 7 to 9 | 108 (1.5) | 73 (1.4) | 266 (1.2) |  |
| 10 or more | 56 (0.8) | 38 (0.8) | 139 (0.6) |  |
| Combined information from frequency and number of drinks, n (%) |  |  |  | <.0001 |
| No or light alcohol consumption | 6361 (72.6) | 3864 (65.8) | 16665 (64.0) |  |
| Light consistent drinker or rare binge drinker | 1202 (13.7) | 951 (16.2) | 4394 (16.9) |  |
| Moderate-to-heavy consistent drinker or frequent binge drinker | 1196 (13.7) | 1059 (18.0) | 4967 (19.1) |  |

Abbreviations: PCOS, polycystic ovary syndrome; SD, standard deviation; PSS, perceieved stress scale.

^a^Percentages for each variable calculated based on non-missing data. Denominators may be different from total N. The “don’t know” response for the reported frequency of snort, gasp, or stop breathing while asleep in the past 12 months was preserved and shown as its own category due to its relevance.

^b^No special diet was an exclusive option. If not selecting this option, participants could select all that apply for each of the special diet types listed.

^c^Survey question “How frequently did you eat fruits and vegetables in the past calendar month?” was given to participants, with options presented in this table.

^d^No physical activity was an exclusive option. If not selecting this option, participants could select all that apply for each of the overall physical activity levels listed.

^e^PSS-4 derived from 4 standardized questions, “In the last month, how often have you felt that you were unable to control the important things in your life?”, “In the last month, how often have you felt confident about your ability to handle your personal problems?”, “In the last month, how often have you felt that things were going your way?”, “In the last month, how often have you felt difficulties were piling up so high that you could not overcome them?”, with minimum value of 0 and maximum value of 16.

**Supplemental Table S5.** Comparison of baseline sociodemographic and health characteristics of AWHS participants in three analytical subsets representative of participants that completed relevant surveys used to define possible PCOS status (Figure S1 flowchart).

| **Self-reported sociodemographic and health characteristics at baseline^a^** | **Data subsets from eligible participants who responded to demographics survey and medical history survey** | | |
| --- | --- | --- | --- |
|  | **Subset 1:**  **N = 75,931 participants without a PCOS diagnosis** | **Subset 2:**  **N = 72,533 participants who responded to the reproductive history survey** | **Subset 3:**  **N = 41,197 participants who responded to the reproductive history and hormonal symptoms surveys** |
| **N** | **75,931** | **72,533** | **41,197** |
| *From the demographics survey:* | | | |
| Age, years, median (IQR) | 33 (26 – 42) | 33 (26 – 42) | 35 (27 – 44) |
| Age category, n (%) |  |  |  |
| 18-19 | 3827 (5.0) | 3646 (5.0) | 1740 (4.2) |
| 20-29 | 24463 (32.2) | 23278 (32.1) | 11774 (28.6) |
| 30-39 | 23085 (30.4) | 22029 (30.4) | 12550 (30.5) |
| 40-49 | 15573 (20.5) | 14961 (20.6) | 9360 (22.7) |
| ≥50 | 8983 (11.8) | 8619 (11.9) | 5773 (14.0) |
| Race and ethnicity,^b^ n (%) |  |  |  |
| Non-Hispanic White | 54621 (71.9) | 52359 (72.0) | 30679 (74.5) |
| Non-Hispanic Black | 4109 (5.4) | 3884 (5.4) | 2046 (5.0) |
| Asian | 2438 (3.2) | 2311 (3.2) | 1161 (2.8) |
| Hispanic | 5283 (7.0) | 4967 (6.8) | 2526 (6.1) |
| Multiple/other races | 9231 (12.2) | 8777 (12.1) | 4664 (11.3) |
| Education level, n (%) |  |  |  |
| High school or below | 13581 (17.9) | 12942 (17.8) | 6363 (15.4) |
| Some college or tech school | 23970 (31.6) | 22802 (31.4) | 12454 (30.2) |
| College degree | 21512 (28.3) | 20530 (28.3) | 12186 (29.6) |
| Graduate degree | 16484 (21.7) | 15895 (21.9) | 10023 (24.3) |
| Subjective socioeconomic status (SES), n (%) |  |  |  |
| Low (0-3) | 20636 (27.2) | 19690 (27.1) | 10480 (25.4) |
| Medium (4-5) | 31422 (41.4) | 30007 (41.1) | 17037 (41.4) |
| High (6-9) | 23658 (31.2) | 22642 (31.2) | 13591 (33.0) |
| Reported family history of PCOS, n (%) | 3965 (5.2) | 3816 (5.3) | 2180 (5.3) |
| Body mass index, kg/m^2^, median (IQR) | 26.6 (22.8 - 32.4) | 26.6 (22.8 - 32.4) | 26.8 (22.9 - 32.4) |
| *From the reproductive history survey (variable relevant to one of the criteria for defining possible PCOS):* | | | |
| Prolonged time from menarche to cycle regularity (5+ years, not yet regular, or regular after hormones), n (%) | / (not applicable) | 17059 (23.5) | 9519 (23.1) |

Abbreviations: AWHS, Apple Women’s Health Study; PCOS, polycystic ovary syndrome; IQR, interquartile range.

^a^ Numbers and percentages may not add up to total N or 100% due to missingness. Missingness rates were <1% for sociodemographic characteristics, and <2% for body mass index at baseline and family history of PCOS.

^b^ Those who self-identified categories of “American Indian or Alaska Native”, “Middle Eastern or North African”, “Native Hawaiian or Pacific Islander”, or “none of these fully describe me” were collapsed as “other races” due to relatively small sample sizes; Multiple races included those who self-identified more than one option.

**Supplemental Table S6.** Self-reported sociodemographic and overall health characteristics using number (percentages), stratified by age groups among our analytical sample (with or without PCOS or possible PCOS).

| **Baseline characteristics^a^** | **Age 18-19** | **Age 20-29** | **Age 30-39** | **Age 40-49** | **Age ≥50** |
| --- | --- | --- | --- | --- | --- |
| **N** | 1837 | 14420 | 16244 | 11237 | 6209 |
| Race and ethnicity,^b^ n (%) |  |  |  |  |  |
| Non-Hispanic White | 1257 (68.4) | 10265 (71.2) | 11976 (73.7) | 8464 (75.3) | 5128 (82.6) |
| Non-Hispanic Black | 79 (4.3) | 555 (3.8) | 793 (4.9) | 584 (5.2) | 284 (4.6) |
| Asian | 63 (3.4) | 541 (3.8) | 474 (2.9) | 270 (2.4) | 85 (1.4) |
| Hispanic | 130 (7.1) | 1032 (7.2) | 1094 (6.7) | 675 (6.0) | 230 (3.7) |
| Multiple/other races | 304 (16.5) | 1994 (13.8) | 1861 (11.5) | 1208 (10.8) | 465 (7.5) |
| Education level, n (%) |  |  |  |  |  |
| High school or below | 1194 (65.0) | 2541 (17.6) | 1866 (11.5) | 1174 (10.4) | 511 (8.2) |
| Some college or tech school | 576 (31.4) | 4957 (34.4) | 5087 (31.3) | 3434 (30.6) | 1713 (27.6) |
| College degree or higher | 34 (1.8) | 6858 (47.6) | 9256 (57.0) | 6607 (58.8) | 3978 (64.1) |
| Subjective socioeconomic status (SES), n (%) |  |  |  |  |  |
| Low (0-3) | 599 (32.6) | 4862 (33.7) | 4464 (27.5) | 2508 (22.3) | 868 (14.0) |
| Medium (4-5) | 764 (41.6) | 6233 (43.2) | 6991 (43.0) | 4676 (41.6) | 2191 (35.3) |
| High (6-9) | 470 (25.6) | 3303 (22.9) | 4762 (29.3) | 4028 (35.8) | 3135 (50.5) |
| Self-evaluated overall health compared to other peers, n (%) |  |  |  |  |  |
| Much better | 106 (5.8) | 706 (4.9) | 1071 (6.6) | 1323 (11.8) | 1540 (24.8) |
| Slightly better | 334 (18.2) | 2412 (16.7) | 3089 (19.0) | 2519 (22.4) | 1560 (25.1) |
| About the same | 692 (37.7) | 5521 (38.3) | 5778 (35.6) | 3489 (31.0) | 1425 (23.0) |
| Slightly worse | 527 (28.7) | 3873 (26.9) | 3935 (24.2) | 2362 (21.0) | 936 (15.1) |
| Much worse | 116 (6.3) | 940 (6.5) | 1014 (6.2) | 776 (6.9) | 358 (5.8) |

^a^ Numbers and percentages may not add up to total N or 100% due to missingness. Missingness rates were <1% for sociodemographic characteristics, and <2% for body mass index at baseline and family history of PCOS.

^b^ Those who self-identified categories of “American Indian or Alaska Native”, “Middle Eastern or North African”, “Native Hawaiian or Pacific Islander”, or “none of these fully describe me” were collapsed as “other races” due to relatively small sample sizes; Multiple races included those who self-identified more than one option.

# Supplemental Table S7. Selected lab biomarker concentrations by PCOS status, among subsets of participants within our analytical sample with lab biomarker data from linked clinical health records.

| **Biomarker^a^** | **LOINC code** | **Summary distributions** | **All** | **By PCOS status** | | |
| --- | --- | --- | --- | --- | --- | --- |
|  |  |  |  | **PCOS** | **Possible PCOS** | **No PCOS** |
| Total T in serum/plasma | 2986-8 | *n* | *158* | *72* | *31* | *55* |
|  |  | median [IQR], ng/dL | 31.5 [21.0-56.7] | 36.0 [24.8-52.3] | 31.0 [21.5-95.0] | 26.0 [18.0-45.4] |
| Free T in serum/plasma | 2991-8 | *n* | *76* | *38* | *8* | *30* |
|  |  | median [IQR], pg/mL | 2.8 [1.4-5.2] | 2.1 [2.1-6.8] | 3.6 [1.7-6.9] | 1.6 [1.2-3.0] |
| FSH in serum/plasma | 15067-2  20433-9 | *n* | *174* | *47* | *29* | *98* |
|  |  | median [IQR], mIU/mL | 6.6 [4.7-12.0] | 6.0 [4.2-7.0] | 7.0 [4.6-12.9] | 7.2 [5.1-25] |
| LH in serum/plasma | 10501-5 | *n* | *92* | *39* | *9* | *44* |
|  |  | median [IQR], mIU/mL | 6.8 [4.1-17] | 8.3 [4.8-16] | 6.3 [5.0-35] | 5.6 [3.9-13] |
| E2 in serum/plasma | 2243-4  83096-8 | *n* | *156* | *44* | *24* | *88* |
|  |  | median [IQR], pg/mL | 60 [33-119] | 55 [35-99] | 44 [24-84] | 73 [39-126] |
| TSH in serum/plasma | 3016-3 | *n* | *705* | *456* | *94* | *455* |
|  |  | median [IQR], µIU/mL | 2.0 [1.2-3.3] | 1.8 [1.2-2.9] | 2.1 [1.3-4.5] | 2.0 [1.2-3.4] |
| Prolactin in serum/plasma | 2842-3  20568-2 | *n* | *148* | *64* | *20* | *64* |
|  |  | median [IQR], ng/mL | 11.7 [7.5-16.7] | 11.8 [7.5-16.7] | 11.7 [6.7-15.4] | 11.7 [8.1-17.6] |
| 17-OHP in serum/plasma | 1668-3 | *n* | *58* | *34* | *8* | *16* |
|  |  | median [IQR], ng/dL | 50 [33-75] | 53 [31-77] | 45 [37-66] | 39 [30-63] |
| DHEA-S in serum/plasma | 2191-5 | *n* | *81* | *42* | *7* | *32* |
|  |  | median [IQR], µg/dL | 191 [110-277] | 236 [121-359] | 201 [137-242] | 162 [106-227] |
| HbA1c in blood | 4548-4  17856-6  4549-2 | *n* | *883* | *252* | *126* | *505* |
|  |  | median [IQR], % total hemoglobin | 5.4 [5.1-5.8] | 5.5 [5.2-6.1] | 5.5 [5.1-5.8] | 5.4 [5.1-5.7] |
| HDL in serum/plasma | 2085-9 | n | 547 | 113 | 88 | 346 |
|  |  | median [IQR], mg/dL | 53 [45-64] | 49 [42-62] | 50 [43-63] | 55 [46-65] |

Abbreviations: PCOS, polycystic ovary syndrome; AWHS, Apple Women’s Health Study; LOINC, Logical Observation Identifiers Names and Codes; IQR, interquartile range; T, testosterone; FSH, follicle-stimulating hormone; LH, luteinizing hormone; E2, estradiol; TSH, thyroid-stimulating hormone, also known as thyrotropin; 17-OHP, 17-hydroxyprogesterone; DHEA-S, dehydroepiandrosterone sulfate; HbA1c, hemoglobin A1c; HDL, high-density lipoprotein.

^a^ Biomarkers selected based on relevance and data availability in this cohort.
